# Supplementary material for: Monomeric agonist peptide/MHCII complexes activate T‐cells in an autonomous fashion
Source: EMBO Rep. 2023 Sep 28;24(11):e57842. doi: 10.15252/embr.202357842 (PMC10626418; doi:10.15252/embr.202357842)
Supplement: Supplementary file 1 — Appendix [file EMBR-24-e57842-s006.pdf]

TABLE OF CONTENT

Appendix Figure S1 - Synthesis and testing of I-E^k-reactive 14.4.4 scF_V variants. ……………………3

Appendix Figure S2 - Flow cytometric analysis of TNF-α and IFN-γ induction in T-cells responding to MCC peptide-pulsed BMDCs in the presence of the 14.4.4 scF_V (including reference curve of AF647 counting beads). ………………………………………………………………………………....5

Appendix Figure S3 - Flow cytometric analysis of BMDC, lymph node derived B-cell and T-cell cultures. ………………………………………………………………………………………...…..7

Appendix Figure S4 - Surface I-E^k molecules fail to associate with detergent resistant membranes on non-activated and activated BMDCs and B-cells as shown via lysis gradient centrifugation and flow cytometry. ..…………………………………………………………………...…………...…..9

Appendix Figure S5 - Deconvolved mid sections of BMDCs and B-cells did not show a clustered-appearance of pMHCIIs after staining with the I-E^k-reactive 14.4.4 scF_V or 14.4.4 mAb or the I-A^k-reactive 11-5.2 mAb under native or PFA-fixed conditions. ………………………………...…11

Appendix Figure S6 - Synthesis and testing of mSav A106C and positional accuracy of detected PS-CFP2 signals. ……………………………………………………………………………….……13

Appendix Figure S7 - Quantification of PS-CFP2 blinking, the diffusion of I-E^k on BMDCs and B-cells at 25 °C, the surface-distribution of CD18 on living B-cells and the fluorescent background in the absence of PS-CFP2. …………..…………………………………………..………………….......16

Appendix Figure S8 - Quantification of the surface-distribution of I-E^k, CD18 and CD205 on living and PFA-fixed BMDCs and B-cells. ...…………..……………………………………..…………...19

Appendix Figure S9 - Quantification of the surface-distribution of I-Ek, CD205 and CD18 on PFA-fixed BMDCs and B-cells. ..…………..…………………………………………..………………...22

Appendix Figure S10 - STED microscopy of activated BMDCs confirmed a randomized distribution of pMHCIIs. …………..………………………………………..……………………......………...24

Appendix Figure S11 - STED microscopy of activated B-cells confirmed a randomized distribution of pMHCIIs. ………..……………………………………..…………………...………..……..26

Appendix Figure S12 - Diffusion analysis of I-E^k on non-activated and activated BMDCs and B-cells at 37 °C. ……….………………………………………..……………………...……….…………...28

Appendix Figure S13 - Flow cytometry and diffusion analysis of newly-arriving I-E^k in complex with MCC-PEG$_2$-biotin on activated BMDCs and B-cells. …………………………………………..……….30

Appendix Figure S14 - TOCCSL simulation and quantitation of I-E^k surface densities and FRET measurements of I-E^k-bound 14.4.4 scF$_V$ on BMDCs, B-cells and PLBs. …………………...…..……32

Appendix Figure S15 - Quantitation of synaptic TCR occupancies (the ratio of pMHCII-bound TCRs to total TCRs) via FRET imaging. ..…….…………………………………………...………………35

Appendix Figure S16 - Synaptic TCR-pMHCII binding and immune synapse formation as visualized via TIRF microscopy. ……..…………………………………..…………………………...38

Appendix Figure S17 - Synaptic engagement and recognition of agonist I-E^k/MCC present in low abundance and co-presented "bystander" ligands present in high abundance and calculation of apparent lifetimes from TCR:I-E^k/MCC binding events recorded via smFRET at different time lags. …...…….40

Appendix Figure S18 - Highly abundant bystander pMHCIIs did not sensitize T-cells for low levels of antigenic pMHCIIs. ………………………………………..…………………....……….......42

Appendix Figure S19 - Co-agonist candidates I-E^k/K99A and I-E^k/ER60 supplied in increasing densities did not sensitize T-cells for the detection of agonist I-E^k/MCC. …………………………...45

Appendix Figure S20 - DNA and protein sequence of the 14.4.4 scF$_V$. …..………………………..…47

Appendix Table S1 - Ligand occupancy of DNA origamis, fitting parameters of dose-response curves, and significance table of the indicated constructs. ……………………………………………….………49

Appendix Figure S1.

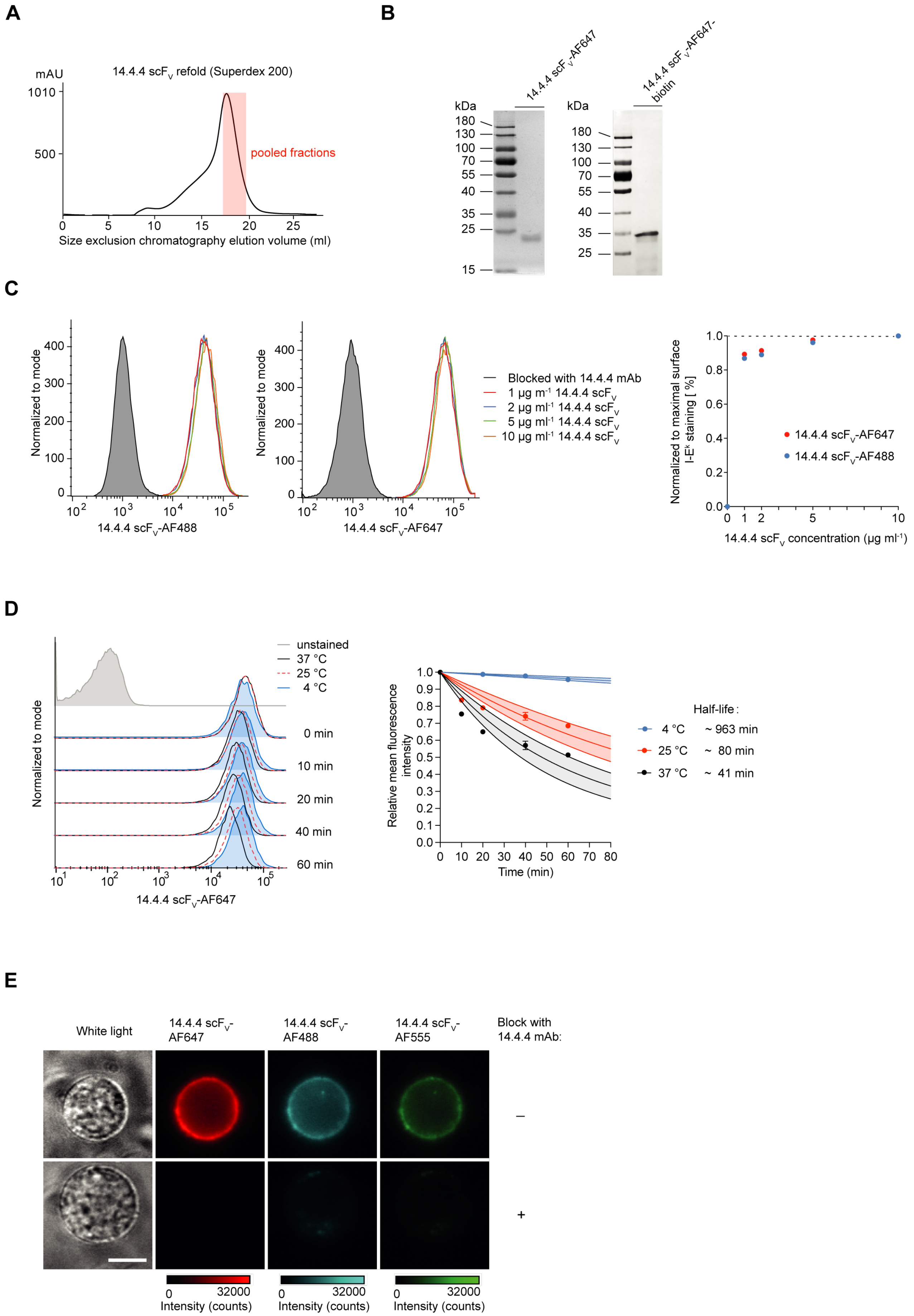

Appendix Figure S1 - Synthesis and testing of I-E^k-reactive 14.4.4 scF$_V$ variants.

A Following refolding of 14.4.4 scF$_V$ from *E. coli* inclusion bodies, Superdex 200 size exclusion chromatography was applied to separate monomeric 14.4.4 scF$_V$ from multimeric conjugates or protein aggregates. To monitor the composition of the major peak of the S200 profile, fraction 31 to 39 representing monomeric 14.4.4 scF$_V$ were pooled and either immediately labeled with lysine- or cysteine-reactive fluorophores or site-specifically biotinylated with the use of BirA biotin ligase.

B Non-reducing, non-boiling 10% SDS-PAGE analysis of 14.4.4-scF$_V$ labeled with NHS-AF647 (colloidal Coomassie, left) and the site-specifically biotinylated and maleimide-AF647-conjugated 14.4.4 scF$_V$ version (14.4.4 scF$_V$-AF647-biotin, silver staining, right).

C I-E^k-expressing CH27 B-cells (n > 15,000 cells per data point) were stained with 1, 2, 5 or 10 μg ml^{-1} site-specifically AF647- or AF488-conjugated 14.4.4 scF$_V$ on ice and analyzed via flow cytometry to determine the concentration of 14.4.4 scF$_V$ allowing for saturating labeling conditions. A concentration of 5 μg ml^{-1} was deemed sufficient to stain 95% of surface expressed I-E^k molecules on 0.25 x 10^6 CH27 B-cells. Single data points were pooled from two technical replicates.

D Flow cytometry analysis to determine the kinetics of unbinding for the 14.4.4 scF$_V$ associated with I-E^k on CH27 B-cells (n > 4,000 cells per condition) at 4°C, 25°C and 37°C. The 14.4.4 scF$_V$ demonstrated a suitable binding stability to perform quantitative imaging experiments with a half-life of ~ 16 hours at 4°C, ~ 80 minutes at 25°C and ~ 41 minutes at 37°C (one-phase decay fit through the first four data points with a shared plateau of 0.1). Data points represent mean and standard deviation of two technical replicates. 95% confidence intervals of the fit are shown.

E To assess specific binding of the 14.4.4 scF$_V$ to I-E^k, we stained CH27 B-cells with a 1:1:1 mix of the 14.4.4 scF$_V$-AF647, 14.4.4 scF$_V$-AF488 and 14.4.4 scF$_V$-AF555. After staining on ice, CH27 B-cells were immediately seeded onto ICAM-1 coated glass slides for investigation via fluorescence microscopy. Epitope blockade with a 14.4.4 mAb completely abrogated 14.4.4 scF$_V$ binding, which confirmed target specificity. Scale bar, 10 μm.

Appendix Figure S2.

A

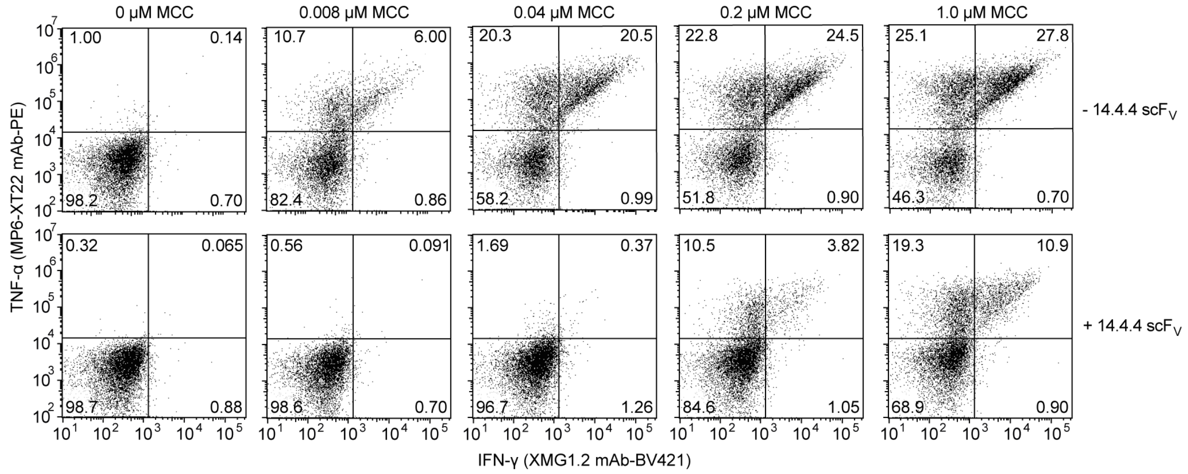

B

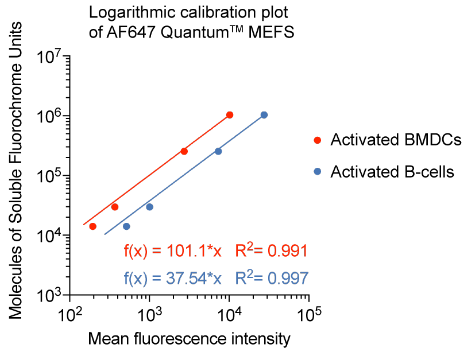

Appendix Figure S2 - Flow cytometric analysis of TNF-α and IFN-γ induction in T-cells responding to MCC peptide-pulsed BMDCs in the presence of the 14.4.4 scF$_V$ (including reference curve of AF647 counting beads).

A Flow cytometry analysis of TNF-α and IFN-γ induction in T-cells co-cultured with activated BMDCs, pulsed with increasing quantities of the 88-103 MCC peptide (from 0 to 1 μM) for 1 hour, and in the presence or absence of 20 μg ml^{-1} 14.4.4 scF$_V$. Cells were pre-gated for single living cells (n > 11,000 per condition) and TCR-expression (H57 mAb-APC-Fire750).

B Example reference curve of AF647 counting beads (AF647 Quantum™ MESF) as recorded via flow cytometry and calculated with an Excel sheet provided by the manufacturer (Bangs Laboratories). A reference curve was determined for each biological replicate in Fig 1B-C.

Appendix Figure S3.

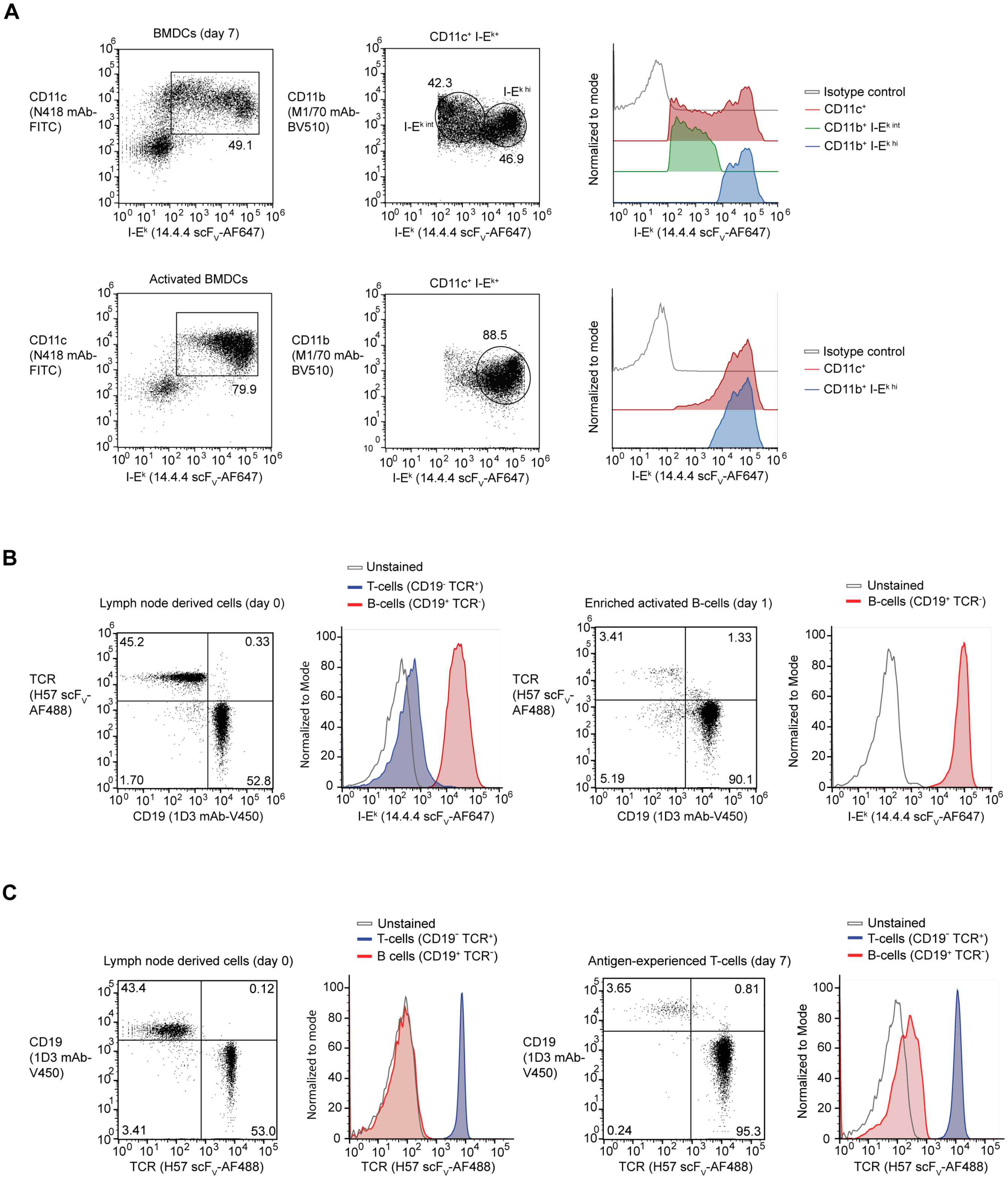

Appendix Figure S3 - Flow cytometric analysis of BMDC, lymph node derived B-cell and T-cell cultures.

A Flow cytometric analysis of GMCSF- and IL-4-stimulated bone marrow-derived dendritic cells (BMDCs) isolated from 5c.c7 TCR-transgenic mice after 7 days in culture. Non-activated BMDCs harvested on day 6 or 7 were stained with CD11c mAb-FITC, CD11b mAb- BV510 and 14.4.4 scF$_V$-AF647 (I-E^k) and gated as CD11c$^+$ CD11b$^+$ I-E^{k+} (upper panel). BMDCs were stimulated with 100 ng ml^{-1} LPS for 24 h to induce BMDC activation (lower panel).

B Flow cytometric analysis of lymph node derived cells isolated from 5c.c7 TCR-transgenic mice. Left panel: Lymphocytes were stained with CD19 mAb-V450, H57 scF$_V$-AF488 (TCRβ chain) and 14.4.4 scF$_V$-AF647 (I-E^k) and gated as CD19$^+$ TCR$^-$ B-cells and CD19$^-$ TCR$^+$ T-cells (left). Right panel: Non-activated B-cell were isolated via (negative) magnetic labeling of lymph node derived cells and depletion of all activated B-cells and non-B-cells. Enriched primary B-cells were stimulated with 50 µg ml^{-1} LPS for 24-48 h (right). The cell population gated as CD19$^+$ TCR$^-$ I-E^{k+} was used to determine the number of surface-expressed I-E^k molecules on B-cells.

C Flow cytometric analysis of lymphocytes obtained from lymph nodes of 5c.c7 TCR-transgenic mice. Lymphocytes were stained with CD19 mAb-V450 and H57 scF$_V$-AF488 (TCRβ chain) and gated as CD19$^+$ TCR$^-$ B-cells and CD19$^-$ TCR$^+$ T-cells. Left panel: T-cells isolated from lymph nodes on day 0 were judged to be a mixed culture containing predominantly B-cells and naïve T-cells. Right panel: Lymphocytes were pulsed with 0.5-1 µM HPLC-purified 88-103 MCC peptide and IL-2 and cultured for 7 days to generate a B-cell depleted antigen-experienced T-cell culture.

Appendix Figure S4.

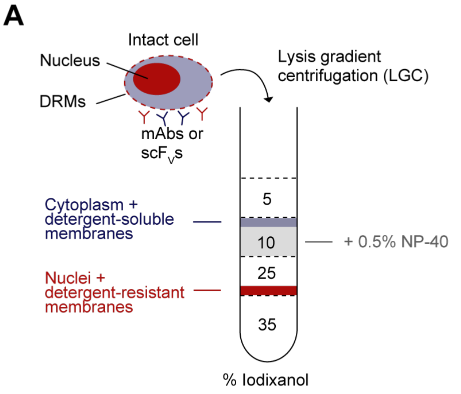

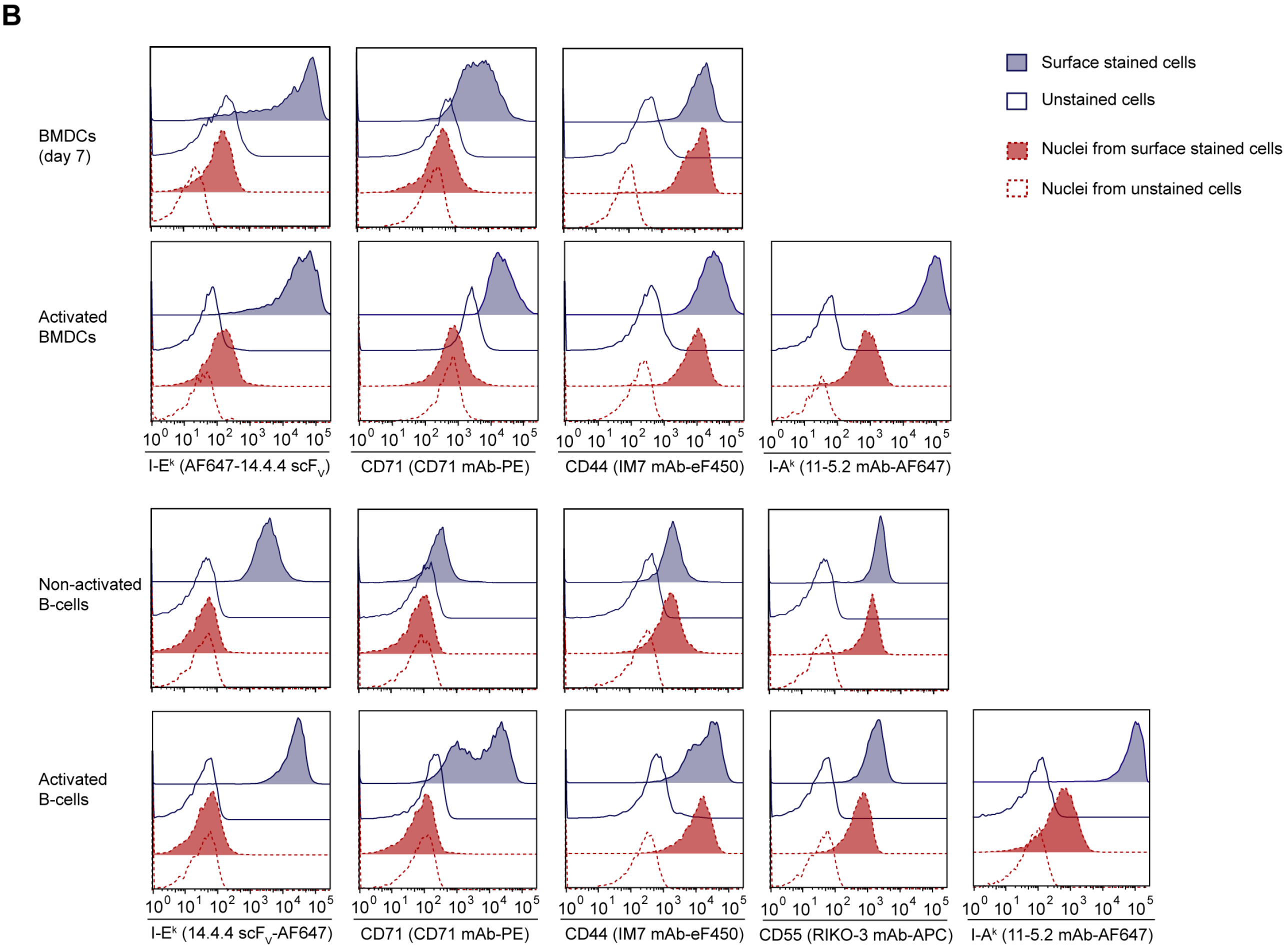

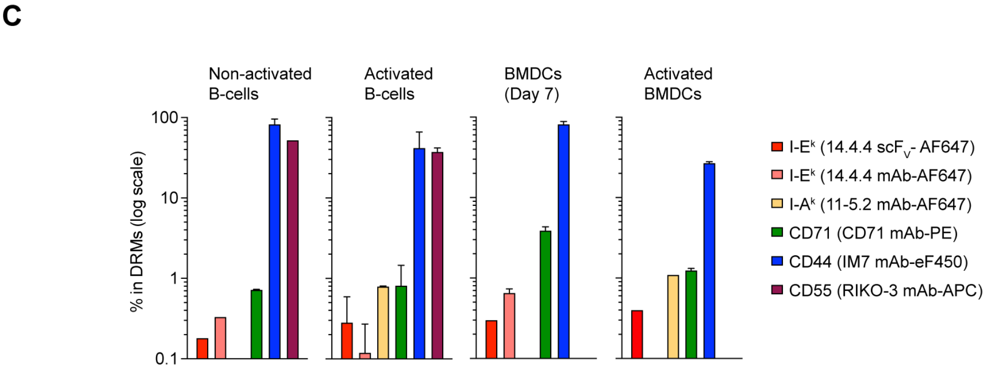

Appendix Figure S4 - Surface I-E^k molecules fail to associate with detergent resistant membranes on non-activated and activated BMDCs and B-cells as shown via lysis gradient centrifugation and flow cytometry.

A Scheme of the lysis gradient centrifugation (LGC) procedure. Non-activated and activated BMDCs or B-cells were stained with scF$_V$s or mAbs on ice and subjected to a quick LGC step in order to separate detergent-resistant membranes (DRMs) and nuclei from cytoplasm and detergent-soluble membranes. Intact cells (n = 2,000 – 11,000) and LGC-isolated nuclei containing DRMs (n = 700 – 14,000) were then analyzed by flow cytometry.

B Activated and non-activated CD11c$^+$ -sorted BMDCs were stained with CD71 mAb-PE, CD44 mAb-eF450, 14.4.4 scF$_V$-AF647, 14.4.4 mAb-AF647 or 11-5.2 mAb-AF647. Activated and non-activated CD19$^+$ B-cells (negatively MACS-sorted, stained with CD19 mAb-FITC) were decorated with, CD71 mAb-PE, CD44 mAb-eF450, 14.4.4 scF$_V$-AF647, 14.4.4 mAb-AF647, 11-5.2 mAb-AF647 and CD55 mAb-APC. The intensity histograms represent intact cells (blue shade) and LGC-isolated nuclei (red shade) next to unstained cells (blue line) and nuclei (red dashed line) as controls for background subtraction.

C Percentages (depicted in log scale) of I-E^k in DRMs on non-activated and activated BMDCs and B-cells as determined by comparing the fluorescence intensity of intact cell and LGC-isolated nuclei staining. CD71 mAb-PE staining was used as negative control (not enriched in DRMs). CD55 and CD44 served as positive controls (partly or fully associated with DRMs). Statistics: mean and standard deviation of one to four biological replicates.

Appendix Figure S5.

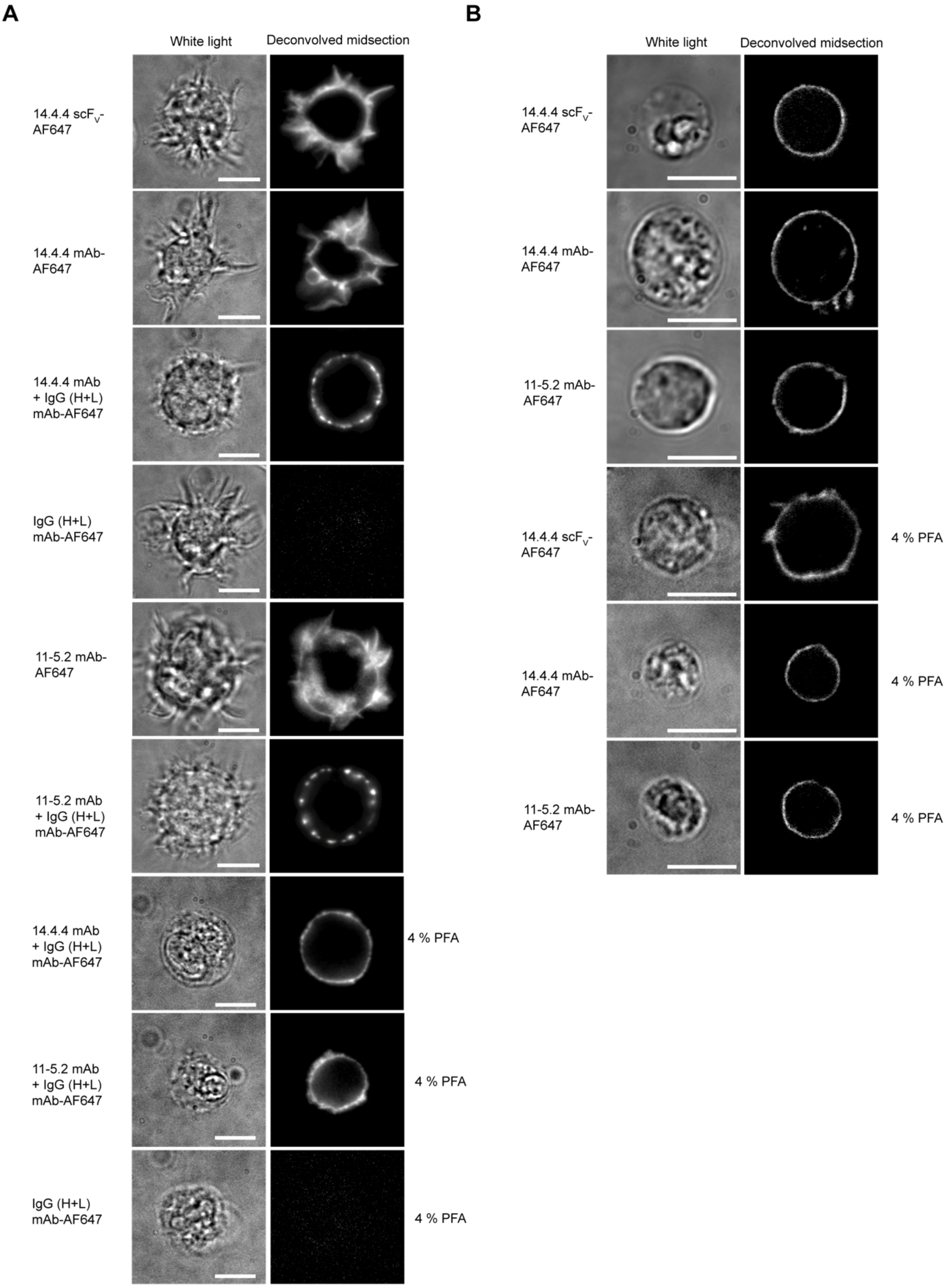

Appendix Figure S5 - Deconvolved mid sections of BMDCs and B-cells did not show a clustered-appearance of pMHCIIs after staining with the I-E^k-reactive 14.4.4 scF$_V$ or 14.4.4 mAb or the I-A^k-reactive 11-5.2 mAb under native or PFA-fixed conditions.

A Deconvolved mid sections of living and PFA-fixed activated BMDCs, which had been stained with the 14.4.4 scF$_V$-AF647 or indicated AF647-conjugated mAbs. Scale bars, 10 µm.

B Deconvolved mid sections of living and PFA-fixed activated B-cells, which had been stained with the 14.4.4 scF$_V$-AF647 or indicated AF647-conjugated mAbs. Scale bars, 10 µm.

Appendix Figure S6.

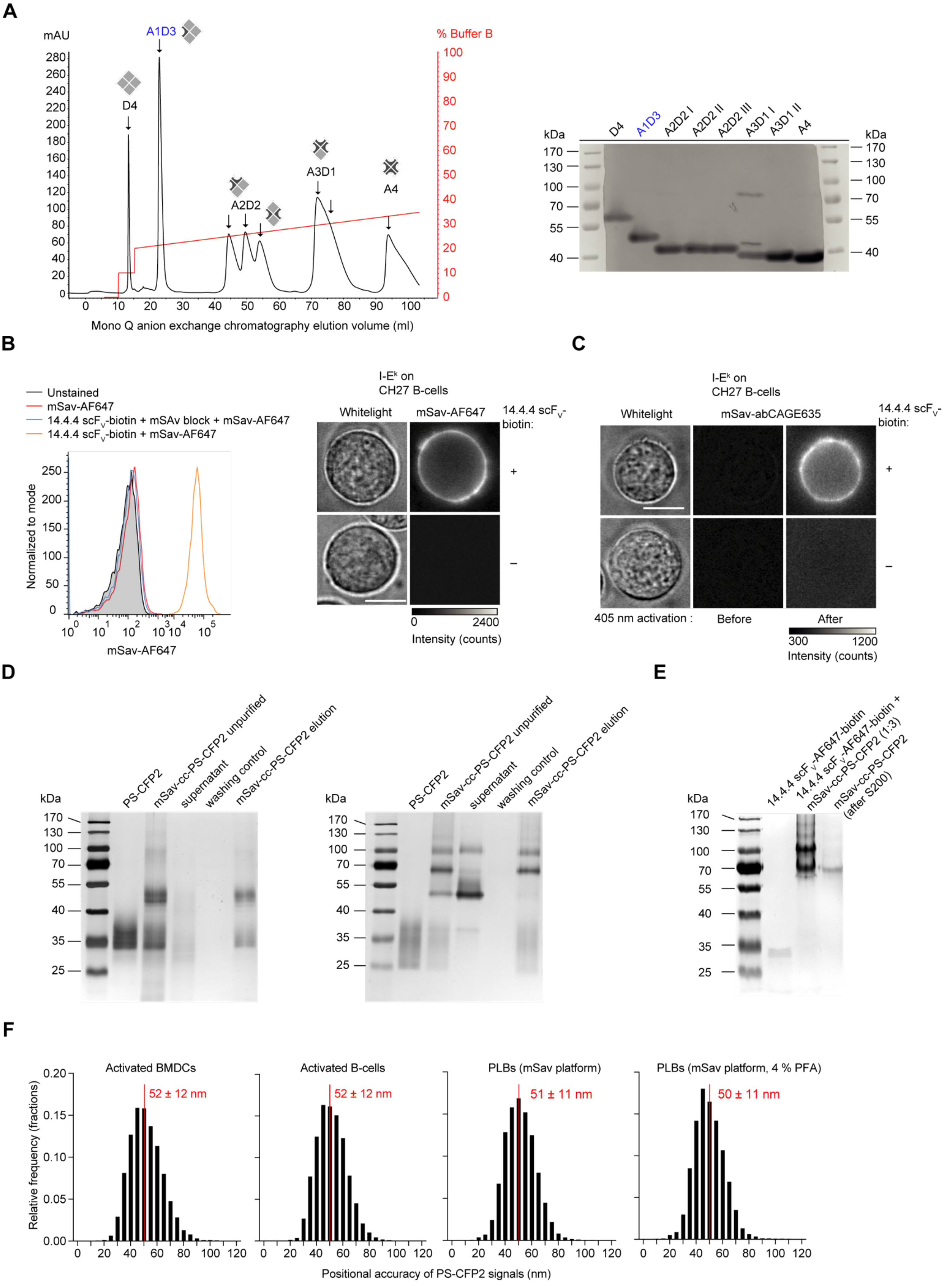

Appendix Figure S6 - Synthesis and testing of mSav A106C and positional accuracy of detected PS-CFP2 signals.

A Monovalent streptavidin containing a free cysteine (A106C) for site-specific labeling (A1D3) could be separated from zero-valent (D4), divalent (A2D2), trivalent (A3D1) or tetravalent (A4) streptavidin based on the C-terminal hexa-glutamate tag located on the "Alive" subunit. Indicated major peak fractions from anion exchange chromatography (MonoQ) were examined by reducing and non-boiling 10% SDS PAGE (silver stain). Due to the increase in negative charge contributed to the hexa-glutamate tags (A4 > D4), streptavidin tetramers migrated more rapidly in SDS-PAGE.

B Flow cytometry and microscopy-based analysis to elucidate specific binding of the site-specifically AF647-conjugated mSav with a biotinylated 14.4.4 scF_V to I-E^k on CH27 B-cells. The absence of the biotinylated probe abolished completely the binding of the mSav-AF647 to the cells. Scale bar, 10 µm.

C To design a photostable and photo-inducible molecular probe for tracking experiments, we site-specifically conjugated the photoactivatable fluorophore Abberior CAGE635 (abCAGE635) to mSav. Functionality of mSav-abCAGE635 was elucidated via fluorescence microscopy. The initially non-fluorescent ("caged") abCAGE635 dye could be activated with a short 405 nm laser pulse. Scale bar, 10 µm.

D The protein construct mSav-cc-PS-CFP2 was produced by chemically crosslinking (via TCO- and tetrazine-based click chemistry) a version of mSav featuring an unpaired single cysteine residue with a cysteine mutant of PS-CFP2. Ni-NTA affinity chromatography was applied to separate the mSav-cc-PS-CFP2 conjugate (which binds to biotin) together with free PS-CFP2-tetrazine (which cannot bind to biotin) from free mSav-TCO based on the 12x histidine tag present on the PS-CFP2-tetrazine protein. 10% SDS-PAGE performed under reducing and boiling (left) or non-reducing and non-boiling conditions (right) followed by silver staining of the protein bands reveal individual steps of the purification process and the eluted mSav-cc-PS-CFP2 conjugate, which was then subjected to S200 gel filtration to remove multimeric complexes.

E Monovalent streptavidin-based biotin shift assay with the mSav-cc-PS-CFP2 conjugate (after S200 gel filtration) confirmed sufficient biotinylation (90-100%) of the site-specifically biotinylated and AF647-conjugated 14.4.4 scF_V (14.4.4 scF_V-AF647-biotin) as analyzed via non-reducing, non-boiling 10% SDS PAGE. Protein bands were visualized with silver staining.

F Positional accuracy of detected PS-CFP2 signals on a living BMDC and B-cell (Fig 2B) and PLBs (in complex with the mSav platform) after fitting with a Gaussian intensity profile via ThunderSTORM. Histograms represent n=5,794 signals on a BMDC, n=13,664 signals on a B-cell, n=15,624 signals on PLBs, n=13,103 signals on PLBs (4% PFA), binning = 5 nm. The red line indicates mean ± standard deviation.

Appendix Figure S7.

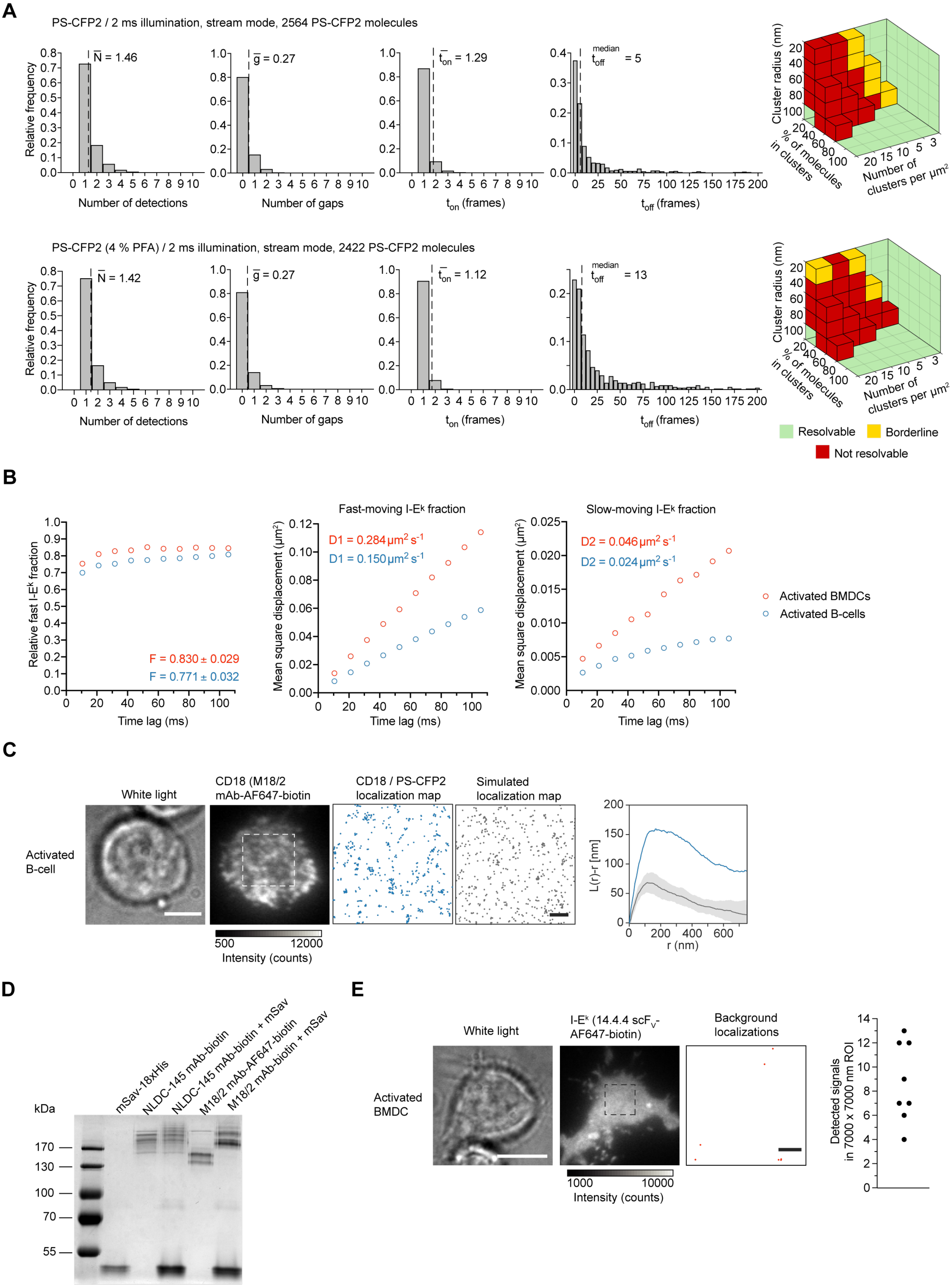

Appendix Figure S7 - Quantification of PS-CFP2 blinking, the diffusion of I-E^k on BMDCs and B-cells at 25 °C, the surface-distribution of CD18 on living B-cells and the fluorescent background in the absence of PS-CFP2.

A The normalized histograms illustrate the four parameters to describe the blinking behavior of the photoactivatable fluorophore PS-CFP2 in the presence or absence of 4% PFA treatment using a mSav-based platform. To keep blinking at low levels, we recorded PS-CFP2 applying continuous illumination of 2 ms intervals (readout time 2.3 ms, stream mode) at a power density of 3.5 kW cm^{-2}. Numbers within the histograms indicate the mean or median value of the respective parameters: N=number of localizations per trace, g = number of gaps per trace, t_{on} = on-time in frames, t_{off} = off-time in frames. In total, n = 2,564 molecules (PS-CFP2) and n = 2,422 molecules (PS-CFP2, 4% PFA) on n = 530 (PS-CFP2) and n = 537 (PS-CFP2, 4% PFA) independent lipid bilayer positions were recorded. PS-CFP2 blinks lasting longer than 30 frames were excluded. Data were pooled from two biological replicates. Cubes represent the sensitivity of Ripley's K analysis in detecting clustering scenarios with the underlying blinking statistic. Ripley's K functions were determined from Monte Carlo simulations of nanoclusters with a cluster radius of 20, 40, 60, or 100 nm, a fraction of molecules residing inside clusters between 20 and 100%, 3, 5, 10, 15, and 20 clusters per µm^2, an average molecular density of 100 molecules per µm^2 and the experimentally determined blinking statistics of PS-CFP2 under PFA-fixed and non-fixed conditions.

B BMDCs and B-cells were decorated with the 14.4.4 scF$_V$-biotin and mSav- abCAGE635 and seeded onto ICAM-1 coated glass slides to record the diffusion of single I-E^k molecules at 25 °C with a time lag of 10.58 ms. Left panel: A binary fit revealed the relative fraction of fast- and slow-moving I-E^k molecules on activated BMDCs and B-cells. Middle panel: Mean square displacement plot of fast-moving I-E^k molecules. Right panel: Mean square displacement plot of slow-moving I-E^k molecules. F = fraction of fast-moving molecules. D1 = diffusion constant of fast-moving molecules. D2 = diffusion constant of slow-moving molecules; Statistics: mean and standard deviation. Number of trajectories n = 28,865 (22 activated BMDCs, 24 h LPS) and n = 24,874 (34 activated B-cells, 48 h LPS) of one experiment.

C Quantification of the surface-distribution of CD18 on living activated B-cells. Ripley's K analysis was employed to compare cell-derived CD18 distributions (blue) to ten simulations of randomized distributions with corresponding molecular densities, diffusion parameters and the PS-CFP2 blinking statistic (gray, mean ± standard deviation). Large scale bar, 5 μm. Small scale bar, 1 μm.

D mSav-association based gel shift assay of the biotinylated and AF647-labeled CD205-reactive mAb NLDC-145 (unpurified) and CD18-reactive mAb M18/2 (purified with a monomeric avidin column) as analyzed via non-reducing, non-boiling 10% SDS PAGE. Protein bands were visualized with colloidal Coomassie brilliant blue staining and indicate one to two biotins per mAb.

E Localization map derived from PALM measurements in the absence of PS-CFP2. Activated BMDCs were decorated only with 14.4.4 scF$_V$-AF647-biotin. The number of localizations detected in the PS-CFP2 emission channel (ET525/50) over 3,050 frames within a 7,000 x 7,000 nm cell ROI is shown. Data are representative for n = 8 cells of one experiment. Large scale bar, 5 μm. Small scale bar, 1 μm.

Appendix Figure S8.

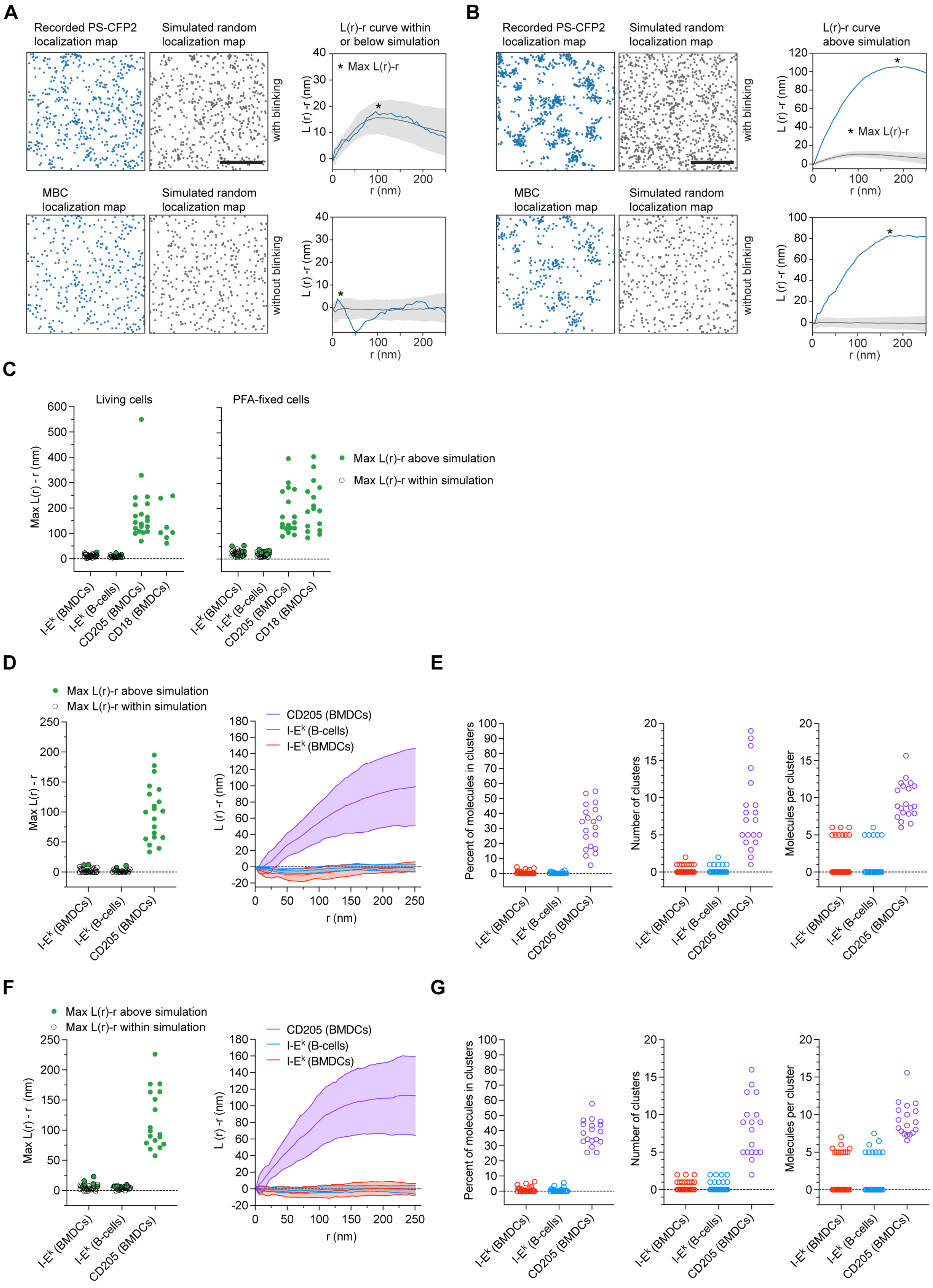

Appendix Figure S8 - Quantification of the surface-distribution of I-E^k and CD205 on living and PFA-fixed BMDCs and B-cells.

A Upper panel: Example of a recorded I-E^k (PS-CFP2) localization map (3,000 x 3,000 nm, blue) next to a simulated random localization map (gray) with the same molecular density, including the blinking dynamics of PS-CFP2 and optionally the diffusion parameters of I-E^k (for live cell analysis). Scale bar, 1 µm. Ripley's K analysis of the recorded cell region (blue) and 100 simulations (mean ± standard deviation) of the respective random distribution (gray) including the blinking dynamics of PS-CFP2. The maximum value of the sample L(r)-r function (blue) is marked with an asterisk. Lower panel: Same as above, but after model-based correction of the recorded I-E^k (PS-CFP2) localization map. The random simulation considers only the molecular density of the MBC localization map (i.e. ground truth without blinking).

B Upper panel: Example of a recorded CD205 (PS-CFP2) localization map (3,000 x 3,000 nm, blue) next to a simulated random localization map (gray) with the same molecular density, including the blinking dynamics of PS-CFP2 and optionally the diffusion parameters of CD205 (for live cell analysis). Scale bar, 1 µm. Ripley's K analysis of the recorded cell region (blue) and 100 simulations (mean ± standard deviation) of the respective random distribution (gray) including the blinking dynamics of PS-CFP2 is shown. The maximum value of the sample L(r)-r function (blue) is marked with an asterisk. Lower panel: Same as above, but after model-based correction of the recorded CD205 (PS-CFP2) localization map. The random simulation considers only the molecular density of the MBC localization map (i.e. ground truth without blinking).

C Summary of the Ripley's K blinking analysis on living and PFA-fixed cell regions. Left panel (living cells): Analysis of 1-2 ROIs (3,000 x 3,000 nm) per cell of n = 14 cells (I-E^k, BMDCs), n = 11 cells (I-E^k, B-cells), n = 10 cells (CD205, BMDCs), and n = 5 cells (CD18, BMDC). Right panel (PFA-fixed cells): Analysis of 1-2 ROIs (3,000 x 3,000 nm) per cell of n = 13 cells (I-E^k, BMDCs), n = 21 cells (I-E^k, B-cells), n = 9 cells (CD205, BMDCs), and n = 9 cells (CD18, BMDC). Data was pooled from one to two independent experiments. Gray circles mark

cell regions with a random appearance of molecules (Max L(r)-r within simulation as described in (A)). Green dots mark cell regions with a clustered appearance of molecules (Max L(r)-r above simulation as described in (B)).

D Left panel: Same as in (C) but after MBC of the ROIs representing living cells. Right panel: Summarized Ripley's K function of I-E^k and CD205 localization maps derived from living BMDCs and B-cells after MBC. Shown are mean (solid line) ± standard deviation (area fill).

E DBSCAN analysis of I-E^k and CD205 localization maps recorded from living BMDCs and B-cells after MBC. Every circle represents a cell region as described in (C).

F Left panel: Same as in (C) but after MBC of the ROIs representing PFA-fixed cells. Right panel: Summarized Ripley's K function of I-E^k and CD205 localization maps derived from PFA-fixed BMDCs and B-cells after MBC. Shown are mean (solid line) ± standard deviation (area fill).

G DBSCAN analysis of I-E^k and CD205 localization maps derived from PFA-fixed BMDCs and B-cells after MBC. Every circle represents a cell region as described in (C).

Appendix Figure S9.

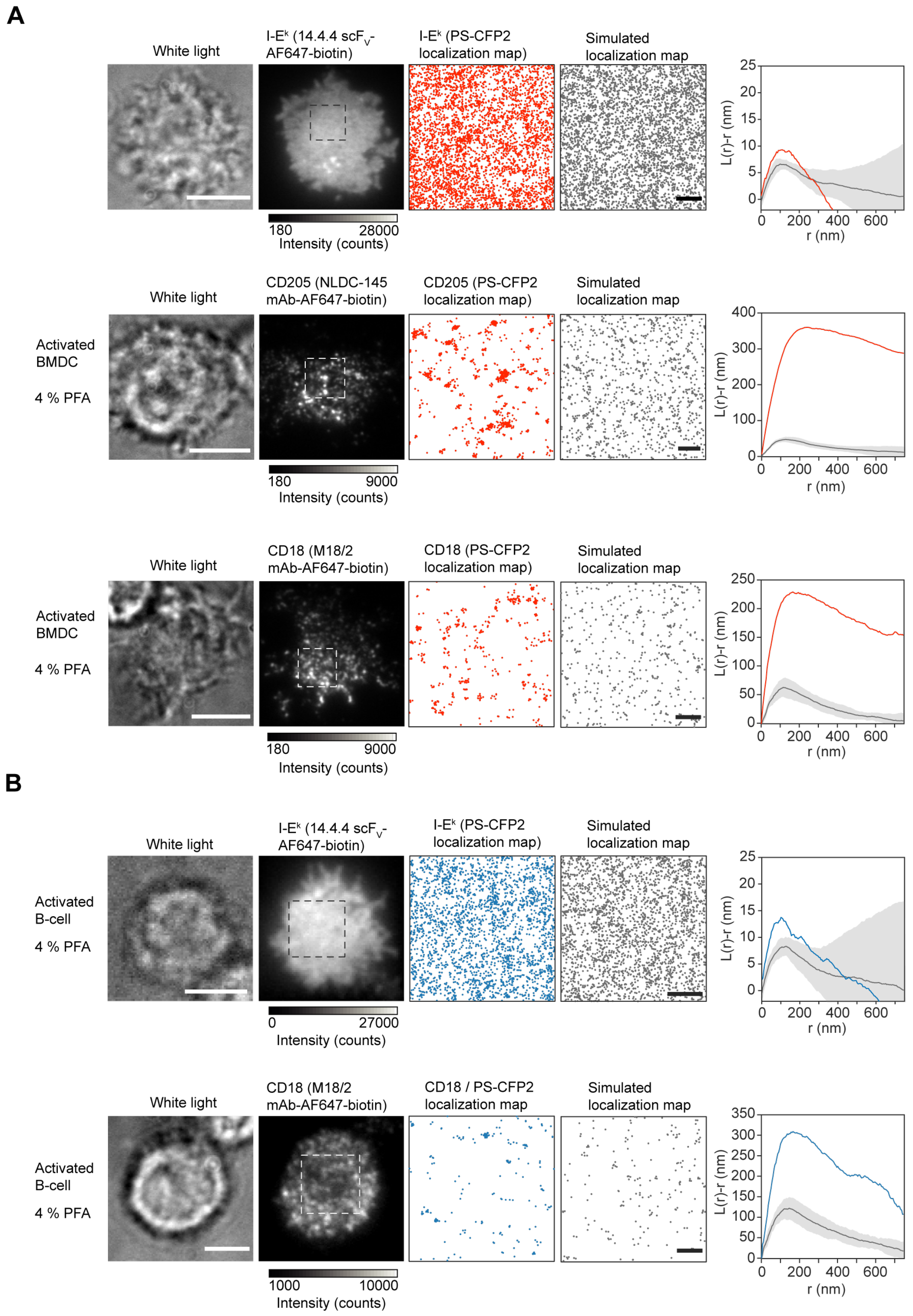

Appendix Figure S9 - Quantification of the surface-distribution of I-E^k, CD205 and CD18 on PFA-fixed BMDCs and B-cells.

A Quantification of the surface-distribution of I-E^k, CD205 and CD18 on PFA-fixed activated BMDCs. Cells were labelled with 14.4.4 scF$_V$-AF647-biotin (I-E^k) or NLDC-145 mAb-AF647-biotin (CD205) or M18/2 mAb-AF647 biotin (CD18) followed by mSav-cc-PS-CFP2, seeded on ICAM-1 coated glass slides, and fixed with 4% PFA prior to recording of the localization map. The presence of each cell was verified through the acquisition of a white light image (first image, scale bar 10 µm), a single AF647 ensemble fluorescence image (second image), followed by recording 3,050 image frames displaying single PS-CFP2 fluorescence events within 7 seconds to be assembled as a localization map (third image), which was compared to a simulated random I-E^k localization map (fourth image, scale bar, 1 µm). Ripley's K analysis was applied to compare cell-derived I-E^k (upper row), CD205 (middle row) or CD18 (lower row) distributions (red) to ten simulations of randomized distributions with corresponding molecular densities and PS-CFP2 blinking statistics (gray, mean ± standard deviation).

B Quantification of the surface-distribution of I-E^k and CD18 on PFA-fixed activated B-cells. B-cells were labeled with 14.4.4 scF$_V$-AF647-biotin (I-E^k) or M18/2 mAb-AF647-biotin (CD18) followed by mSav-cc-PS-CFP2, seeded on ICAM-1 coated glass slides, and fixed with 4% PFA prior to recording of the localization map. Ripley's K analysis was employed to compare cell-derived I-E^k (upper row) or CD18 (lower row) distributions (blue) to ten simulations of randomized distributions with corresponding molecular densities and blinking statistics (gray, mean ± standard deviation). Large scale bar, 5 µm. Small scale bar, 1 µm.

Appendix Figure S10.

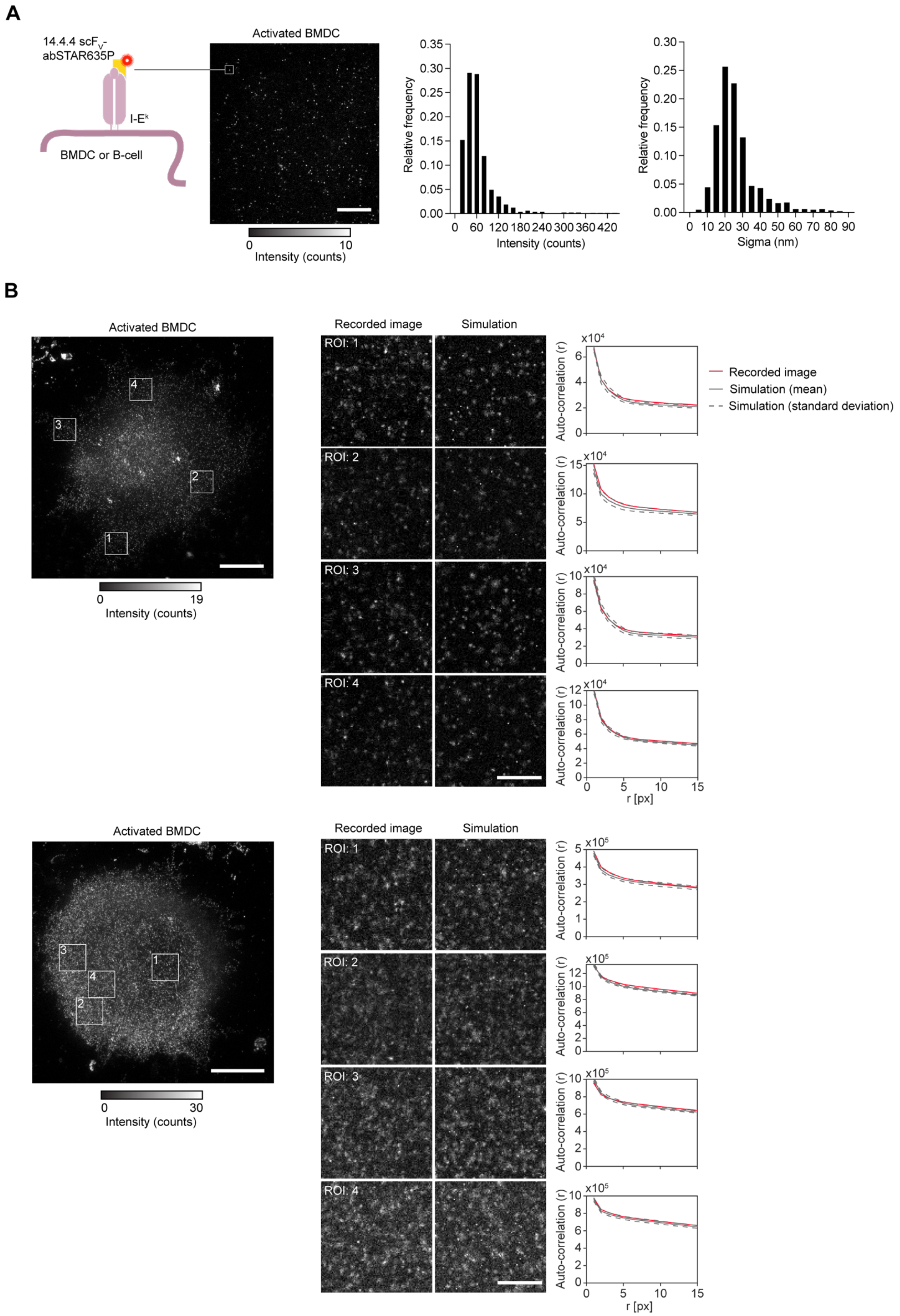

Appendix Figure S10 - STED microscopy of activated BMDCs confirmed a randomized distribution of pMHCIIs.

A Activated BMDCs and B-cells were stained with 14.4.4 scF_V- abSTAR635P and allowed to adhere on ICAM-1 coated glass-slides. Afterwards, the samples were fixed, mounted and subjected to STED microscopy. The images represent the distribution of $I\text{-}E^k$ on a representative activated BMDC labeled with single molecule concentrations of the 14.4.4 scF_V-abSTAR635P. Representing fluorescent spots (n = 786) were localized via the ImageJ plugin ThunderSTORM to calculate the fluorescence intensity and sigma of each individual spot (right). Histograms: binning = 20 (intensity), binning = 5 (sigma). Scale bar, 2.5 μm.

B STED microscopy of activated BMDCs labeled with saturated amounts of 14.4.4 scF_V-abSTAR635P. Images represent STED recordings on cells and simulated STED images of randomly distributed fluorescence molecules with the number of molecules per area and the point-spread function matched to the experimental data. Right panels show the image autocorrelation analysis of each recorded STED image (100 x 100 px ROI, red), as a function of the distance *r* (1 pixel (px) = 25 nm); gray line and gray dotted line indicate the ACF of a reference (mean and standard deviation, respectively), for a random distribution derived from simulations based on the experimentally determined properties of the point-spread function of n = 5 independent simulations. Scale bars, 5 μm (overview) and 1 μm (enlarged ROI).

Appendix Figure S11.

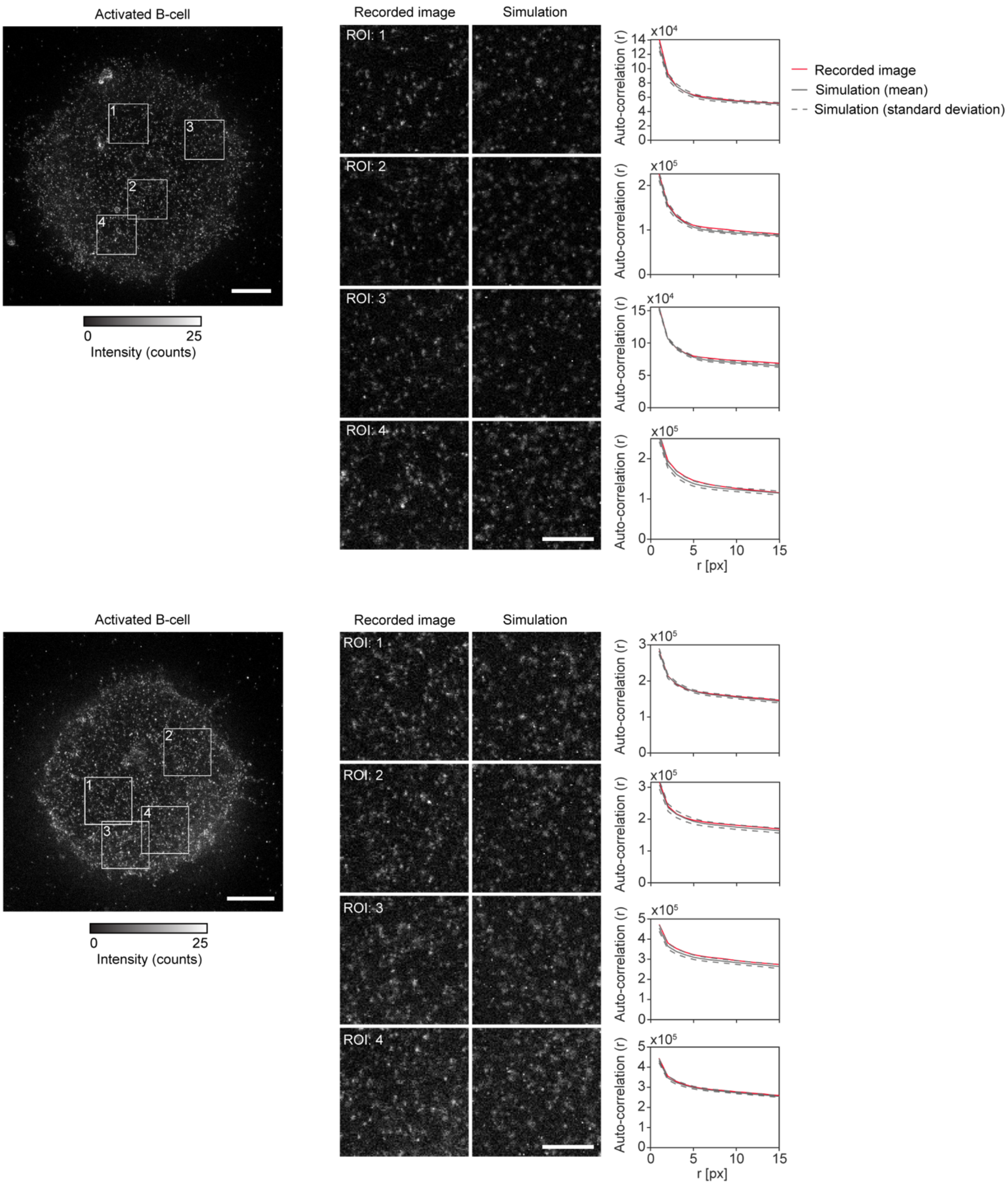

Appendix Figure S11 - STED microscopy of activated B-cells confirmed a randomized distribution of pMHCIIs.

STED microscopy of activated B-cells labeled with saturated amounts of 14.4.4 scF_V-abSTAR635P. Images represent STED recordings of cells and simulated STED images of randomly distributed fluorescence molecules with the number of molecules per area and the point-spread function matched to the experimental data. Right panels show the image autocorrelation analysis of each recorded STED image (100 x 100 px ROI, blue), as a function of the distance *r* (1 pixel (px) = 25 nm); gray line and gray dotted line indicate the ACF of a reference (mean and standard deviation, respectively), for a random distribution derived from simulations based on the experimentally determined properties of the point-spread function of n = 5 independent simulations. Scale bars: 2.5 µm (overview) and 1 µm (enlarged ROI).

Appendix Figure S12.

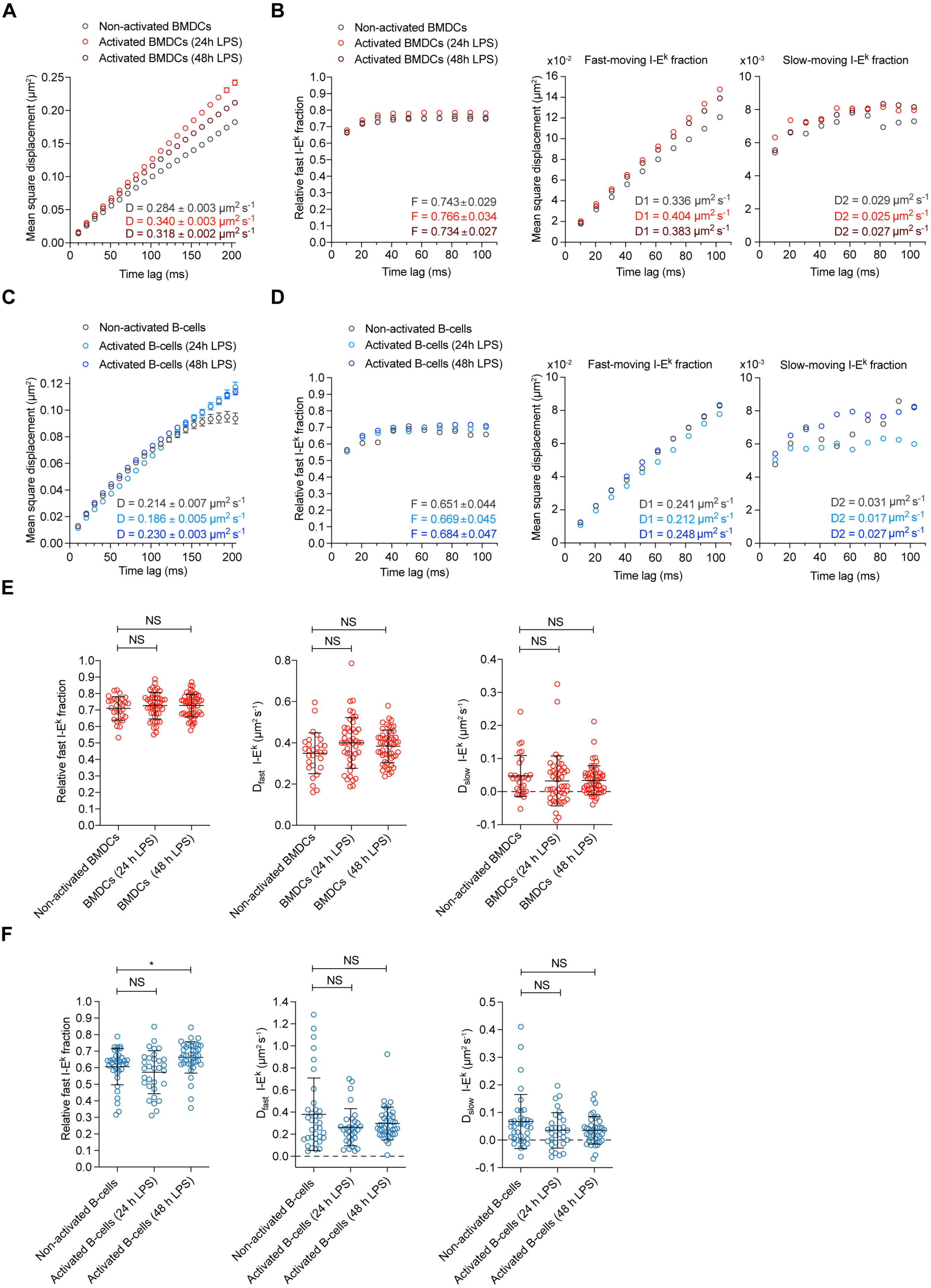

Appendix Figure S12 - Diffusion analysis of I-E^k on non-activated and activated BMDCs and B-cells at 37 °C.

A Mean square displacement plot of recorded I-E^k trajectories on non-activated and activated (24 h LPS or 48 h LPS) BMDCs. Statistics: mean and standard deviation; number of trajectories n = 14,001 (29 non-activated BMDCs); n = 20,497 (45 activated BMDCs, 24 h LPS); n = 29,731 (54 activated BMDCs, 48 h LPS). D = diffusion constant.

B Left panel: A binary fit of the data in (A) revealed the relative fraction of fast- and slow-moving I-E^k molecules on non-activated and activated BMDCs. Middle panel: Mean square displacement plot of fast-moving I-E^k molecules. Right panel: Mean square displacement plot of slow-moving I-E^k molecules. Statistics: mean and standard deviation. F = fraction of fast-moving molecules. D1 = diffusion constant of fast-moving fraction. D2 = diffusion constant of slow-moving fraction. Diffusion constants were calculated by fitting through the first two data points. Fraction was calculated by fitting through the first ten data points.

C Mean square displacement plot of recorded I-E^k trajectories on non-activated and activated (24 h LPS or 48 h LPS) B-cells. Statistics: mean and standard deviation; number of trajectories n = 3,832 (44 non-activated B-cells); n = 4,555 (33 activated B-cells, 24 h LPS); n = 14,650 (42 activated B-cells, 48 h LPS). D = diffusion constant.

D Left panel: A binary fit of the data in (C) revealed the relative fraction of fast- and slow-moving I-E^k molecules on non-activated and activated B-cells. Middle panel: Mean square displacement plot of fast-moving I-E^k molecules. Right panel: Mean square displacement plot of slow-moving I-E^k molecules. Statistics: mean and standard deviation. F = fraction of fast-moving molecules. D1 = diffusion constant of fast-moving fraction. D2 = diffusion constant of slow-moving fraction. Diffusion constants were calculated by fitting through the first two data points. Fraction was calculated by fitting through the first ten data points.

E, F Quantification of the binary diffusion data from (A) and (C) on individual non-activated and activated BMDCs (E) and B-cells (F). Each circle represents the mean of all trajectories of a single cell after being categorized by the binary fit. Statistics: mean and standard deviation; unpaired two-tailed student's t-test. P value format: $P > 0.05$ (NS), $P \leq 0.05$ (*).

Appendix Figure S13.

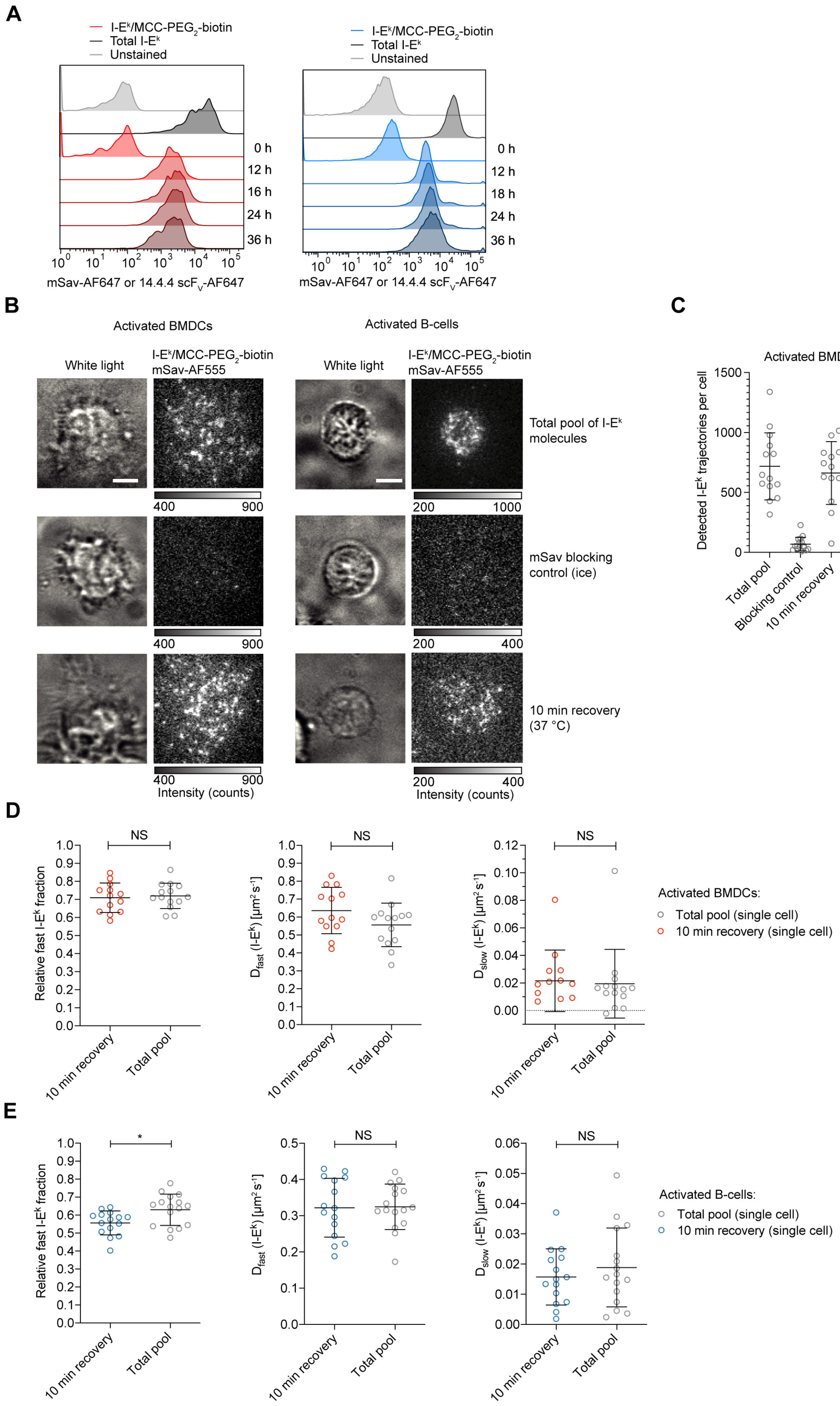

Appendix Figure S13 - Flow cytometry and diffusion analysis of newly-arriving I-E^k in complex with MCC-PEG$_2$-biotin on activated BMDCs and B-cells.

A Histograms derived from the flow cytometry analysis (Fig 4B) of activated BMDCs (n > 1,000 cells, left) and B-cells (n > 15,000 cells, right), which were pulsed with MCC-PEG$_2$-biotin for up to 36 h and stained with 14.4.4 scF$_V$-AF647 (all surface resident I-E^k molecules) or mSav-AF647 (only I-E^k/MCC-PEG$_2$-biotin).

B Activated BMDCs (left images) and B-cells (right images) were incubated with 50 μM MCC-PEG$_2$-biotin for twelve hours (BMDCs) and six hours (B-cells), washed and either stained with single molecule dilutions of mSav-AF555 to visualize a subset of surface-resident I-E^k/MCC-PEG$_2$-biotin molecules or treated with unlabeled monovalent streptavidin to block all surface-resident I-E^k/MCC-PEG$_2$-biotin molecules. Next, APCs treated with unlabeled mSav were rested for 10 minutes at 37°C to allow newly synthesized pMHCIIs to reach the cell surface. The latter was stained with mSav-AF555 immediately after to visualize newly surface-exposed I-E^k/MCC-PEG$_2$-biotin molecules. Cells were kept on ice after blocking with unlabeled mSav to serve as blocking control. Under these conditions, most of the newly synthesized I-E^k molecules did not appear on the cell surface and hence could not be stained with mSav-AF555. Scale bars, 5 μm.

C Number of recorded trajectories in the "total pool", "newly-arriving" and the "blocking control" fraction. 90% of recorded trajectories in the "newly-arriving" fraction appeared within the recovery time of ten minutes. Statistics: mean and standard deviation of n = 14 - 16 cells (total pool), n = 14 - 16 cells (blocking control) and n = 13 - 15 cells (10 min recovery) of one experiment. Each gray circle represents a cell.

D, E Quantification of the binary diffusion data (Fig 4D-E) of newly-arriving and a subset of all I-E^k molecules on individual activated BMDCs (D) and B-cells (E). Each circle represents the mean of all trajectories of a single cell after being categorized by the binary fit. Statistics: mean and standard deviation; unpaired two-tailed student's t-test. P value format: P > 0.05 (NS), P ≤ 0.05 (*).

Appendix Figure S14.

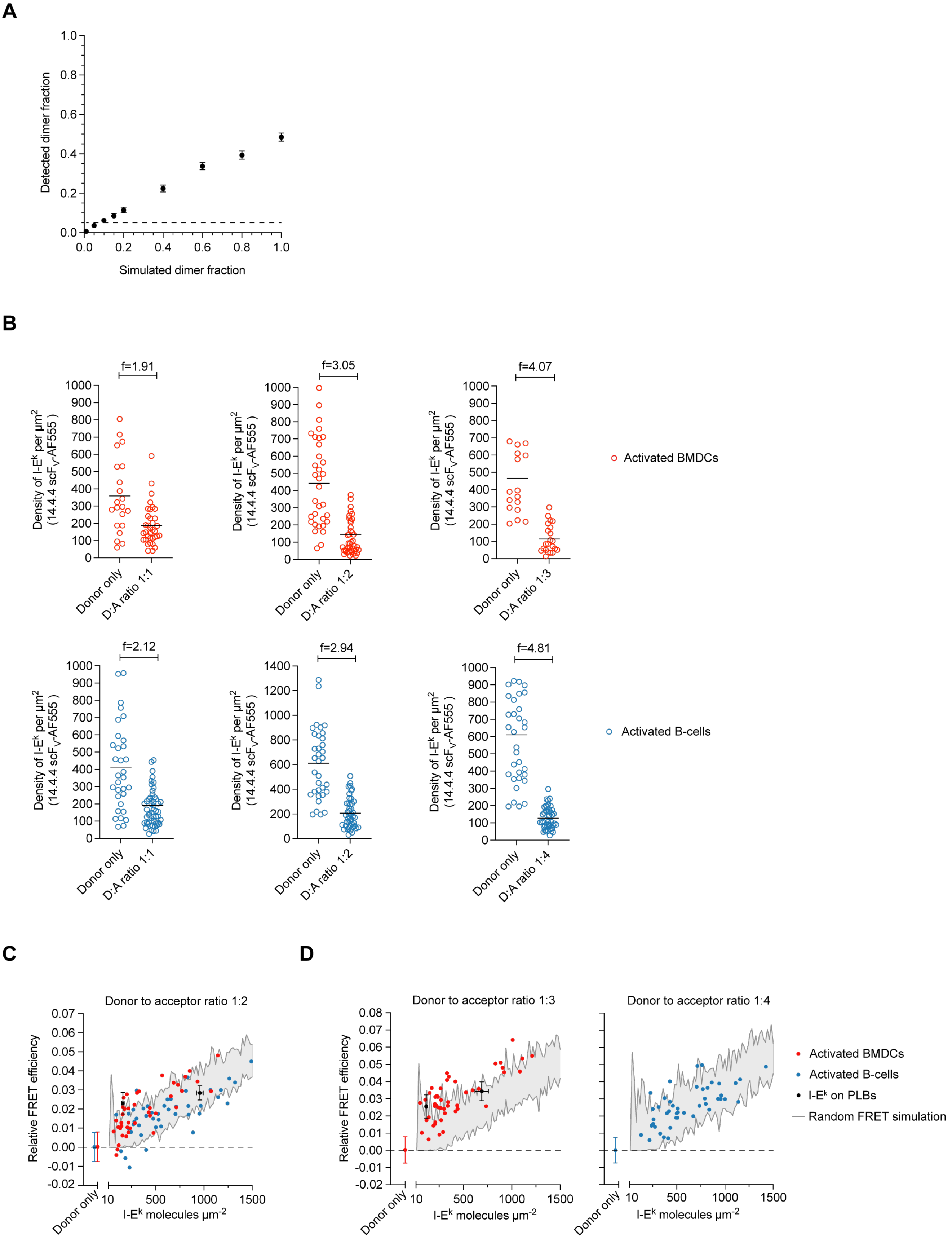

Appendix Figure S14 - TOCCSL simulation and quantitation of I-E^k surface densities and FRET measurements of I-E^k-bound 14.4.4 scF$_V$ on BMDCs, B-cells and PLBs.

A Monte-Carlo based simulations of TOCCSL experiments show a ~ 50 % reduction of detected dimer fractions due to the presence of partially photobleached dimers. Shown are the mean apparent/detected dimer fractions and the error bar (95 % bootstrapping confidence interval) from 1000 independent simulations. Dashed line shows the 5 % detection level of the TOCCSL method.

B Estimation of the total I-E^k density on BMDCs (upper row, red) and B-cells (lower row, blue) after adjusting the quantities of the 14.4.4 scF$_V$-AF555 (FRET donor, D) and the 14.4.4 scF$_V$-AF647 (FRET acceptor, A) to give rise to 1:1 (first column), 1:2 (second column) and 1:3 or 1:4 (third column) molecular ratios. Total I-E^k surface densities were measured using the 14.4.4 scF$_V$-AF555 as molecular probe. The reduction of the number of FRET donor molecules upon pre-mixing with the FRET acceptor was used to calculate the actual ratio r of the donor and acceptor molecules on the cells (e.g. donor only r = 1; 1:1 premix r = 2, 1:2 premix r = 3, 1:3 premix r = 4, 1:4 premix r = 5). The actual ratio (e.g. D:A=1:1, r = 1.91) was used to calculate the total density of donor and acceptor molecules from the measured density of donor molecules (14.4.4 scF$_V$-AF555) after the DRAAP sequence. Each panel represents an individual experiment with n = 17 – 43 activated BMDCs and n = 32 – 54 activated B-cells. Each circle represents a cell.

C FRET measurements of activated B-cells and BMDCs stained with a 1:2 premix of the 14.4.4 scF$_V$-AF555 and 14.4.4 scF$_V$-AF647. PLBs were decorated with unlabeled I-E^k/MCC followed by the 14.4.4 scF$_V$-AF555 and the 14.4.4 scF$_V$-AF647 in a 1:2 premix at the indicated total densities. Density dependent FRET efficiencies estimated from randomized molecular pMHCII encounters through Monte Carlo simulations are shown in gray for a 1:2 donor to acceptor ratio. These data represent one experiment. Each dot represents a single cell measurement. Error bars represent mean and standard deviation of n = 71 BMDCs, n = 131 B-cells (donor only) and n = 30 PLB positions. Upper and lower boundaries of the gray area represent range for n = 20 simulations per data point.

D FRET measurements of activated B-cells and BMDCs performed as in (C), though stained with a 1:3 or 1:4 premix of the 14.4.4 scF_V-AF555 and 14.4.4 scF_V-AF647. Density dependent FRET efficiencies estimated from randomized molecular pMHCII encounters through Monte Carlo simulations are shown in gray for a 1:3 and 1:4 donor to acceptor ratio. Each dot represents a single cell measurement. Error bars represent mean and standard deviation of n = 71 BMDCs, n = 131 B-cells (donor only) and n = 30 PLB positions. Data were pooled from two (BMDCs) and one (B-cells) independent experiments. Upper and lower boundaries of the gray area represent range for n = 20 simulations per data point.

Appendix Figure S15.

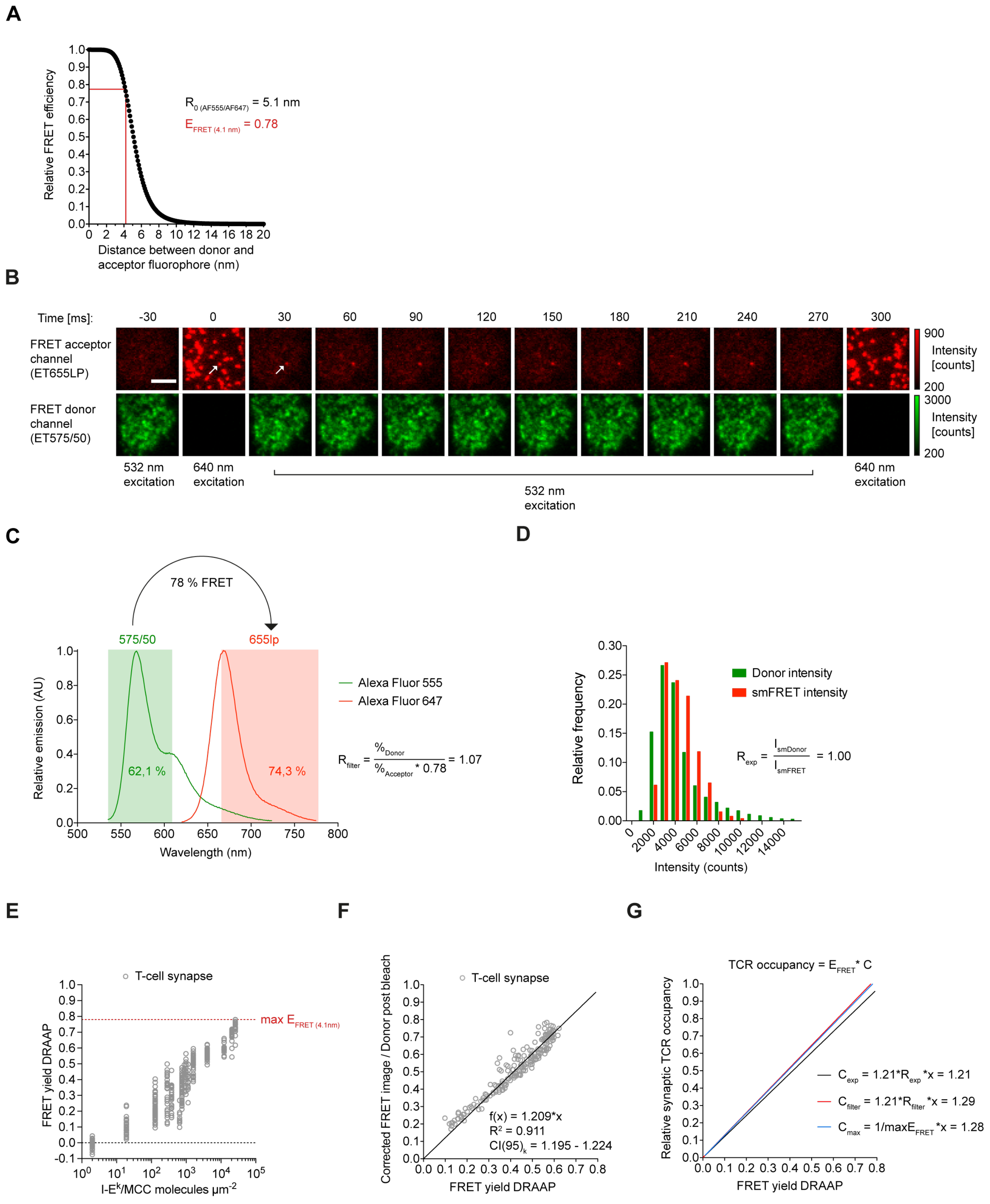

Appendix Figure S15 - Quantitation of synaptic TCR occupancies (the ratio of pMHCII-bound TCRs to total TCRs) via FRET imaging.

A Considering an inter-dye distance of approximately 4.1 nm within the TCR-pMHCII complex (see Fig 6A) and a Förster radius R_0 of 5.1 nm for the employed AF555:AF647 FRET dye pair, TCR binding of single pMHCII was calculated to result in a theoretical FRET yield of about 78%.

B Example of a single molecule FRET experiment. A series of images was acquired with the support of an emission beam splitter giving rise to a green channel (FRET donor channel, filter: ET575/50) and a red channel (FRET acceptor channel, filter: ET655LP). Single molecule FRET events (as indication for single TCR-pMHCII binding events) appear and disappear in single steps and align with single I-E^k/MCC-AF647 (acceptor) events (white arrow). Scale bar, 5 µm.

C Estimating the conversion factor $R_{filter} = \%_{Donor} / \%_{Acceptor}\ x\ maxE_{FRET}$ based on the emission filters ET575/50 (FRET donor channel) and ET655LP (FRET acceptor channel, left). Percentages indicate the fraction of transmitted light (including bleed through and cross excitation) upon 532 nm laser excitation and 100% FRET efficiency. Estimation of R_{filter} is based on the theoretical maximal FRET yield ($maxE_{FRET} = 0.78$) of the applied FRET system (AF555/AF647 FRET pair, $R_0 = 5.1$ nm, $r = 4.1$ nm) and yielded in $R_{filter} = 1.07$.

D Calculating the conversion factor $R_{exp} = I_{smDonor}/I_{smFRET}$ (I = intensity) from the experimentally derived mean intensities of n = 17,501 single molecule donor events in the green channel (TCRβ chain, H57 scF$_V$-AF555) and n = 262 single molecule FRET events in the red channel (TCRs binding to I-E^k/MCC-AF647, right). Histogram binning = 1,000. $R_{exp} = 1.00$.

E Quantification of synaptic FRET yields of T-cells approaching SLBs presenting increasing I-E^k/MCC-AF647 densities together with ICAM-1 and B7-1. Each circle represents the FRET yield of an entire T-cell synapse (n = 9 to 52 cells per condition) recorded between two and twelve minutes after the T-cell landed on the functionalized PLBs. This data was pooled from five biological replicates. The red line indicates the theoretical maximal FRET yield (max E_{FRET} = 0.78) in the T-cell synapse when all synaptic TCRs are bound to I-E^k/MCC-AF647.

F Correlation between FRET yields determined by FRET DRAAP and apparent FRET values (corrected FRET image divided by DD_{post}) of n = 203 T-cell synapses. The slope of the linear regression fit represents the conversion factor *C*. This data was pooled from three biological replicates.

G Comparison of the theoretically (C_{max}, C_{filter}) and experimentally (C_{exp}) determined conversion factors. The experimentally determined conversion factor C = 1.21 was used to calculate TCR occupancies from relative FRET yields by multiplying the relative FRET image (E_{FRET}) with 1.21.

Appendix Figure S16.

A

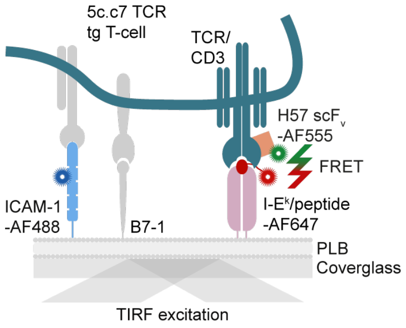

B

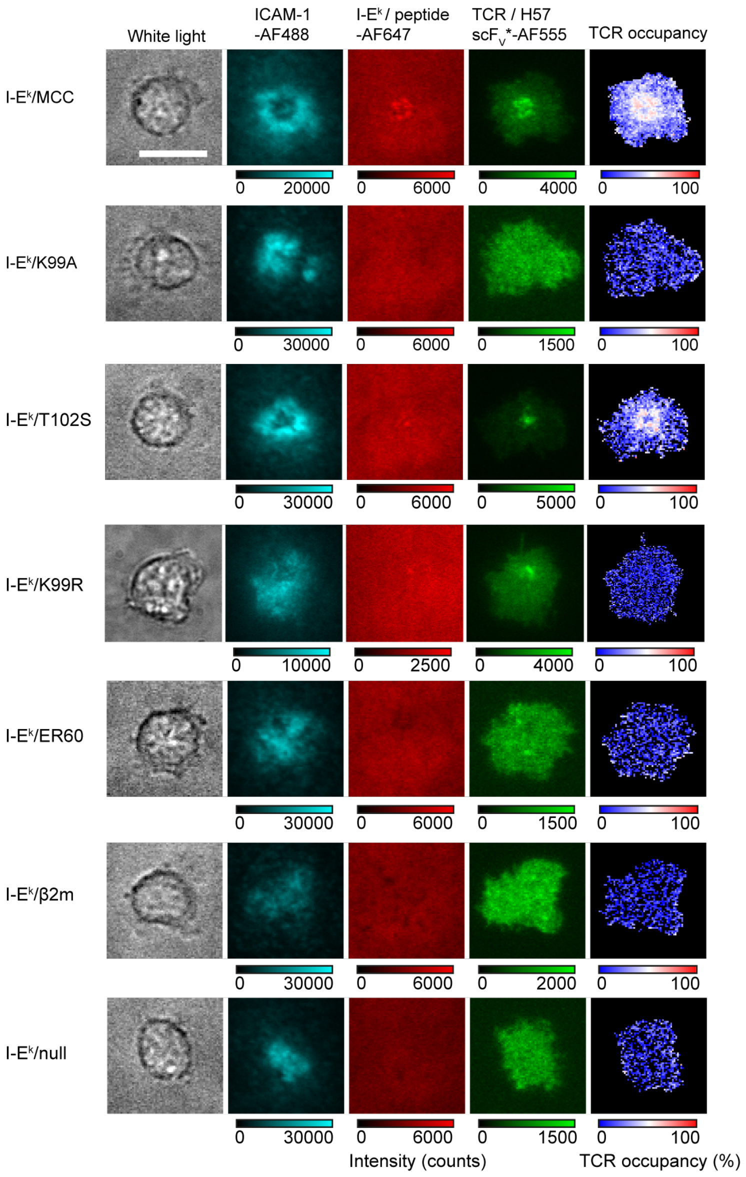

Appendix Figure S16 - Synaptic TCR-pMHCII binding and immune synapse formation as visualized via TIRF microscopy.

A Scheme of the FRET-based visualization and quantitation of synaptic TCR-pMHCII binding.

B 5c.c7 TCR-transgenic T-cells were stained via their TCRβ chain with the H57 scF_V-AF555 (green) and allowed to spread on a functionalized PLBs presenting I-E^k in complex with strong agonist (MCC), weak agonist (T102S), antagonist (K99R) potential co-agonists (K99A, ER60, β2m) and non-binder (null) peptide-AF647 derivatives (red) together with ICAM-1-AF488 (cyan) and B7-1 (unlabeled). T-cell synapses were recorded after 10 minutes. ICAM-1 and B7-1 were displayed at a density of ~ 100 molecules per μm^2. I-E^k was displayed at a density of ~1,000 molecules per μm^2. Synaptic TCR-pMHCII binding was quantitated using FRET DRAAP. Cyan, red or green color bars show intensity values (counts). The union jack color bar represents the TCR occupancy in percent. Scale bar, 10 µm.

Appendix Figure S17.

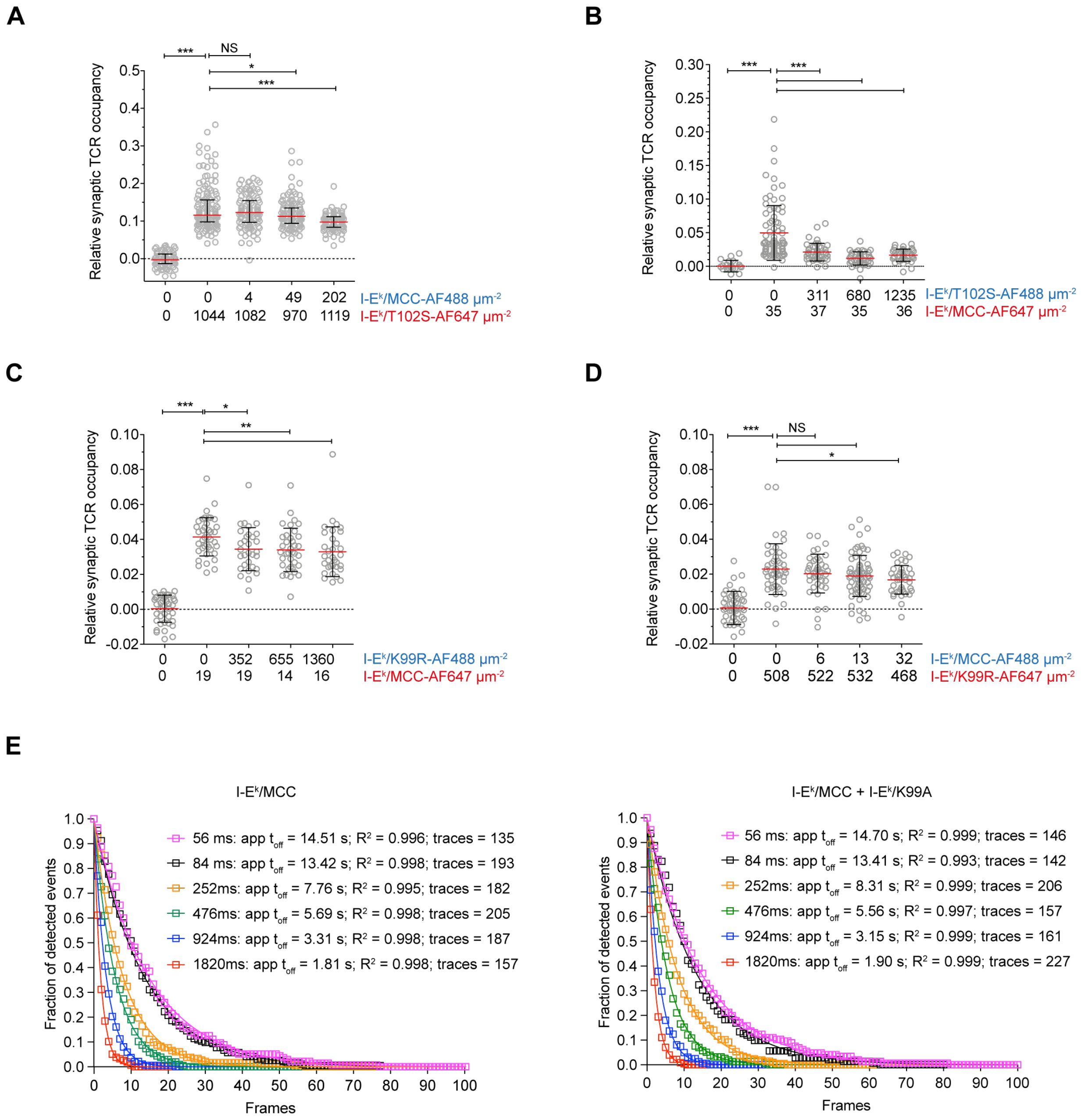

Appendix Figure S17 - Synaptic engagement and recognition of agonist I-E^k/MCC present in low abundance and co-presented "bystander" ligands present in high abundance and calculation of apparent lifetimes from TCR:I-E^k/MCC binding events recorded via smFRET at different time lags.

A - D Synaptic TCR occupancy of 5c.c7 T-cells approaching SLBs presenting ICAM-1 and B7-1 together with the agonist I-E^k/MCC-AF647 in low abundance and increasing densities of I-E^k/T102S-AF488 (B) or I-E^k/K99R-AF488 (C). The impact of co-presented I-E^k/T102S and I-E^k/K99R on the detection of the agonist I-E^k/MCC-AF647 was quantified by measuring the FRET yield between H57 scF$_V$-AF555 bound to the 5c.c7 TCRβ chain and I-E^k/MCC-AF647. To assay the influence of increasing densities of the agonist I-E^k/MCC-AF488 on the engagement of I-E^k/T102S-AF647 or I-E^k/K99R-AF647 in high abundance, we measured the FRET yield between H57 scF$_V$-AF555 in complex with the 5c.c7 TCRβ and either I-E^k/T102S-AF647 (A) or I-E^k/K99R-AF647 (D). Each circle represents a single T-cell synapse (n = 23 to 147 cells per condition) pooled from one experiment. Statistics: mean, standard deviation and unpaired two-tailed student's t test. P value format: $P > 0.05$ (NS), $P \leq 0.05$ (*), $P \leq 0.01$ (**), $P \leq 0.001$ (***).

E The inverse cumulative decay function of the smFRET traces was fitted to a one-exponential decay function (start 1.0, plateau 0.0) to extract the apparent lifetime (app $t_{off} = 1/k_{off}$) for each time lag (56, 84, 252, 476, 924, 1,820 ms). FRET traces longer than 100 frames were discarded. smFRET measurements were performed at 26 °C. Data were derived from one experiment with n = 10 - 20 cells per time lag.

Appendix Figure S18.

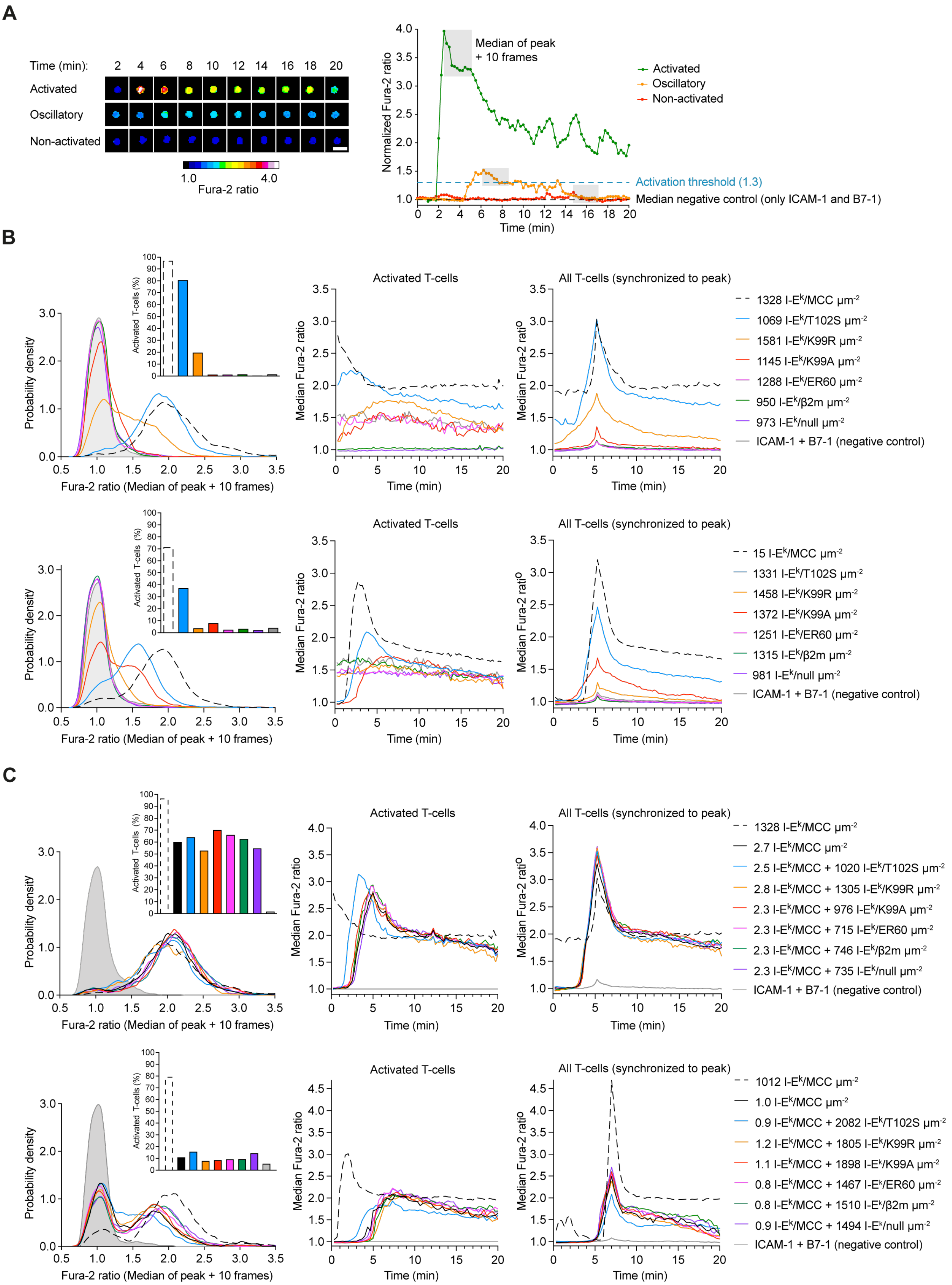

Appendix Figure S18 - Highly abundant bystander pMHCIIs did not sensitize T-cells for low levels of antigenic pMHCIIs.

A Example of a Fura-2-labeled T-cells approaching a functionalized PLB. T-cells react within seconds to agonist I-E^k/MCC with an increase of the Fura-2 ratio (i.e. ratiometric image after 340 nm and 380 nm excitation) as the result of a calcium signaling response. T-cells did not become activated in the absence of the stimulatory ligand (only ICAM-1 and B7-1 present). When confronted with low doses of antigen, T-cells showed an oscillatory Fura-2 ratio behavior, with changes in Fura-2 ratio amounting to lower numbers than observed in cases of full activation. All T-cell tracks were normalized to the median Fura-2 ratio of the negative control (ICAM-1 and B7-1at 100 molecules per μm^2). T-cells which stayed 80% of their recorded track time above the Fura-2 ratio of 1.3 were counted as "activated". T-cells, which stayed 80% of their track time below this threshold were counted as "non-activated". All others were considered "oscillatory". We chose an activation threshold of 1.3 for all experiments based on a receiver operating curve (ROC curve) between a positive (> 10 I-Ek/MCC μm^{-2}, ICAM-1 and B7-1 at 100 μm^{-2}) and negative control (ICAM-1 and B7-1 at 100 μm^{-2}). For more detailed information refer to the Material and Methods section. Scale bar, 20 μm.

B Calcium imaging of T-cells exposed to PLBs displaying ICAM-1 and B7-1 (at 100 molecules per μm^2) together with I-E^k in complex with the MCC, T102S, K99R, K99A, ER60, β2m or null peptides. Shown are percentage of activated T-cells (left box), probability density plot of Fura-2 ratios of individual T-cells (left), mean Fura-2 ratio of activated T-cells over time (middle), and mean Fura-2 ratio of the entire T-cell population synchronized with regard to the highest Fura-2 value of the track (right). Two biological replicates are shown (upper and lower panel). Upper panel: n = 486 – 1,268 cells per condition. Lower panel: n = 327 - 552 cells per condition.

C Calcium response of T-cells contacting PLBs displaying the agonist I-E^k/MCC in low abundance and co-presented with bystander pMHCIIs at high densities as well as ICAM-1 and B7-1 (at 100 molecules per μm^2). Shown are percentage of activated T-cells (left box), probability density plot of Fura-2 ratios of individual T-cells (left), mean Fura-2 ratio of

activated T-cells over time (middle), and mean Fura-2 ratio of the entire T-cell population synchronized to the highest Fura-2 value of the track (right). Two biological replicates are shown (upper and lower panel). Upper panel: n = 153 - 415 cells per condition. Lower panel: n = 136 - 625 cells per condition.

Appendix Figure S19.

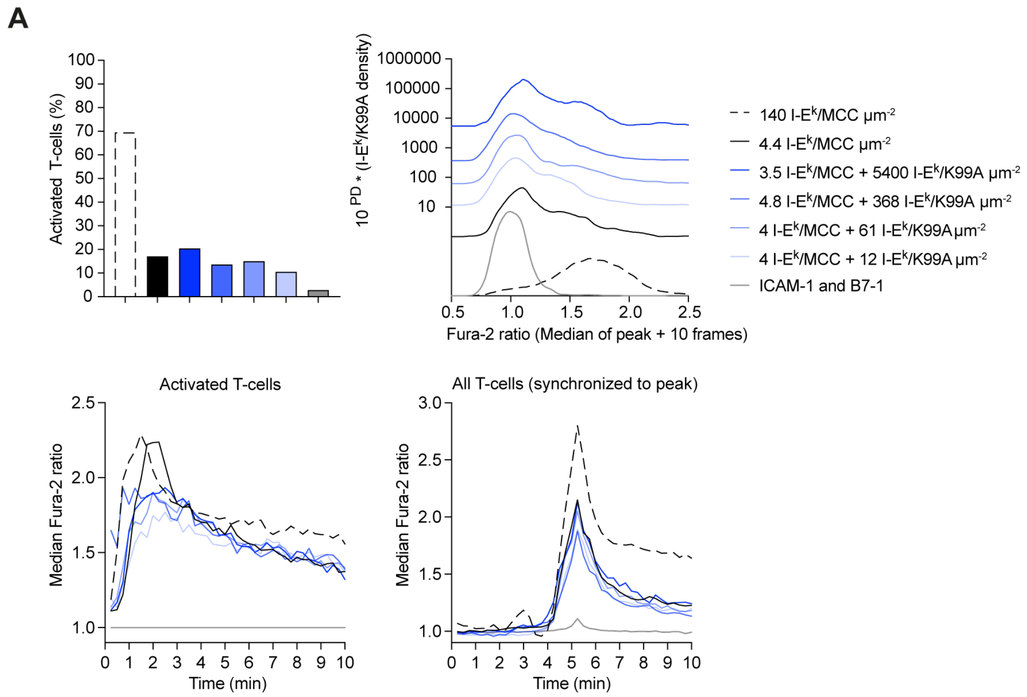

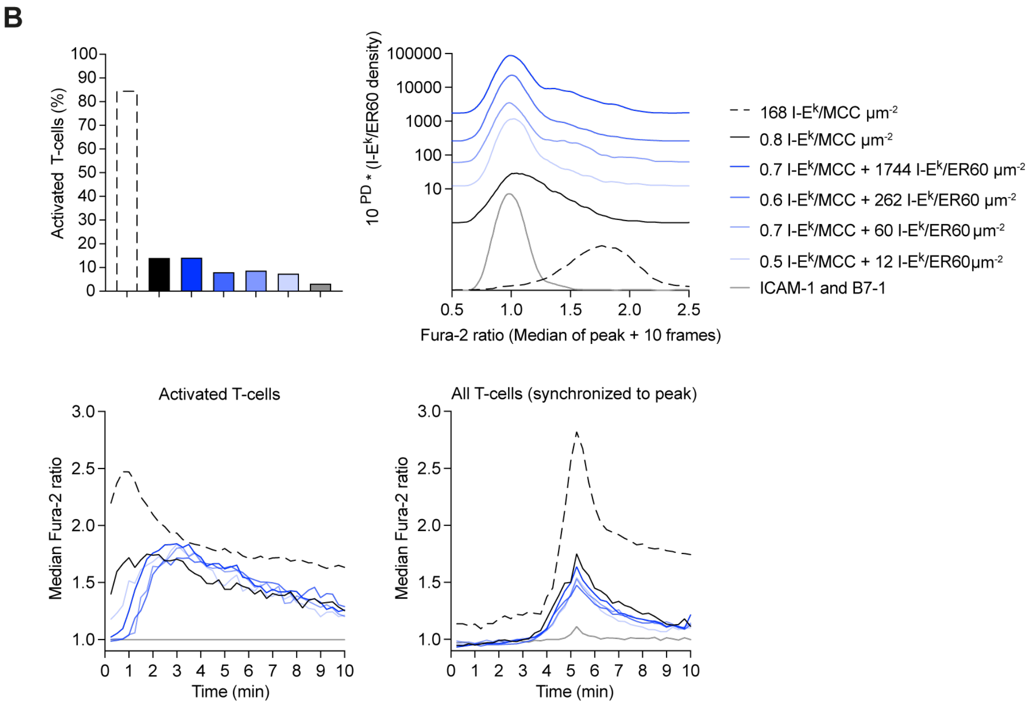

Appendix Figure S19 - Co-agonist candidates I-E^k/K99A and I-E^k/ER60 supplied in increasing densities did not sensitize T-cells for the detection of agonist I-E^k/MCC.

A, B Calcium response of T-cells in contact with PLBs displaying the agonist I-E^k/MCC in low abundance together with the co-agonist candidate I-E^k/K99A (A) or I-E^k/ER60 (B) at supraphysiological densities and ICAM-1 and B7-1 at 100 molecules per µm^2. Shown are percentages of activated T-cells (upper left), probability density plot of Fura-2 ratios of individual T-cells (upper right), mean Fura-2 ratio of activated T-cells over time (lower left), and mean Fura-2 ratio of the entire T-cell population synchronized to the highest Fura-2 value of the track (lower right). n = 186 - 590 cells per condition (A). n = 156 - 411 cells per condition (B).

Appendix Figure S20.

A

```
            10         20         30         40         50         60         70
1   MQVQLQQSGP DLVKPGASVT ISCKASGYAF SSSWMSWLKQ RPGKGLEWIG WIFPRDGDTN YNGKFKGKAT
            80         90        100        110        120        130        140
71  LTADKSSSTA YMQLSSLTSE DSAVYFCARR GDYHYGMDYW GQGTSVTVSS AGGGGSGGGG SGGGGSDIVL
           150        160        170        180        190        200        210
141 TQSPASLAVS LGQRATISCR ASKSVSTSGY SYMHWYQQKP GQPPKLLIYL TSNLESGVPA RFSGSGSGTD
           220        230        240        249
211 FTLNIHPVEE EDAATYYCQH SRELPWTFGG GTKLEIKGS*--
            259      267
250 *GLNDIFEAQK IEWHEGSG--
```

B

```
            10         20         30         40         50         60         70
1   CATATGCAGG TTCAGCTGCA GCAGTCTGGT CCGGACCTGG TTAAACCGGG TGCGTCTGTT ACCATCTCTT
            80         90        100        110        120        130        140
71  GCAAAGCGTC TGGTTACGCG TTCTCTTCTT CTTGGATGTC TTGGCTGAAA CAGCGTCCGG GTAAAGGTCT
           150        160        170        180        190        200        210
141 GGAATGGATC GGTTGGATCT TCCCGCGTGA CGGTGACACC AACTACAACG GTAAATTCAA AGGTAAAGCG
           220        230        240        250        260        270        280
211 ACCCTGACCG CGGACAAATC TTCTTCTACC GCGTACATGC AGCTGTCTTC TCTGACCTCT GAAGACTCTG
           290        300        310        320        330        340        350
281 CGGTTTACTT CTGCGCGCGT CGTGGTGACT ACCACTACGG CATGGACTAC TGGGGTCAGG GTACCTCTGT
           360        370        380        390        400        410        420
351 TACCGTTTCT TCTGCGGGTG GCGGCGGTTC GGGTGGCGGT GGCTCTGGTG GCGGCGGTTC TGACATCGTT
           430        440        450        460        470        480        490
421 CTGACCCAGT CTCCGGCGTC TCTGGCGGTT TCTCTGGGTC AGCGTGCGAC CATCTCTTGC CGTGCGTCTA
           500        510        520        530        540        550        560
491 AATCTGTTTC TACCTCTGGT TACTCTTACA TGCACTGGTA CCAGCAGAAA CCGGGTCAGC CGCCGAAACT
           570        580        590        600        610        620        630
561 GCTGATCTAC CTGACCTCTA ACCTGGAATC TGGTGTTCCG GCGCGTTTCT CTGGTTCTGG TTCTGGTACC
           640        650        660        670        680        690        700
631 GACTTCACCC TGAACATCCA CCCGGTTGAA GAAGAAGACG CGGCGACCTA CTACTGCCAG CACTCTCGTG
           710        720        730        740        750     760/814  762/816
701 AACTGCCGTG GACCTTCGGT GGTGGTACCA AACTGGAAAT CAAAGGATCC *TAATAAAAGC TT
            760        770        780        790        800   804
751 *GGCCTGAACG ATATTTTTGA AGCGCAGAAA ATTGAATGGC ATGAAGGTAG CGGC
```

Appendix Figure S20 - DNA and protein sequence of the 14.4.4 scF$_V$.

A Protein sequence of the 14.4.4 scF$_V$. The variable domain of the heavy chain (V_H) (red) is connected via a linker sequence (green) to the variable domain of the light chain (V_L) (blue). The serine residue at position 120 which had been substituted for a cysteine residue to generate a 14.4.4 scF$_V$ suitable for site-specific modifications is underlined. The BirA ligase recognition sequence is indicated in violet (insertion site marked with asterisks).

B DNA sequence of the 14.4.4 scF$_V$ construct. The sequence of the heavy chain (V_H) (red) was connected via a linker sequence (green) to the light chain (V_L) (blue). The S120C conversion is underlined (T<u>C</u>T > T<u>G</u>T). The construct was cloned into the pEt21a(+) expression vector with the 5´restriction enzyme NdeI and the 3´restriction enzyme HindIII (highlighted in bold). To arrive at the 14.4.4 scF$_V$-biotin construct we inserted the BirA ligase recognition sequence (violet) upstream of the HindIII site and the two stop codons (marked with asterisks).

Appendix Table S1.

A

Layout	Ligand 1 (mSav-V)	Ligand 2 (mSav-X)	No ligand (%)	Single occupancy - ligand 1 (%)	Single occupancy - ligand 2 (%)	Double occupancy (%)
**M**	I-E^k/MCC	---	39.51 ± 1.38	60.49 ± 1.38	---	---
**D10**	I-E^k/MCC	---	10.12 ± 1.23	43.39 ± 5.28	---	46.50 ± 2.65
**D10**	I-E^k/K99A	---	10.12 ± 1.23	43.39 ± 5.28	---	46.50 ± 2.65
**D10**	I-E^k/MCC	I-E^k/K99A	9.87 ± 1.24	20.81 ± 1.85	22.31 ± 2.12	47.01 ± 2.73
**D20**	I-E^k/MCC	I-E^k/K99A	9.32 ± 0.73	24.35 ± 1.68	18.35 ± 0.87	47.98 ± 1.82

B

Layout	Ligand 1	Ligand 2	n	n_{low}	n_{high}	A_{max} (%)	$A_{max,low}$ (%)	$A_{max,high}$ (%)	T_A (μm^{-2})	$T_{A,low}$ (μm^{-2})	$T_{A,high}$ (μm^{-2})	# cells	# mice
**mSAv**	I-E^k/MCC	---	1.85	0.98	2.72	93.58	79.40	107.75	0.33	0.25	0.45	166 ± 45	4
**M**	I-E^k/MCC	---	4.00	0.30	7.70	93.15	85.05	101.25	0.40	0.33	0.49	186 ± 56	3
**D10**	I-E^k/MCC	---	4.00	1.95	6.05	91.61	86.90	96.32	0.48	0.43	0.55	150 ± 35	3
**D10**	I-E^k/MCC	I-E^k/K99A	3.27	1.83	4.71	87.91	82.47	93.35	0.34	0.28	0.42	123 ± 41	4
**D20**	I-E^k/K99A	I-E^k/K99A	2.14	1.26	3.02	90.91	82.40	99.43	0.46	0.32	0.64	134 ± 23	4
**D10**	I-E^k/K99A	---	n.a.	n.a.	n.a.	n.a.	n.a.	n.a.	n.a.	n.a.	n.a.	80 ± 25	3

C

Layout	mSAv- I-E^k/MCC	M- I-E^k/MCC	D10- I-E^k/MCC	D10- I-E^k/MCC + K99A	D20- I-E^k/MCC + K99A	D10- I-E^k/K99A
**mSAv**-I-E^k/MCC	-	n.s.	< 0.05	n.s.	n.s.	< 0.001
**M**-I-E^k/MCC	n.s.	-	n.s.	n.s.	n.s.	< 0.001
**D10**-I-E^k/MCC	< 0.05	n.s.	-	n.s.	n.s.	< 0.001
**D10**-I-E^k/MCC + K99A	n.s.	n.s.	n.s.	-	n.s.	< 0.001
**D20**-I-E^k/MCC + K99A	n.s.	n.s.	n.s.	n.s.	-	< 0.001
**D10**-I-E^k/K99A	< 0.001	< 0.001	< 0.001	< 0.001	< 0.001	-

Appendix Table S1 - Ligand occupancy of DNA origamis, fitting parameters of dose-response curves, and significance table of the indicated constructs.

A Ligand occupancy of DNA origami platforms. Efficiency of homo- or heterofunctionalization with biotinylated I-E^k/MCC-AF555 or I-E^k/K99A-AF647 was determined via sequential two-color colocalization experiments as described in Material and Methods. Data are the mean of at least two biological replicates (± SEM).

B Dose-response curves recorded for the indicated constructs were fitted with equation (12) to extract the activation threshold TA, the maximum response A_{max} and the Hill coefficient n. The mean number of cells per region (± SEM) and the number of mice to extract T-cells and generate dose-response curves are shown. The 95% confidence intervals are shown.

C Results of the significance test of activation thresholds for the indicated constructs. Statistical significance between the activation thresholds $T_{A,1}$ and $T_{A,2}$ for two different data sets was calculated via a bootstrap ratio test as follows: A bootstrap sample was obtained by drawing n data points (sampling with replacement) from a dose-response curve, where n equals the size of the data set. From each data set, 1000 bootstrap samples were drawn and fitted via $A = \frac{A_{max}}{1+10^{(logT_A - L)*n}}$. This yielded activation threshold values $T_{A,1}^i$ and $T_{A,2}^i$ $(i = 1, \ldots, 1000)$ for each of the bootstrap samples from the two different data sets. The ratio $T_{A,1}^i / T_{A,2}^i$ $(i = 1, \ldots, 1000)$ was calculated for each pair of bootstrap samples. If the 100*(1-α)% CI of log($T_{A,1}/T_{A,2}$) did not contain 0, the null hypothesis of equal T_A was rejected at a significance level of α.
