# Supplementary figures and images for: Monomeric agonist peptide/MHCII complexes activate T‐cells in an autonomous fashion
Source: EMBO Rep. 2023 Sep 28;24(11):e57842. doi: 10.15252/embr.202357842 (PMC10626418; doi:10.15252/embr.202357842)

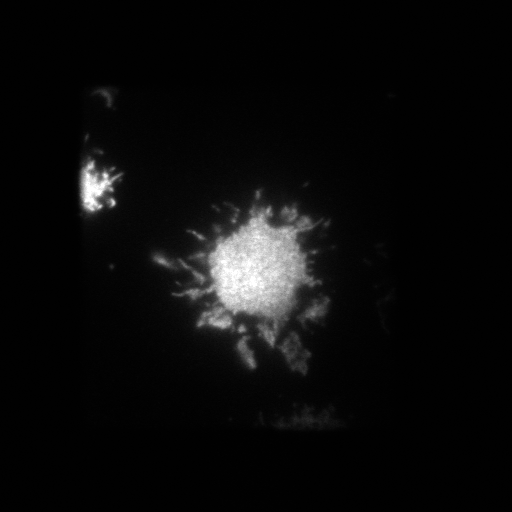

Supplement: Supplementary file 2 — Source Data for Figure 1 [file EMBR-24-e57842-s001.zip › Figure 1/E/Activated BMDC.tif]

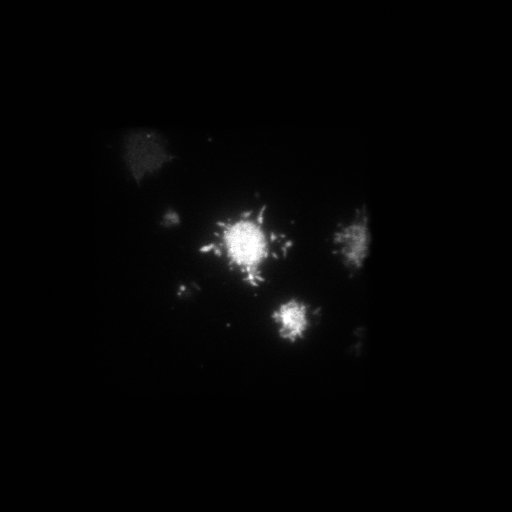

Supplement: Supplementary file 2 — Source Data for Figure 1 [file EMBR-24-e57842-s001.zip › Figure 1/E/Activated B-cell.tif]

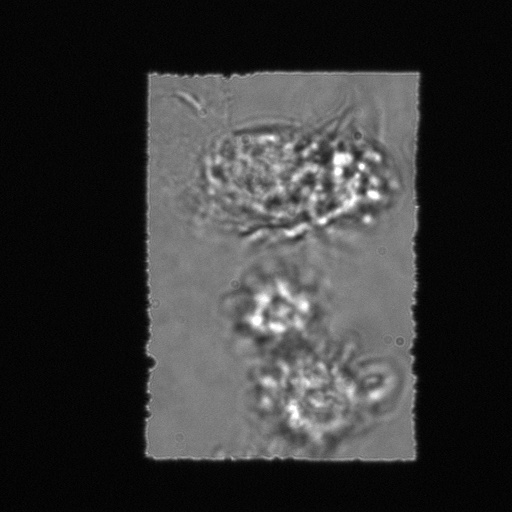

Supplement: Supplementary file 3 — Source Data for Figure 2 [file EMBR-24-e57842-s005.zip › Figure 2/C/Activated BMDC DEC205.tif]

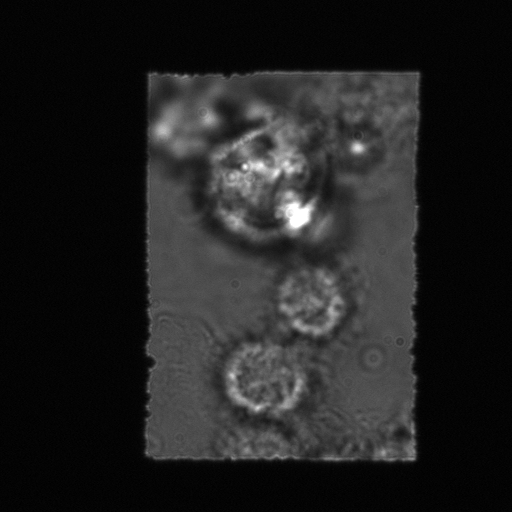

Supplement: Supplementary file 3 — Source Data for Figure 2 [file EMBR-24-e57842-s005.zip › Figure 2/D/Activated BMDC CD18.tif]

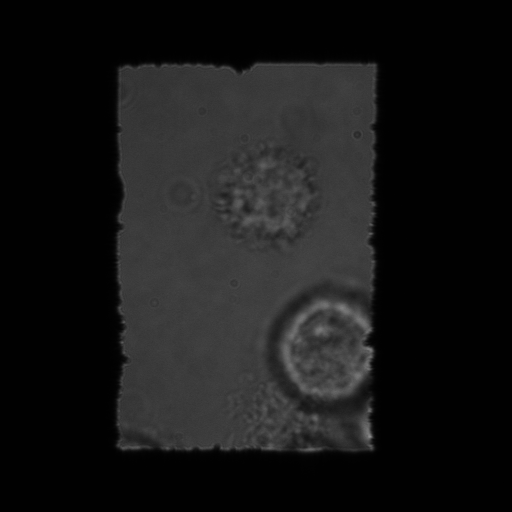

Supplement: Supplementary file 3 — Source Data for Figure 2 [file EMBR-24-e57842-s005.zip › Figure 2/B/Activated BMDC IEk.tif]

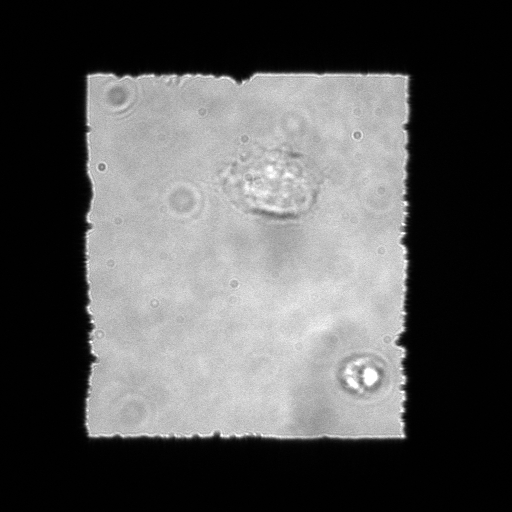

Supplement: Supplementary file 3 — Source Data for Figure 2 [file EMBR-24-e57842-s005.zip › Figure 2/B/Activated B-cell IEk.tif]

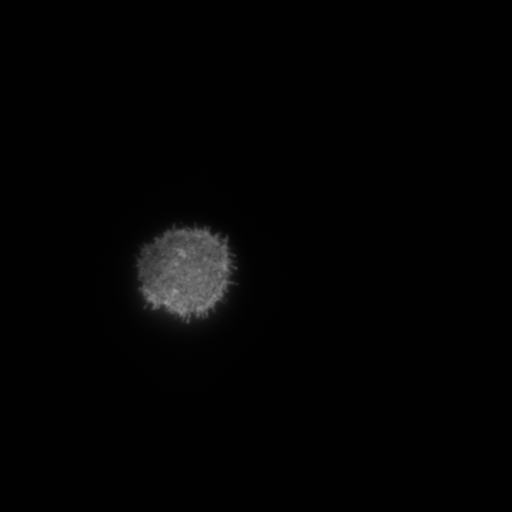

Supplement: Supplementary file 4 — Source Data for Figure 3 [file EMBR-24-e57842-s007.zip › Figure 3/H/Activated B-cell_full_activation.tif]

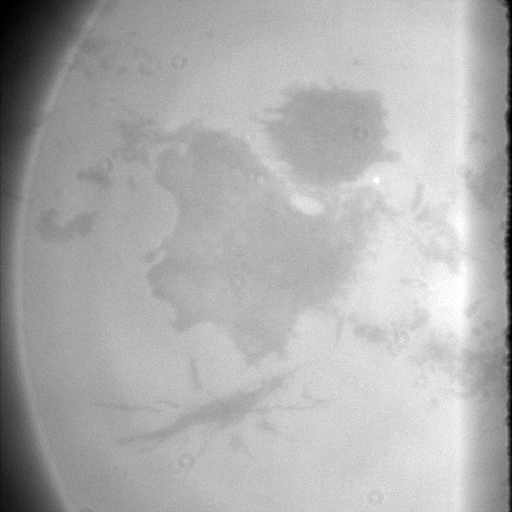

Supplement: Supplementary file 4 — Source Data for Figure 3 [file EMBR-24-e57842-s007.zip › Figure 3/H/Activated BMDC_IRM.tif]

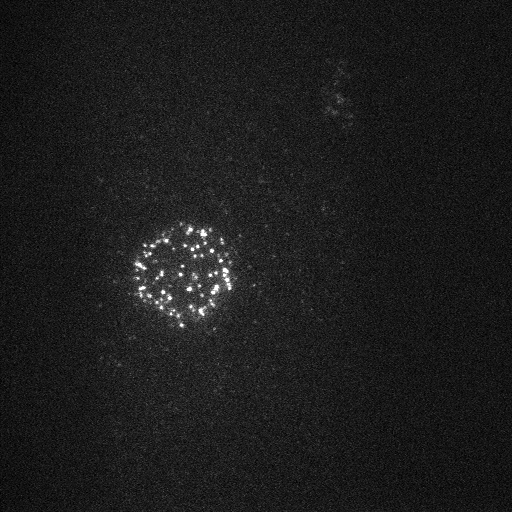

Supplement: Supplementary file 4 — Source Data for Figure 3 [file EMBR-24-e57842-s007.zip › Figure 3/H/Activated B-cell_time_series.tif]

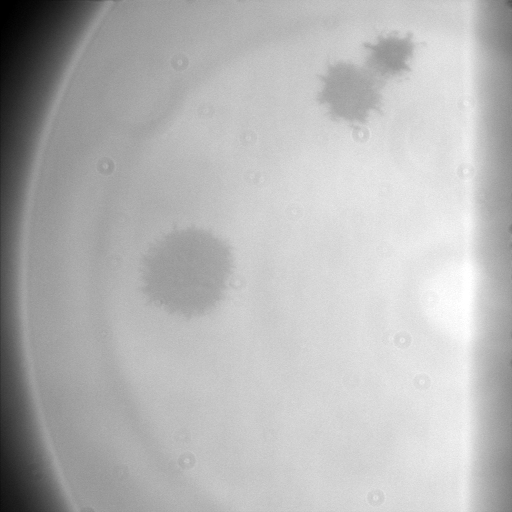

Supplement: Supplementary file 4 — Source Data for Figure 3 [file EMBR-24-e57842-s007.zip › Figure 3/H/Activated B-cell_IRM.tif]

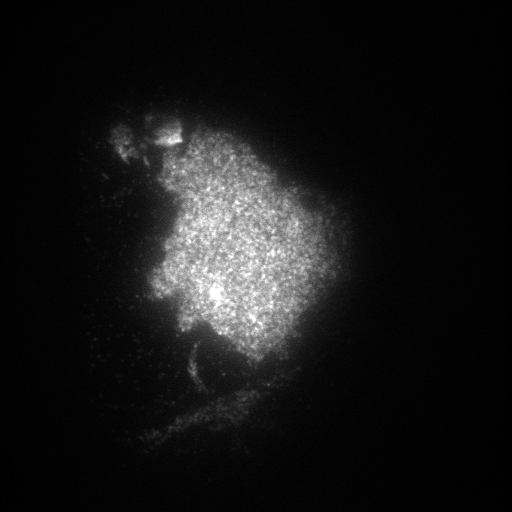

Supplement: Supplementary file 4 — Source Data for Figure 3 [file EMBR-24-e57842-s007.zip › Figure 3/H/Activated BMDC_full.tif]

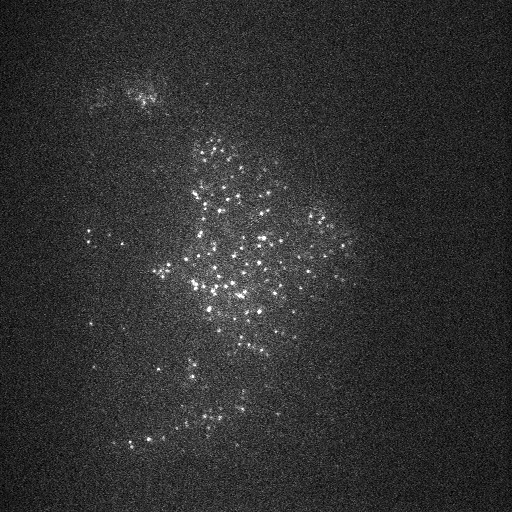

Supplement: Supplementary file 4 — Source Data for Figure 3 [file EMBR-24-e57842-s007.zip › Figure 3/H/Activated BMDC_time_series.tif]

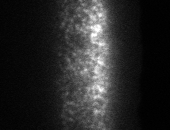

Supplement: Supplementary file 6 — Source Data for Figure 5 [file EMBR-24-e57842-s009.zip › Figure 5/A/Example TOCCSL sequence.tif]

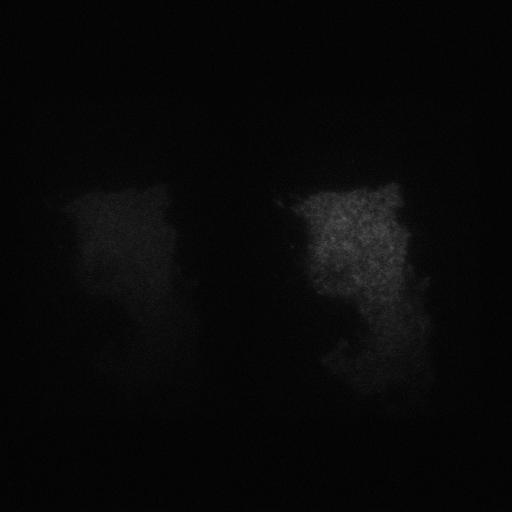

Supplement: Supplementary file 6 — Source Data for Figure 5 [file EMBR-24-e57842-s009.zip › Figure 5/F/100 nM mSav/green image before bleach.TIF]

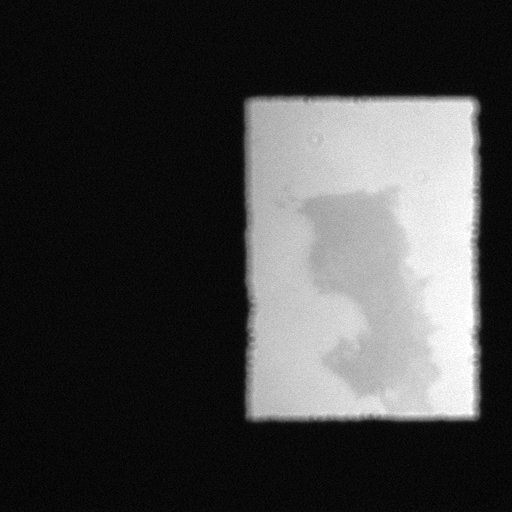

Supplement: Supplementary file 6 — Source Data for Figure 5 [file EMBR-24-e57842-s009.zip › Figure 5/F/100 nM mSav/IRM.tif]

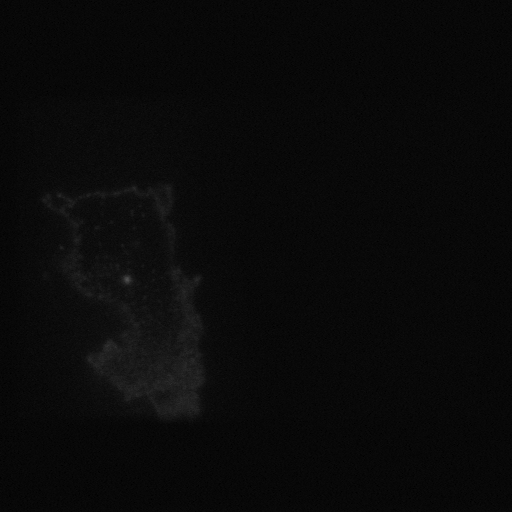

Supplement: Supplementary file 6 — Source Data for Figure 5 [file EMBR-24-e57842-s009.zip › Figure 5/F/100 nM mSav/red image after bleach.TIF]

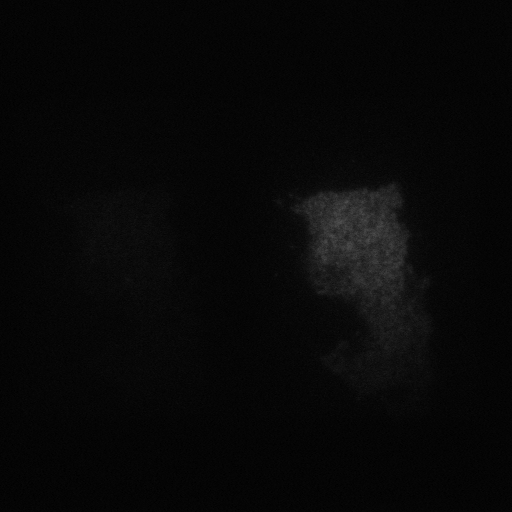

Supplement: Supplementary file 6 — Source Data for Figure 5 [file EMBR-24-e57842-s009.zip › Figure 5/F/100 nM mSav/green image after bleach.TIF]

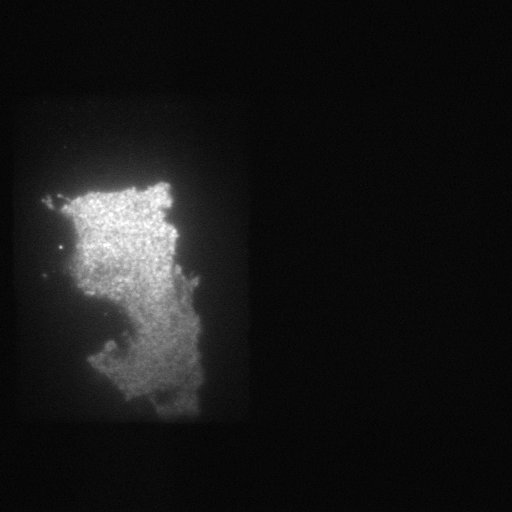

Supplement: Supplementary file 6 — Source Data for Figure 5 [file EMBR-24-e57842-s009.zip › Figure 5/F/100 nM mSav/red image before belach.TIF]

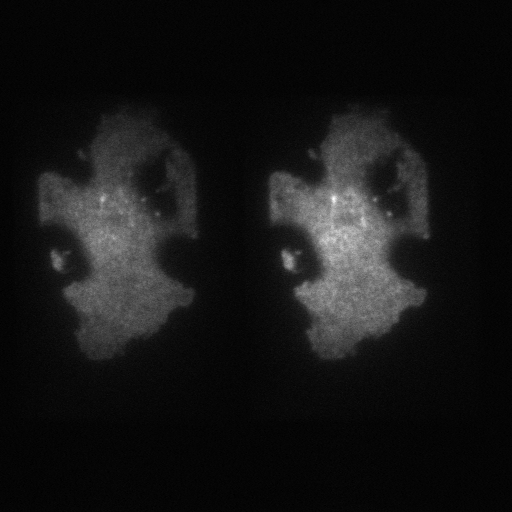

Supplement: Supplementary file 6 — Source Data for Figure 5 [file EMBR-24-e57842-s009.zip › Figure 5/F/118 nm diSav/green image before bleach.TIF]

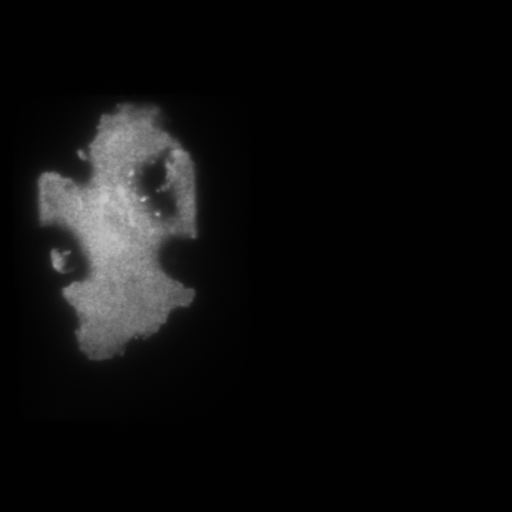

Supplement: Supplementary file 6 — Source Data for Figure 5 [file EMBR-24-e57842-s009.zip › Figure 5/F/118 nm diSav/red image before bleach.TIF]

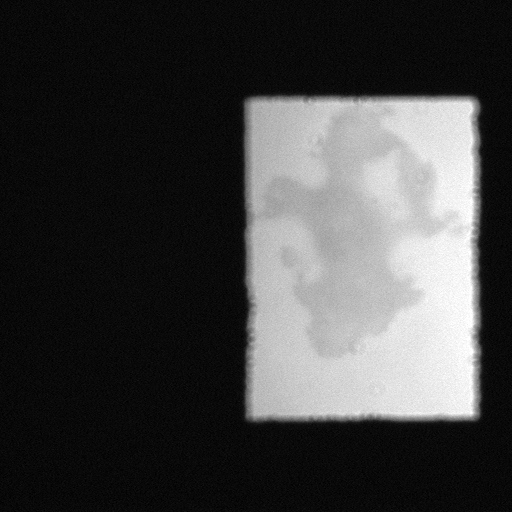

Supplement: Supplementary file 6 — Source Data for Figure 5 [file EMBR-24-e57842-s009.zip › Figure 5/F/118 nm diSav/IRM.tif]

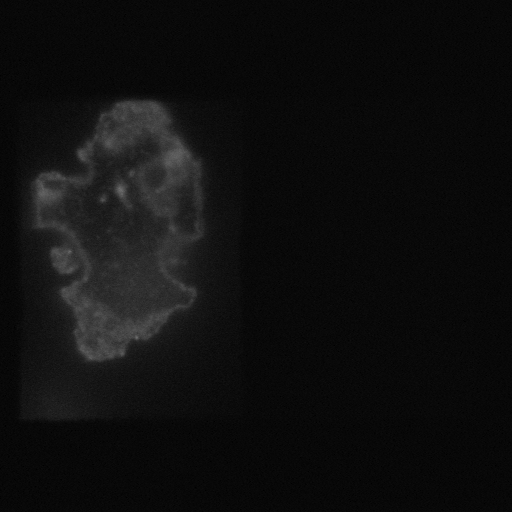

Supplement: Supplementary file 6 — Source Data for Figure 5 [file EMBR-24-e57842-s009.zip › Figure 5/F/118 nm diSav/red image after bleach.TIF]

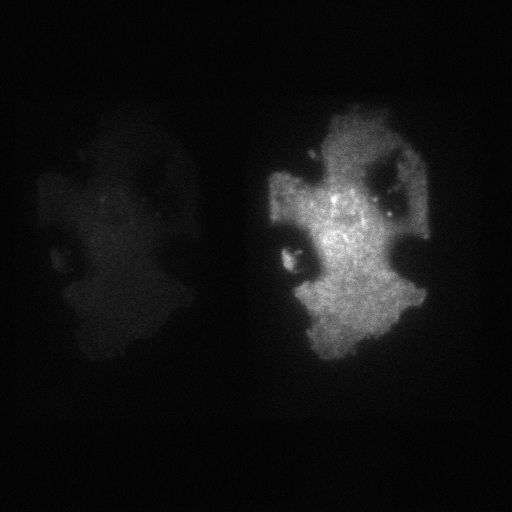

Supplement: Supplementary file 6 — Source Data for Figure 5 [file EMBR-24-e57842-s009.zip › Figure 5/F/118 nm diSav/green image after bleach.TIF]

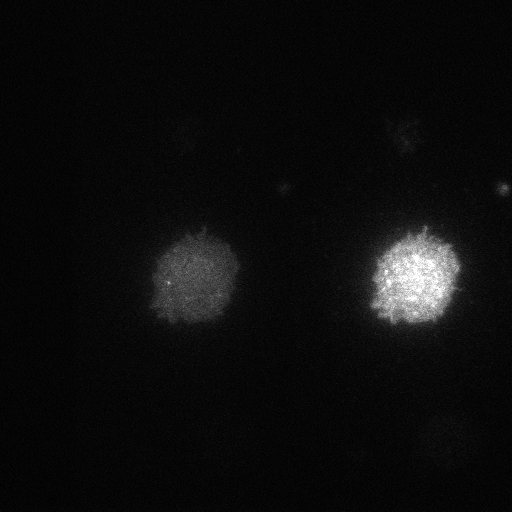

Supplement: Supplementary file 6 — Source Data for Figure 5 [file EMBR-24-e57842-s009.zip › Figure 5/D/Activated B-cell/green image before bleach.TIF]

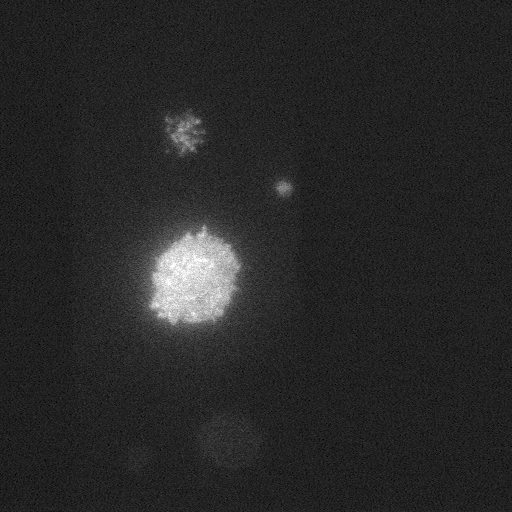

Supplement: Supplementary file 6 — Source Data for Figure 5 [file EMBR-24-e57842-s009.zip › Figure 5/D/Activated B-cell/red image before bleach.TIF]

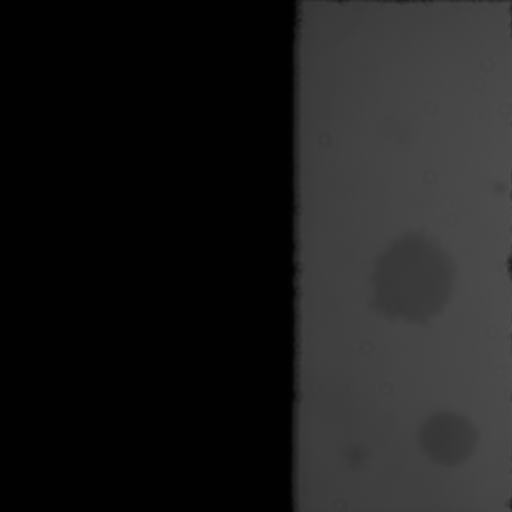

Supplement: Supplementary file 6 — Source Data for Figure 5 [file EMBR-24-e57842-s009.zip › Figure 5/D/Activated B-cell/IRM.tif]

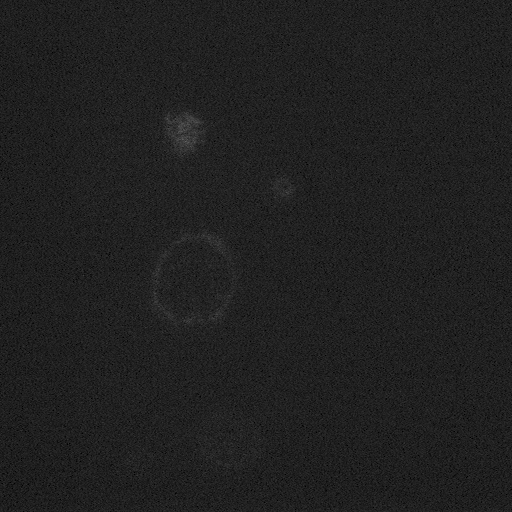

Supplement: Supplementary file 6 — Source Data for Figure 5 [file EMBR-24-e57842-s009.zip › Figure 5/D/Activated B-cell/red image after bleach.TIF]

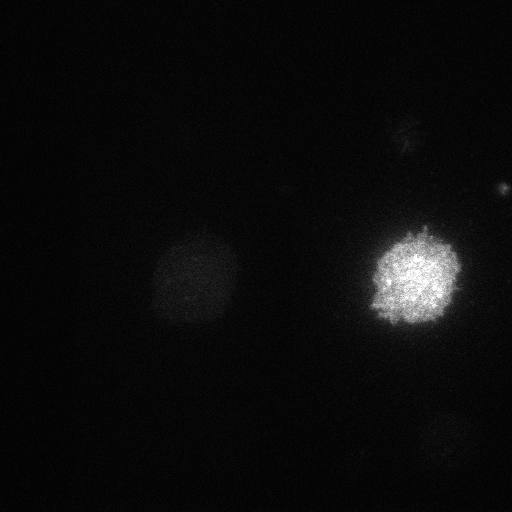

Supplement: Supplementary file 6 — Source Data for Figure 5 [file EMBR-24-e57842-s009.zip › Figure 5/D/Activated B-cell/green image after bleach.TIF]

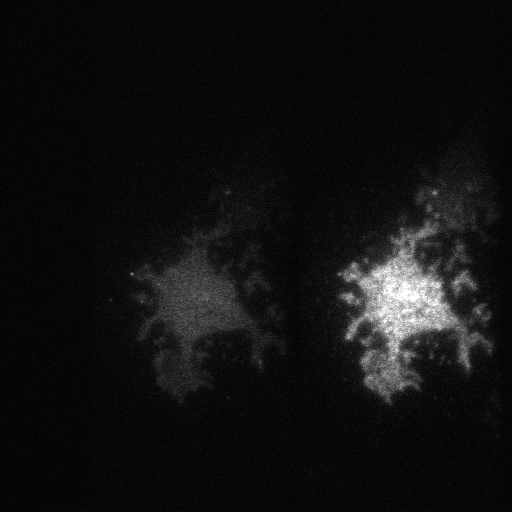

Supplement: Supplementary file 6 — Source Data for Figure 5 [file EMBR-24-e57842-s009.zip › Figure 5/D/Activated BMDC/green image before bleach.TIF]

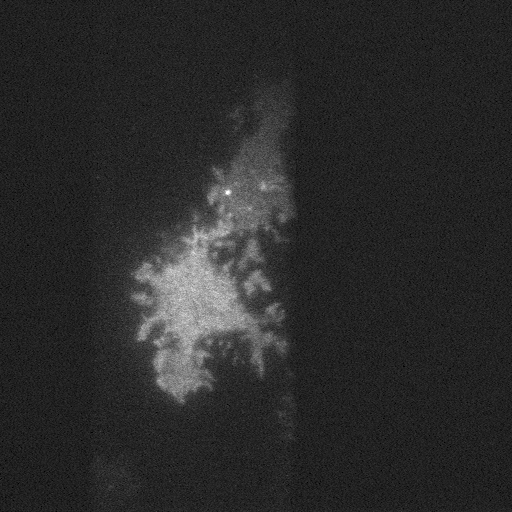

Supplement: Supplementary file 6 — Source Data for Figure 5 [file EMBR-24-e57842-s009.zip › Figure 5/D/Activated BMDC/red image before bleach.TIF]

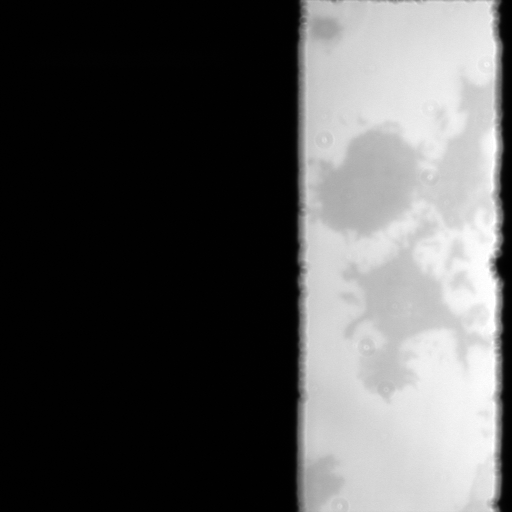

Supplement: Supplementary file 6 — Source Data for Figure 5 [file EMBR-24-e57842-s009.zip › Figure 5/D/Activated BMDC/IRM.tif]

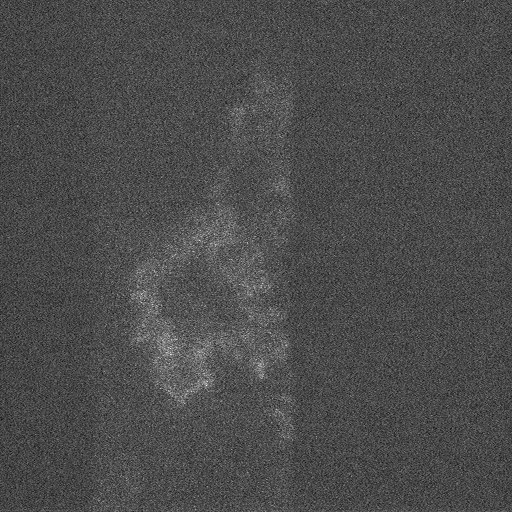

Supplement: Supplementary file 6 — Source Data for Figure 5 [file EMBR-24-e57842-s009.zip › Figure 5/D/Activated BMDC/red image after bleach.TIF]

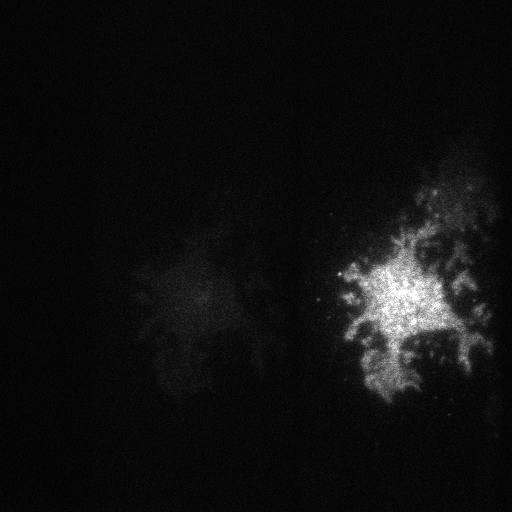

Supplement: Supplementary file 6 — Source Data for Figure 5 [file EMBR-24-e57842-s009.zip › Figure 5/D/Activated BMDC/green image after bleach.TIF]

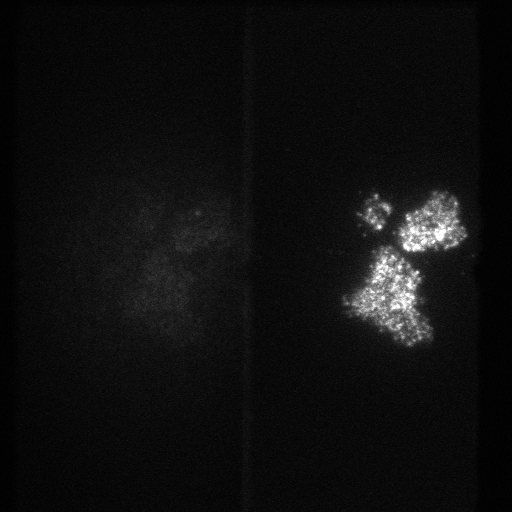

Supplement: Supplementary file 7 — Source Data for Figure 6 [file EMBR-24-e57842-s004.zip › Figure 6/F/example_cell_smFRET_stack.tif]

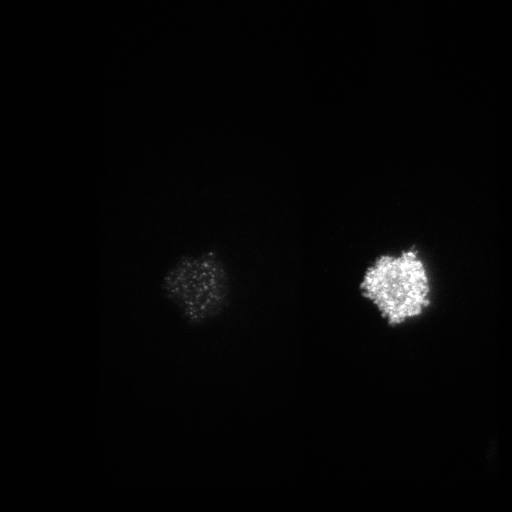

Supplement: Supplementary file 7 — Source Data for Figure 6 [file EMBR-24-e57842-s004.zip › Figure 6/E/green image before bleach.TIF]

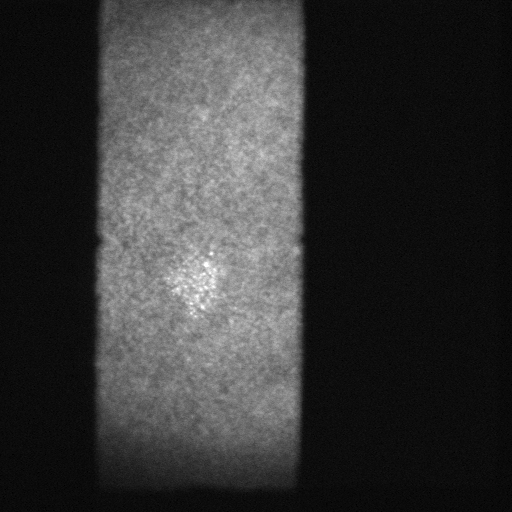

Supplement: Supplementary file 7 — Source Data for Figure 6 [file EMBR-24-e57842-s004.zip › Figure 6/E/red image before bleach.TIF]

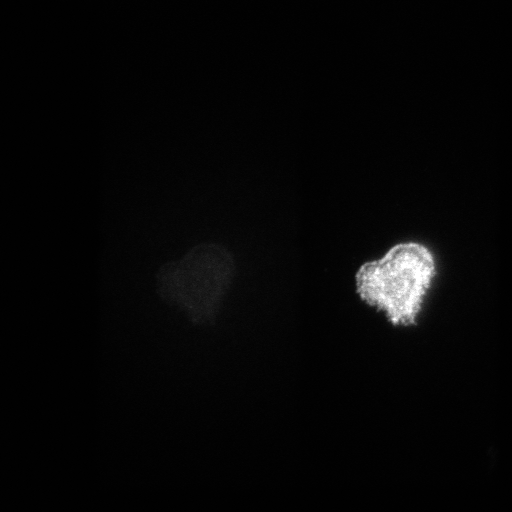

Supplement: Supplementary file 7 — Source Data for Figure 6 [file EMBR-24-e57842-s004.zip › Figure 6/E/example_cell_FRET_stack.tif]

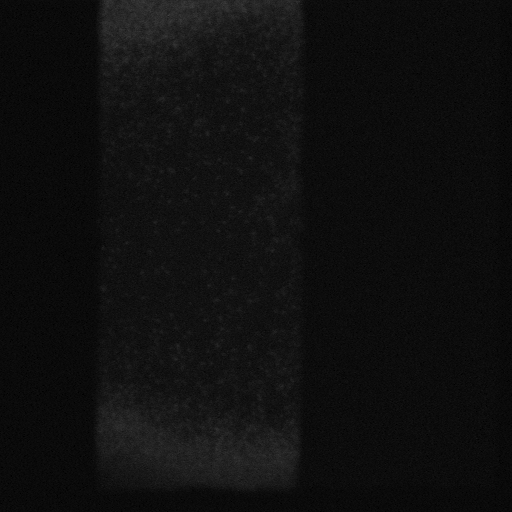

Supplement: Supplementary file 7 — Source Data for Figure 6 [file EMBR-24-e57842-s004.zip › Figure 6/E/red image after bleach.TIF]

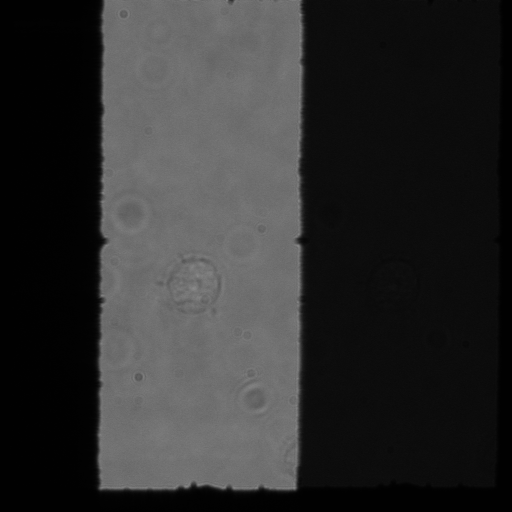

Supplement: Supplementary file 7 — Source Data for Figure 6 [file EMBR-24-e57842-s004.zip › Figure 6/E/example_cell_whitelight.tif]

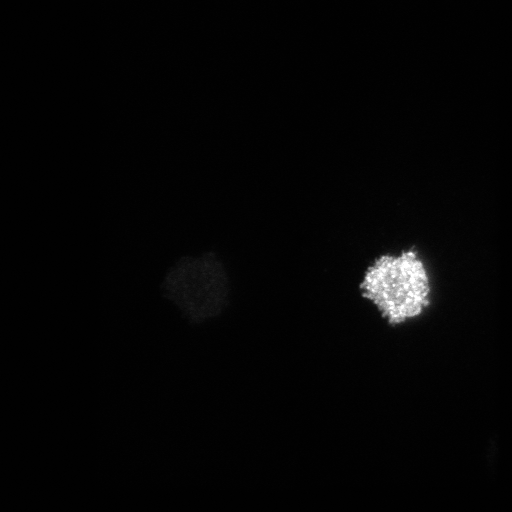

Supplement: Supplementary file 7 — Source Data for Figure 6 [file EMBR-24-e57842-s004.zip › Figure 6/E/green image after bleach.TIF]

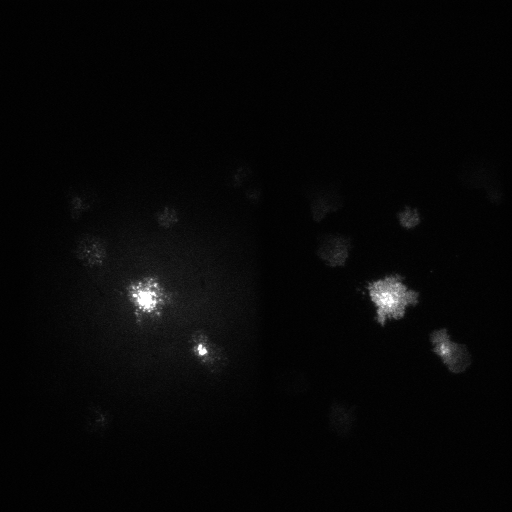

Supplement: Supplementary file 7 — Source Data for Figure 6 [file EMBR-24-e57842-s004.zip › Figure 6/B/green image before bleach.TIF]

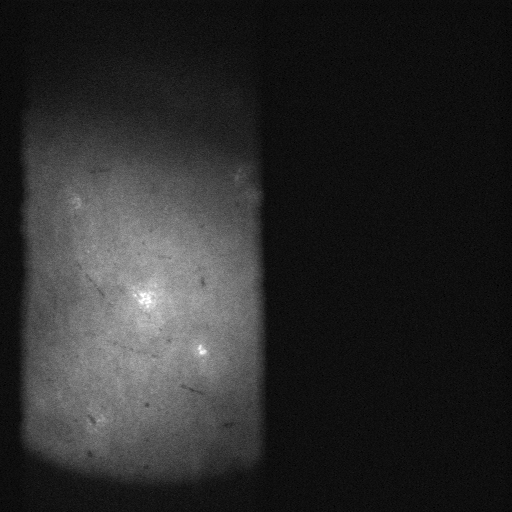

Supplement: Supplementary file 7 — Source Data for Figure 6 [file EMBR-24-e57842-s004.zip › Figure 6/B/red image before bleach.TIF]

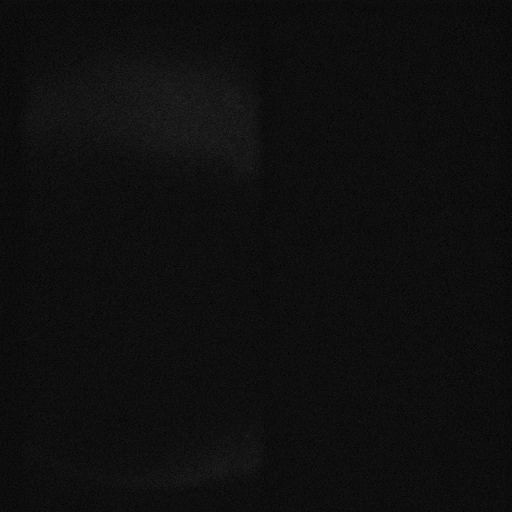

Supplement: Supplementary file 7 — Source Data for Figure 6 [file EMBR-24-e57842-s004.zip › Figure 6/B/red image after bleach.TIF]

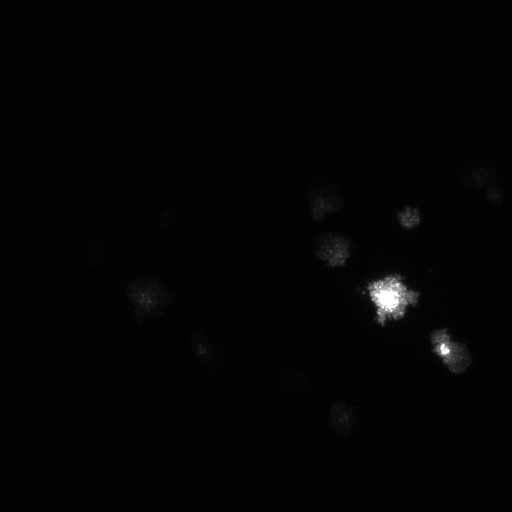

Supplement: Supplementary file 7 — Source Data for Figure 6 [file EMBR-24-e57842-s004.zip › Figure 6/B/green image after bleach.TIF]
