# Supplementary material for: Monomeric agonist peptide/MHCII complexes activate T‐cells in an autonomous fashion
Source: EMBO Rep. 2023 Sep 28;24(11):e57842. doi: 10.15252/embr.202357842 (PMC10626418; doi:10.15252/embr.202357842)
Supplement: Supplementary file 9 [file EMBR-24-e57842-s003.pdf]

EMBO reports

# Monomeric agonist peptide/MHCII complexes activate T-cells in an autonomous fashion

Rene Platzer, Joschka Hellmeier, Janett Göhring, Iago Doel Perez, Philipp Schatzlmaier, Clara Bodner, Margarete Focke-Tejkl, Gerhard Schutz, Eva Sevcsik, Hannes Stockinger, Mario Brameshuber, and Johannes Huppa
**DOI: 10.15252/embr.202357842**

*Corresponding author(s): Johannes Huppa (johannes.huppa@meduniwien.ac.at) , Mario Brameshuber (brameshuber@iap.tuwien.ac.at)*



## Review Timeline:

| | |
|---|---|
| Submission Date: | 19th Jul 23 |
| Editorial Decision: | 21st Jul 23 |
| Revision Received: | 7th Aug 23 |
| Editorial Decision: | 23rd Aug 23 |
| Revision Received: | 4th Sep 23 |
| Accepted: | 14th Sep 23 |

*Editor: Achim Breiling*


**Transaction Report: This manuscript was transferred to EMBO reports following peer review at The EMBO Journal.**

Dear Prof. Huppa,

Thank you for transferring your study to EMBO reports. I now went through your manuscript and the referee reports from The EMBO Journal (attached again below). The referees have several concerns and suggestions to improve the manuscript, or to strengthen the data and the conclusions drawn.

EMBO reports emphasizes novel functional over detailed mechanistic insight, but asks for strong in vivo relevance of the findings, and clear experimental support of the major conclusions. Thus, we will not require addressing points regarding more mechanistic details experimentally. However, it will be necessary that during revision you address all points questioning the main conclusions of the study, and all technical concerns, or points regarding the experimental designs, model systems used, or data presentation.

Given the constructive referee comments, I would like to invite you to revise your manuscript with the understanding that all concerns of the referees must be addressed in the revised manuscript or in a detailed point-by-point response as indicated above. Acceptance of your manuscript will depend on a positive outcome of another round of review at EMBO reports, using the same referees.

Revised manuscripts should be submitted within three months of a request for revision. Please contact me to discuss the revision (also by video chat) if you have questions or comments regarding the revision, or should you need additional time.

When submitting your revised manuscript, please also carefully review the instructions that follow below.

PLEASE NOTE THAT upon resubmission revised manuscripts are subjected to an initial quality control prior to exposition to re-review. Upon failure in the initial quality control, the manuscripts are sent back to the authors, which may lead to delays. Frequent reasons for such a failure are the lack of the data availability section (please see below) and the presence of statistics based on n=2 (the authors are then asked to present scatter plots or provide more data points).

When submitting your revised manuscript, we will require:

1) a .docx formatted version of the final manuscript text (including legends for main figures, EV figures and tables), but without the figures included. Please make sure that changes are highlighted to be clearly visible. Figure legends should be compiled at the end of the manuscript text.

2) individual production quality figure files as .eps, .tif, .jpg (one file per figure), of main figures and EV figures. Please upload these as separate, individual files upon re-submission. Please make sure that all figure panels are called out separately and sequentially in the manuscript text

The Expanded View format, which will be displayed in the main HTML of the paper in a collapsible format, has replaced the Supplementary information. You can submit up to 5 images as Expanded View. Please follow the nomenclature Figure EV1, Figure EV2 etc. The figure legend for these should be included in the main manuscript document file in a section called Expanded View Figure Legends after the main Figure Legends section. Additional Supplementary material should be supplied as a single pdf file labeled Appendix. The Appendix should have page numbers and needs to include a table of content on the first page (with page numbers) and legends for all content. Please follow the nomenclature Appendix Figure Sx, Appendix Table Sx etc. throughout the text, and also label the figures and tables according to this nomenclature.

For more details please refer to our guide to authors:
http://www.embopress.org/page/journal/14693178/authorguide#manuscriptpreparation

See also our guide for figure preparation:
http://wol-prod-cdn.literatumonline.com/pb-assets/embo-site/EMBOPress_Figure_Guidelines_061115-1561436025777.pdf

Moreover, please consult our guidelines for figure legend preparation:
https://www.embopress.org/page/journal/14693178/authorguide#figureformat

3) a .docx formatted letter INCLUDING the reviewers' reports and your detailed point-by-point responses to their comments. As part of the EMBO Press transparent editorial process, the point-by-point response is part of the Review Process File (RPF), which will be published alongside your paper.

4) a complete author checklist, which you can download from our author guidelines (https://www.embopress.org/page/journal/14693178/authorguide). Please insert page numbers in the checklist to indicate where the requested information can be found in the manuscript. The completed author checklist will also be part of the RPF.

Please also follow our guidelines for the use of living organisms, and the respective reporting guidelines:
http://www.embopress.org/page/journal/14693178/authorguide#livingorganisms

5) that primary datasets produced in this study (e.g. RNA-seq, ChIP-seq and array data) are deposited in an appropriate public database. This is now mandatory (like the COI statement). If no primary datasets have been deposited in any database, please state this in this section (e.g. 'No primary datasets have been generated and deposited').

See also: http://embor.embopress.org/authorguide#datadeposition

Please remember to provide a reviewer password if the datasets are not yet public.

The accession numbers and database should be listed in a formal "Data Availability " section (placed after Materials & Methods) that follows the model below. Please note that the Data Availability Section is restricted to new primary data that are part of this study.

# Data availability

The datasets produced in this study are available in the following databases:

- RNA-Seq data: Gene Expression Omnibus GSE46843 (https://www.ncbi.nlm.nih.gov/geo/query/acc.cgi?acc=GSE46843)
- [data type]: [name of the resource] [accession number/identifier/doi] ([URL or identifiers.org/DATABASE:ACCESSION])

*** Note - All links should resolve to a page where the data can be accessed. ***

Moreover, I have these editorial requests:

6) We now request the publication of original source data with the aim of making primary data more accessible and transparent to the reader. Our source data coordinator will contact you to discuss which figure panels we would need source data for and will also provide you with helpful tips on how to upload and organize the files.

7) Our journal encourages inclusion of *data citations in the reference list* to directly cite datasets that were re-used and obtained from public databases. Data citations in the article text are distinct from normal bibliographical citations and should directly link to the database records from which the data can be accessed. In the main text, data citations are formatted as follows: "Data ref: Smith et al, 2001" or "Data ref: NCBI Sequence Read Archive PRJNA342805, 2017". In the Reference list, data citations must be labeled with "[DATASET]". A data reference must provide the database name, accession number/identifiers and a resolvable link to the landing page from which the data can be accessed at the end of the reference. Further instructions are available at: http://www.embopress.org/page/journal/14693178/authorguide#referencesformat

8) Regarding data quantification and statistics, please make sure that the number "n" for how many independent experiments were performed, their nature (biological versus technical replicates), the bars and error bars (e.g. SEM, SD) and the test used to calculate p-values is indicated in the respective figure legends (also for potential EV figures and all those in the final Appendix). Please also check that all the p-values are explained in the legend, and that these fit to those shown in the figure. Please provide statistical testing where applicable. Please avoid the phrase 'independent experiment', but clearly state if these were biological or technical replicates. Please also indicate (e.g. with n.s.) if testing was performed, but the differences are not significant. In case n=2, please show the data as separate datapoints without error bars and statistics.
See also:
http://www.embopress.org/page/journal/14693178/authorguide#statisticalanalysis

If n<5, please show single datapoints for diagrams.

9) Please add scale bars of similar style and thickness to microscopic images, using clearly visible black or white bars (depending on the background). Please place these in the lower right corner of the images themselves. Please do not write on or near the bars in the image but define the size in the respective figure legend.

10) Please note our reference format:
http://www.embopress.org/page/journal/14693178/authorguide#referencesformat

11) We updated our journal's competing interests policy in January 2022 and request authors to consider both actual and perceived competing interests. Please review the policy https://www.embopress.org/competing-interests and add a statement declaring your competing interests. Please name that section 'Disclosure and Competing Interests Statement' and add it after the author contributions section.

12) Please order the manuscript sections like this using these names:
Title page - Abstract - Keywords - Introduction - Results - Discussion - Materials and Methods - Data availability section (DAS) -

Acknowledgements - Disclosure and Competing Interests Statement - References - Figure legends - Expanded View Figure legends

13) Please make sure that all the funding information is also entered into the online submission system and is complete and similar to the one in the manuscript text file (in the Acknowledgements).

14) We now use CRediT to specify the contributions of each author in the journal submission system. CRediT replaces the author contribution section. Please use the free text box to provide more detailed descriptions. Thus, please remove the author contributions section from the manuscript text file. See also guide to authors:
https://www.embopress.org/page/journal/14693178/authorguide#authorshipguidelines

15) We would encourage you to use 'Structured Methods', our new Materials and Methods format. According to this format, the Materials and Methods section should include a Reagents and Tools Table (listing key reagents, experimental models, software and relevant equipment and including their sources and relevant identifiers) followed by a Methods and Protocols section in which we encourage the authors to describe their methods using a step-by-step protocol format with bullet points, to facilitate the adoption of the methodologies across labs. More information on how to adhere to this format as well as downloadable templates (.doc or .xls) for the Reagents and Tools Table can be found in our author guidelines (section 'Structured Methods'):

https://www.embopress.org/page/journal/14693178/authorguide#manuscriptpreparation

I look forward to seeing a revised version of your manuscript when it is ready. Please let me know if you have questions or comments regarding the revision.

Kind regards,

Achim

--------------------
Achim Breiling
Senior Editor
EMBO Reports
--------------------

Referee #1:

Here, Platzer et al use a combination of methods - especially super-resolution imaging and single molecule imaging to show that pMHC on the cell surface is mainly monomeric and fast diffusing. Technically, I think the paper is very good. It's thorough and well written. I'm not entirely sure about the novelty of these findings - the authors seem to imply the monomeric nature of pMHC is surprising, but I'm not sure it is. For one thing, I see that the authors have published similar findings for other immune cell signalling molecules in the past. I could say something similar about the rapid diffusion characteristics. For example they seem to observe diffusion coefficients around 0.2-0.3 um^2/s which is probably what I would have expected. Overall... I think the novelty of the findings is borderline but the paper is technically excellent.

Like I said, technically, I thought the paper was very good. I just didn't understand some of the clustering and interpretation, mainly in Figure 2:
Can the authors justify the method of "correcting" for multiple blinking via simulation? It seems simulations could easily miss biophysical phenomena that could affect the localisations. As this is PALM data, it should be correctable for multiple-blinking using the method of Jensen et al or Bohrer et al, both Nature Methods. In fact, the Jensen paper is explicitly used to test for randomness when combined with the Ripley's K-function analysis they perform here.
I'm curious about the Ripley's K-function curves in 2B. The authors claim there are no "significant" differences between the data and randomly distributed simulations. Is this the curve for 1 ROI or a whole condition? Ripley's is normalised to density so they should be summable to get the condition Ripley-K-function? Or all the curves can be shown? In any case, I don't understand how the p-values were extracted from these curves - for example, in the activated cells the data does actually leave the simulated envelope. Would that not mean there is a significant difference?
I also don't really understand the further cluster analysis in 2 E and F. If Ripley's K-function have already demonstrate the presence of clustering (or not) what do the plots in E and F add? Why not instead perform conventional cluster analysis on those datasets showing positive Ripley's results e.g. by DBSCAN or some other method?

I note also there is a relatively high abundance of self-references...

----
Referee #2:

'Monomeric agonist peptide/MHCII complexes activate T-cells in an autonomous fashion' by Platzer et al. report that pMHC-II complexes are single entities that rapidly diffuse in the plasma membrane of antigen-presenting cells. The presence of bystander-pMHCII previously termed "co-agonist pMHC" affected neither synaptic agonist TCR-binding nor efficiencies of T-cell recognition.

The authors used advanced imaging to revisit the distribution of MHC-II molecules on the cell surface of living APCs. Surprisingly, they found monomers of MHC-II, which is in contrast to multiple independent studies that observed MHC-II dimers, multimers and higher order structures. These former studies include observations of MHC dimer/cluster formation in living cells using single molecule tracking, super-resolution imaging and resolving the crystal structure of MHC-II as a dimer.

The data in the current paper look convincing and proper controls and validation of the reagents are included. The authors did a great job in imaging endogenous MHC-II molecules on living primary APC. They validated their findings with different methods/analysis methods and analyzed two different primary cell types (B cells and BMDCs). The live cell imaging experiments are technically challenging, and the figures include excellent informative cartoons to explain the different experimental set-ups.

Altogether, the experiments are really well performed and the central question is why their results are so contradictory to previous studies. In particular, why did former studies using the same set-up (single molecule tracking studies on live cells) show MHC clusters at the cell surface? Although different factors may have contributed to this (fixation, cell model, imaging methods), it leaves the reader a bit puzzled in the end.

The limitations of the current study are: usage of murine cells only (no human cells), imaging all done with one MHC-II nanobody (14.4.4 sc), one type of coating (ICAM-1), usage of an artificial lipid bilayer with only 1 lipid species (POPC). At the same time, the paper is already very elaborate (18 suppl. figures), and including more data to address these limitations will, most likely, not change the main message.

In summary, this paper revisited MHC clustering and the pseudo-dimer model and comes to a different conclusion. The findings are against the dogma which makes it interesting, yet puzzling. How will these findings advance the field and what are the consequences for immune responses? I leave it to the Editor to decide whether EMBO Journal finds this message of significant novelty.

----
Referee #3:

In this paper the authors describe the use of a range of methods to address and important outstanding question about T cell receptor triggering. This is the extent to which pMHC ligand engagement functions to dimerise/aggregate the TCR.
The authors used two forms of super resolution microscopy (SMLM and STED) and FRET to show that pMHC class II molecules are randomly distributed on antigen presenting cells (B cells and BDDC) and present as single molecules from the moment they appear on the self surface.

They go on to show that the presence of other low affinity pMHC molecules has no impact on TCR engagement of agonist pMHC and that agonist pMHC are most effective at activating T cells when present as single monomeric molecules.

This invaluable study convincingly shows that pMHC molecules are monomeric on the self surface of APCs and activate T cells best as monomers. When taken together with other studies by the same group demonstrating that the TCR is also a monomer both before and after TCR triggering, these results place important constraints on the mechanism of TCR triggering.

This is an extremely well executed and convincing study which represents an important advance in our understanding of TCR triggering.

I read the paper twice and was unable to find any significant errors other than minor typos.

**<u>Point by point response</u> to editor's and referees´ comments relating to Platzer et al. titled "Monomeric agonist peptide/MHCII complexes activate T-cells in an autonomous fashion" (EMBOR-2023-57842)**

1) a .docx formatted version of the final manuscript text (including legends for main figures, EV figures and tables), but without the figures included. Please make sure that changes are highlighted to be clearly visible. Figure legends should be compiled at the end of the manuscript text.

*→ We highlighted all changes made in the ms. text* in red color. *Our responses in this document are initiated with an arrow (→) written in italics and blue fonts.*

2) individual production quality figure files as .eps, .tif, .jpg (one file per figure), of main figures and EV figures. Please upload these as separate, individual files upon re-submission. Please make sure that all figure panels are called out separately and sequentially in the manuscript text.
The Expanded View format, which will be displayed in the main HTML of the paper in a collapsible format, has replaced the Supplementary information. You can submit up to 5 images as Expanded View. Please follow the nomenclature Figure EV1, Figure EV2 etc. The figure legend for these should be included in the main manuscript document file in a section called Expanded View Figure Legends after the main Figure Legends section. Additional Supplementary material should be supplied as a single pdf file labeled Appendix. The Appendix should have page numbers and needs to include a table of content on the first page (with page numbers) and legends for all content. Please follow the nomenclature Appendix Figure Sx, Appendix Table Sx etc. throughout the text, and also label the figures and tables according to this nomenclature.
For more details please refer to our guide to authors:
http://www.embopress.org/page/journal/14693178/authorguide#manuscriptpreparation
See also our guide for figure preparation:
http://wol-prod-cdn.literatumonline.com/pb-assets/embo-site/EMBOPress_Figure_Guidelines_061115-1561436025777.pdf
Moreover, please consult our guidelines for figure legend preparation: https://www.embopress.org/page/journal/14693178/authorguide#figureformat

*→ We labeled all Figures, EV Figures and Appendix Figures in the text and legends as requested. We included page numbers and a table of content in the Appendix.*

*Fig 1 + Fig EV1, EV2, EV3*
*Fig 2 + Fig EV4, EV5, EV6, EV7, EV8*
*Fig 3 + Fig EV9*
*Fig 4 + Fig EV10*
*Fig 5 + Fig EV11*
*Fig 6 + Fig EV12, EV13, EV14*
*Fig 7 + Fig EV15, EV16*

*Appendix Figure S1-4*
*Appendix Table S1*

3) a .docx formatted letter INCLUDING the reviewers' reports and your detailed point-by-point responses to their comments. As part of the EMBO Press transparent editorial process, the point-by-point response is part of the Review Process File (RPF), which will be published alongside your paper.

*→ Done.*

4) a complete author checklist, which you can download from our author guidelines (https://www.embopress.org/page/journal/14693178/authorguide). Please insert page numbers in the checklist to indicate where the requested information can be found in the manuscript. The completed author checklist will also be part of the RPF.
Please also follow our guidelines for the use of living organisms, and the respective reporting guidelines: http://www.embopress.org/page/journal/14693178/authorguide#livingorganisms

→ *We completed the author checklist.*

5) that primary datasets produced in this study (e.g. RNA-seq, ChIP-seq and array data) are deposited in an appropriate public database. This is now mandatory (like the COI statement). If no primary datasets have been deposited in any database, please state this in this section (e.g. 'No primary datasets have been generated and deposited').
See also: http://embor.embopress.org/authorguide#datadeposition
Please remember to provide a reviewer password if the datasets are not yet public.
The accession numbers and database should be listed in a formal "Data Availability " section (placed after Materials & Methods) that follows the model below. Please note that the Data Availability Section is restricted to new primary data that are part of this study.

# Data availability
The datasets produced in this study are available in the following databases:
- RNA-Seq data: Gene Expression Omnibus GSE46843 (https://www.ncbi.nlm.nih.gov/geo/query/acc.cgi?acc=GSE46843)
- [data type]: [name of the resource] [accession number/identifier/doi] ([URL or identifiers.org/DATABASE:ACCESSION])
*** Note - All links should resolve to a page where the data can be accessed. ***

→ *Source data are provided with the manuscript. The datasets generated and/or analyzed are available from the corresponding authors upon reasonable request. Links to the software packages for analysis can be obtained from the cited papers or from the corresponding authors. No additional software packages or primary datasets have been generated and deposited.*

6) We now request the publication of original source data with the aim of making primary data more accessible and transparent to the reader. Our source data coordinator will contact you to discuss which figure panels we would need source data for and will also provide you with helpful tips on how to upload and organize the files.

→ *We attached the source data for all main Figures as requested. In addition, we attached a source data file containing numerical data for all Figures, EV Figures and Appendix Figures in a single Excel sheet.*

7) Our journal encourages inclusion of *data citations in the reference list* to directly cite datasets that were re-used and obtained from public databases. Data citations in the article text are distinct from normal bibliographical citations and should directly link to the database records from which the data can be accessed. In the main text, data citations are formatted as follows: "Data ref: Smith et al, 2001" or "Data ref: NCBI Sequence Read Archive PRJNA342805, 2017". In the Reference list, data citations must be labeled with "[DATASET]". A data reference must provide the database name, accession number/identifiers and a resolvable link to the landing page from which the data can be accessed at the end of the reference. Further instructions are available at: http://www.embopress.org/page/journal/14693178/authorguide#referencesformat

→ *We did not reuse datasets from public databases.*

8) Regarding data quantification and statistics, please make sure that the number "n" for how many independent experiments were performed, their nature (biological versus technical replicates), the bars and error bars (e.g. SEM, SD) and the test used to calculate p-values is indicated in the respective figure legends (also for potential EV figures and all those in the final Appendix). Please also check that all the p-values are explained in the legend, and that these fit to those shown in the figure. Please provide statistical testing where applicable. Please avoid the phrase 'independent experiment', but clearly state if these were biological or technical replicates. Please also indicate (e.g. with n.s.) if testing was performed, but the differences are not significant. In case n=2, please show the data as separate datapoints without error bars and statistics.
See also:
http://www.embopress.org/page/journal/14693178/authorguide#statisticalanalysis

If n<5, please show single datapoints for diagrams.

→ *Done.*

9) Please add scale bars of similar style and thickness to microscopic images, using clearly visible black or

white bars (depending on the background). Please place these in the lower right corner of the images themselves. Please do not write on or near the bars in the image but define the size in the respective figure legend.

*→ Done.*

10) Please note our reference format:
http://www.embopress.org/page/journal/14693178/authorguide#referencesformat

*→ We changed the reference format to Harvard style.*

11) We updated our journal's competing interests policy in January 2022 and request authors to consider both actual and perceived competing interests. Please review the policy https://www.embopress.org/competing-interests and add a statement declaring your competing interests. Please name that section 'Disclosure and Competing Interests Statement' and add it after the author contributions section.

*→ Done. The authors declare no competing interests.*

12) Please order the manuscript sections like this using these names:
Title page - Abstract - Keywords - Introduction - Results - Discussion - Materials and Methods - Data availability section (DAS) - Acknowledgements - Disclosure and Competing Interests Statement - References - Figure legends - Expanded View Figure legends

*→ We ordered the ms. sections as requested.*

13) Please make sure that all the funding information is also entered into the online submission system and is complete and similar to the one in the manuscript text file (in the Acknowledgements).

*→ Done.*

14) We now use CRediT to specify the contributions of each author in the journal submission system. CRediT replaces the author contribution section. Please use the free text box to provide more detailed descriptions. Thus, please remove the author contributions section from the manuscript text file. See also guide to authors:
https://www.embopress.org/page/journal/14693178/authorguide#authorshipguidelines

*→ We removed the author contribution section from the ms text file as requested.*

15) We would encourage you to use 'Structured Methods', our new Materials and Methods format. According to this format, the Materials and Methods section should include a Reagents and Tools Table (listing key reagents, experimental models, software and relevant equipment and including their sources and relevant identifiers) followed by a Methods and Protocols section in which we encourage the authors to describe their methods using a step-by-step protocol format with bullet points, to facilitate the adoption of the methodologies across labs. More information on how to adhere to this format as well as downloadable templates (.doc or .xls) for the Reagents and Tools Table can be found in our author guidelines (section 'Structured Methods'):

*→ We included a "Reagents and Tools Table" (p. 31 – 37) and explained our methods in detail (p. 38 – 70).*

https://www.embopress.org/page/journal/14693178/authorguide#manuscriptpreparation

I look forward to seeing a revised version of your manuscript when it is ready. Please let me know if you have questions or comments regarding the revision.

Please use this link to submit your revision: https://embor.msubmit.net/cgi-bin/main.plex

**Point-by-point response to reviewers' comments**
(Changes made to the ms. in response to the reviewers' comments are marked in red, while our responses to the comments are *initiated with an arrow (→), written in italics and colored in blue*.

**Referee #1:**

Here, Platzer et al use a combination of methods - especially super-resolution imaging and single molecule imaging to show that pMHC on the cell surface is mainly monomeric and fast diffusing. Technically, I think the paper is very good. It's thorough and well written.

*→ We thank reviewer #1 for her/his time, valuable insights and positive feedback.*

I'm not entirely sure about the novelty of these findings - the authors seem to imply the monomeric nature of pMHC is surprising, but I'm not sure it is.

*→ In fact, Ref. #2 was very surprised by our findings. And we are not aware of any study that describes comprehensively the monomeric (or multimeric) nature of MHCII on living cells using single-molecule microscopy techniques.*

For one thing, I see that the authors have published similar findings for other immune cell signalling molecules in the past. I could say something similar about the rapid diffusion characteristics. For example they seem to observe diffusion coefficients around 0.2-0.3 um^2/s which is probably what I would have expected. Overall... I think the novelty of the findings is borderline but the paper is technically excellent.

*→ We are certainly aware of previous MHC-tracking studies conducted with many different cell types using different molecular probes (e.g. summarized in Umemura et al 2008 Biophysical Journal). We would like to emphasize though that our study, while it incorporates single molecule tracking, deals with the much larger issue of spatiotemporal organization of (even newly-arriving) MHCII on living cells in a comprehensive manner in a fashion no other study (to our knowledge) has done, employing superresolution microscopy, (PALM, STED), FRET, TOCCSL (stoichiometry), biochemical experimentation (combined with flow cytometry) as well as functional T-cell studies focused on whether and how endogenous peptide-MHCIIs impact the recognition of rare antigens. Not a single study has provided such detail and depth.*
*Regarding our previous studies on the distribution and stoichiometry of the TCR (Brameshuber et al 2018, and Rossboth et al 2018, both Nature Immunology), we are not exactly sure what the reviewer is referring to. More specifically, why should MHCII molecules on APCs feature the same biophysical parameters as TCRs on T-cells? TCRs are much larger complexes (together with CD3) and diffuse as a consequence and also due to cytoskeletal interaction considerably slower (~ 0.05 $\mu m^2/s$) with a mobile fraction of only ~64% (Brameshuber et al 2018, Nature Immunology).*

Like I said, technically, I thought the paper was very good. I just didn't understand some of the clustering and interpretation, mainly in Figure 2:
Can the authors justify the method of "correcting" for multiple blinking via simulation? It seems simulations could easily miss biophysical phenomena that could affect the localisations.

*→ We have included the parameters of (i) molecular density, (ii) lateral diffusion (fast- and slow-moving molecules at 25 °C) and (iii) PS-CFP2 blinking (recorded under the same imaging conditions as the cell data) in our simulations. Of note, a straightforward analysis can easily introduce artifacts as "biophysical phenomena". Using experimentally derived (and close to the ground-truth) blinking statistics is in our view the best way to deal with any kinds of blinking artifacts. As pointed out in Platzer et al 2020 (Nature Communications), the correction even accounts for outliers, which is not the case for other algorithms.*

*→ Interestingly and of relevance for the argument made by the reviewer, Jensen et al discussed our techniques (Baumgart et al 2016, Nature Methods, and Platzer et al 2020, Nature Communications) as tools to comprehensively analyze and test spatial randomness in PALM data. The authors mentioned the limitations of our approach as (i) to be time-consuming, (ii) complex and (iii) to require a substantial experimental effort, such as determining the cell-surface density and diffusion constant of MHCII and PS-CFP2 blinking data for our simulations. We are aware that our techniques do not provide a corrected set of localization maps (i.e.*

*ground* truth localizations without blinking), which can be further characterized with conventional cluster analysis tools (e.g. DBSCAN).

As this is PALM data, it should be correctable for multiple-blinking using the method of Jensen et al or Bohrer et al, both Nature Methods. In fact, the Jensen paper is explicitly used to test for randomness when combined with the Ripley's K-function analysis they perform here.

*→ In addition to presenting the results of our analytical approach, we have now included the model-based correction introduced by Jensen et al (Jensen et al 2022 Nature Methods) in the revised version of our ms. (**Fig EV6A-G**). We have explained all necessary steps in the method section (**p. 55**).*

I'm curious about the Ripley's K-function curves in 2B. The authors claim there are no "significant" differences between the data and randomly distributed simulations. Is this the curve for 1 ROI or a whole condition?

*→ We only compared Ripley's K (L(r)-r) maxima (similar as was done in Jensen et al) of recorded and simulated PALM data, not the shape of the entire curve. We now described in more depth and detail the underlying principle of this approach in the main text (**p. 10**), methods section (**p. 54**) and **Fig EV6AB**, and how we end up defining differences to spatial randomness. We did not perform a significant test on our sample and simulated data, so we removed the term "significant" in the section. Of note, this analysis technique was justified and thoroughly tested in Platzer et al 2020 (Nature Communications). In addition, we summarized all investigated cell regions in a plot (**Fig EV6C**).*

Ripley's is normalised to density so they should be summable to get the condition Ripley-K-function? Or all the curves can be shown? In any case, I don't understand how the p-values were extracted from these curves - for example, in the activated cells the data does actually leave the simulated envelope. Would that not mean there is a significant difference?

*→ We included summarized Ripley's K functions of the model-based corrected (MBC) localization maps of I-$E^k$ on (living and PFA-fixed) BMDCs and B-cells, as well as DEC205 on BMDCs (**Fig EV6D and 6F**). As mentioned above, we did not extract p-values from our analysis. We compared the max L(r) -r value with the mean +/- standard deviation of 100 simulations representing spatial randomness as described in the method section (**p. 54**) and in **Fig EV6AB**. We next analyzed all regions (with L(r)-r above and within the simulation) using DBSCAN as described in the method section (**p. 56**).*

I also don't really understand the further cluster analysis in 2 E and F. If Ripley's K-function have already demonstrated the presence of clustering (or not) what do the plots in E and F add? Why not instead perform conventional cluster analysis on those datasets showing positive Ripley's results e.g. by DBSCAN or some other method?

*→ We have provided a more thorough explanation of the varying labeling density approach (Baumgart et al 2016, Nature Methods) in the main text (**p. 11**), which is an unbiased complementary method to determine molecular clustering over spatial randomness using label density variation.*

*→ We have also applied conventional cluster analysis on all ground truth datasets after MBC using DBSCAN and presented the results in Fig EV6E (for living cells) and Fig EV6F (for PFA-fixed cells). We further explained DBSCAN analysis in the methods section (**p. 56**).*

I note also there is a relatively high abundance of self-references...

*→ This is certainly true for the section dealing with superresolution (PALM and STED).*
*We employed techniques for cluster analysis published in Platzer et al 2020 (Nature Communications), Baumgart et al 2016 (Nature Methods) and Rossboth et al 2018 (Nature Immunology), as well as Sengupta et al 2011 (Nature Methods). We included now the model-based correction (Jensen et al 2022 or Bohrer et al 2021, both Nature Methods), and analyzed the corrected datasets with DBSCAN (Ester et al 1996, In Proceedings of the Second International Conference on Knowledge Discovery in Databases and Data Mining).*

*→ We also employed a FRET-based readout (Huppa et al 2010, Nature), TOCCSL (Brameshuber et al 2018, Nature Immunology), DNA-origamis (Hellmeier et al 2021 PNAS; Hellmeier et al 2021 ACS Nano) and LGC flow cytometry (Schatzlmaier et al 2015, Science Signaling), which had all been developed in-house or in close collaboration with the Institute of Applied Physics at the TU Vienna.*

**Referee #2:**

'Monomeric agonist peptide/MHCII complexes activate T-cells in an autonomous fashion' by Platzer et al. reports that pMHC-II complexes are single entities that rapidly diffuse in the plasma membrane of antigen-presenting cells. The presence of bystander-pMHCII previously termed "co-agonist pMHC" affected neither synaptic agonist TCR-binding nor efficiencies of T-cell recognition.

The authors used advanced imaging to revisit the distribution of MHC-II molecules on the cell surface of living APCs. Surprisingly, they found monomers of MHC-II, which is in contrast to multiple independent studies that observed MHC-II dimers, multimers and higher order structures. These former studies include observations of MHC dimer/cluster formation in living cells using single molecule tracking, super-resolution imaging and resolving the crystal structure of MHC-II as a dimer.

The data in the current paper look convincing and proper controls and validation of the reagents are included. The authors did a great job in imaging endogenous MHC-II molecules on living primary APC. They validated their findings with different methods/analysis methods and analyzed two different primary cell types (B cells and BMDCs). The live cell imaging experiments are technically challenging, and the figures include excellent informative cartoons to explain the different experimental set-ups.

*→ We thank reviewer # 2 for her/his time, valuable suggestions and overall positive feedback.*
*→ In fact, Ref. #1 was not surprised by our findings. It seems that the spatiotemporal organization of MHCII, especially on living APCs, is not completely understood, which prompted us to undertake the study.*

Altogether, the experiments are really well performed and the central question is why their results are so contradictory to previous studies. In particular, why did former studies using the same set-up (single molecule tracking studies on live cells) show MHC clusters at the cell surface? Although different factors may have contributed to this (fixation, cell model, imaging methods), it leaves the reader a bit puzzled in the end.

*→ We are certainly aware that for example Umemura et al 2008 (Biophysical Journal) used I-E$^k$-reactive Fabs and anti- IgG Fab-conjugated gold particles (40 nm in size) to track I-Ek on CHO cells and found confinement zones of ~40 nm with ~1-3 ms residence time in every compartment when using a frame time of 20 µs. Similar to Vrljic et al 2002 (Biophysical journal) and Nishimura et al 2006 (Biophysical journal), they also tracked I-E$^k$ with an (Alexa594-conjugated) Fab fragment at time frames of 33 ms and extracted for I-E$^k$ a lateral diffusion of ~ 0.15 µm$^2$/s at 37 °C, which is in large part comparable to what our measurements showed. Giving the moderate to fast photobleaching rate of the organic fluorophores used in our study, we cannot track at frame rates of 20 µs. Instead. we aimed for a small molecular probe to maximally maintain the integrity and natural diffusion behavior of the target molecule and its interaction with other surface molecules.*

*This is why we carried out our analysis with a smaller, fluorescently-labeled scF$_V$ as well as with streptavidin (+ biotinylated peptide) on activated BMDCs and B-cells (as opposed to CHO cells as employed by Vrljic and Nishimura in 2002 and in 2006). Of note, we also observed signs of confinement with a radius of smaller than 75 nm for slow-moving MHCII molecules (at a frame time of 10 ms), as mentioned in the text. We did not interpret these confinement zones as MHCII clusters per se, but used other techniques (PALM, STED) to describe the spatial distribution of the entire surface MHCII population.*

*It should be noted that the surface density of I-E$^k$ (and I-A$^k$) is unusually high (up to 2,000 molecules per µm$^2$), which corresponds to ~ 90 % of randomly distributed molecules already within a nearest-neighbor distance of 20 nm. The question remains whether further enrichment of MHCII in 40 nm compartments with a 1-3 ms residence time is exerting a sensitizing effect on T-cells, especially since physiological TCR-pMHC binding half-lives are in the range of hundreds of ms to tens of seconds. As T-cells react to single, randomly distributed antigenic pMHC molecules on planar lipid bilayers (in the presence of ICAM-1 and B7-1), we conclude that MHCII compartmentalization is irrelevant for T-cell sensitization.*

The limitations of the current study are: usage of murine cells only (no human cells), imaging all done with one MHC-II nanobody (14.4.4 sc), one type of coating (ICAM-1), usage of an artificial lipid bilayer with only 1 lipid species (POPC). At the same time, the paper is already very elaborate (18 suppl. figures), and including more data to address these limitations will, most likely, not change the main message.

*→ We agree with this reviewer. Importantly, we have also carried out measurements with the combined use of biotinylated peptides (for MHC loading) and monovalent streptavidin (for surface detection), which have*

*yielded comparable if not identical results. We agree with the reviewer that adding more data and surfaces will most likely not change the main message. Hence we have refrained from doing so.*

In summary, this paper revisited MHC clustering and the pseudo-dimer model and comes to a different conclusion. The findings are against the dogma which makes it interesting, yet puzzling. How will these findings advance the field and what are the consequences for immune responses? I leave it to the Editor to decide whether EMBO Journal finds this message of significant novelty.

*→ We provided a elaborate explanation in the introduction and discussion sections why we think our study is innovative even though (or just because) it deals with some of the most basic aspects of immune recognition, which is undeniably only poorly understood. Filling such deficits with insights is not only worth in its own academic right but will in our view make the decisive difference when devising T-cell-based therapies, which have not yet reached the level of efficacy where we need them.*

----
Referee #3:

In this paper the authors describe the use of a range of methods to address and important outstanding question about T cell receptor triggering. This is the extent to which pMHC ligand engagement functions to dimerise/aggregate the TCR.
The authors used two forms of super resolution microscopy (SMLM and STED) and FRET to show that pMHC class II molecules are randomly distributed on antigen presenting cells (B cells and BDDC) and present as single molecules from the moment they appear on the self surface.

They go on to show that the presence of other low affinity pMHC molecules has no impact on TCR engagement of agonist pMHC and that agonist pMHC are most effective at activating T cells when present as single monomeric molecules.

This invaluable study convincingly shows that pMHC molecules are monomeric on the self surface of APCs and activate T cells best as monomers. When taken together with other studies by the same group demonstrating that the TCR is also a monomer both before and after TCR triggering, these results place important constraints on the mechanism of TCR triggering.

This is an extremely well executed and convincing study which represents an important advance in our understanding of TCR triggering.

I read the paper twice and was unable to find any significant errors other than minor typos.

*→ We would like to thank this reviewer for her/his time, efforts and highly positive opinion.*

**1st Revision - Editorial Decision** **23rd Aug 2023**

Dear Prof. Huppa,

Thank you for the submission of your revised manuscript to our editorial offices. I have now received the reports from the two referees that I asked to re-evaluate your study, you will find below. As you will see, both referees are satisfied by the revision and support publication of the manuscript in EMBO reports.

Before proceeding with formal acceptance, I have these editorial requests I ask you to address in a final revised manuscript:

- Please provide the abstract written in present tense.

- You can submit only up to 5 images as Expanded View. Additional Supplementary material should be supplied as a single pdf file labeled Appendix. The Appendix should have page numbers and needs to include a table of content on the first page (with page numbers) and legends for all content. Please follow the nomenclature Appendix Figure Sx, Appendix Table Sx etc. throughout the text, and also label the figures and tables according to this nomenclature.

For more details, please refer to our guide to authors:
http://www.embopress.org/page/journal/14693178/authorguide#manuscriptpreparation

In this case, please either select 5 of the 16 EV figures as final EV figures and move the remainder into the Appendix, or move all the 16 figures into the Appendix (which might make more sense). In any case, please update all the callouts after doing these changes.

Please also make sure that all figure panels are called out separately and sequentially. Presently, Fig. EV5 is called out before EV4. Please check.

- In the Acknowledgements section P32307-B (JG) is mentioned twice. Please check.

- It seems that the panels of Figure 1A are also shown in Figure EV2A. Please clearly indicate this in the respective figure legends.

- Regarding data quantification and statistics, please make sure that the number "n" for how many independent experiments were performed, their nature (biological versus technical replicates), the bars and error bars (e.g. SEM, SD) and the test used to calculate p-values is indicated in the respective figure legends (main, EV and Appendix figures). Please also check that all the p-values are explained in the legend, and that these fit to those shown in the figure. Please provide statistical testing where applicable. Please avoid the phrase 'independent experiment', but clearly state if these were biological or technical replicates. Please also indicate (e.g. with n.s.) if testing was performed, but the differences are not significant. In case n=2 please show the data as separate datapoints or bars without error bars and statistics (see e.g. Fig. 4B). See also:
http://www.embopress.org/page/journal/14693178/authorguide#statisticalanalysis

If n<5, please show single datapoints for diagrams.

- Please format the references according to our journal style:
http://www.embopress.org/page/journal/14693178/authorguide#referencesformat

The names should not be fully uppercase. If there are > 10 authors listed, please use et al. The year should be in brackets. It seem the reference Bodner et al. is missing the journal issue and page numbers.

- Please remove the reagents and tools table from the main manuscript text file. I have attached templates for that in word or excel format. Please upload the filled in table to the manuscript tracking system as 'Reagent Table' file. Please also adjust any callouts to this table. The example linked below shows how the table will display in the published article and includes examples of the type of information that should be provided for the different categories of reagents and tools. Please list your reagents/tools using the categories provided in the template and do not add additional subheadings to the table. Reagents/tools that do not fit in any of the specific categories can be listed under "Other":
https://www.embopress.org/pb%2Dassets/embo-site/msb_177951_sample_FINAL.pdf

- Please also complete the attached SD checklist and make sure that all the source data is provided.

- Finally, please find also attached a word file of the manuscript text (provided by our publisher) with changes we ask you to include in your final manuscript text and comments. Please use the attached file as basis for further revisions and provide your final manuscript file with track changes, in order that we can see any modifications done.

In addition, I would need from you:

- a short, two-sentence summary of the manuscript (not more than 35 words).
- two to four short (!) bullet points highlighting the key findings of your study (two lines each).
- a schematic summary figure as separate file that provides a sketch of the major findings (not a data image) in jpeg or tiff format (with the exact width of 550 pixels and a height of not more than 400 pixels) that can be used as a visual synopsis on our website.

I look forward to seeing the final revised version of your manuscript when it is ready. Please let me know if you have questions regarding the revision.

Best,

Achim Breiling
Senior Editor
EMBO Reports

------------
Referee #1:

I reviewed this manuscript from EMBO Journal. The authors have satisfied all of my concerns from then, and I recommend the work be accepted for publication.

------------
Referee #2:

The authors have addressed all remaining questions and the revised paper is suitable for publication.

The authors have addressed all minor editorial requests.

## 2nd Revision - Editorial Decision 14th Sep 2023

Prof. Johannes Huppa
Medical University of Vienna
Institute for Hygiene and Applied Immunology
Lazarettgasse 19
Vienna 1090
Austria

Dear Prof. Huppa,

I am very pleased to accept your manuscript for publication in the next available issue of EMBO reports. Thank you for your contribution to our journal.

At the end of this email I include important information about how to proceed. Please ensure that you take the time to read the information and complete and return the necessary forms to allow us to publish your manuscript as quickly as possible.

As part of the EMBO publication's Transparent Editorial Process, EMBO reports publishes online a Review Process File to accompany accepted manuscripts. As you are aware, this File will be published in conjunction with your paper and will include the referee reports, your point-by-point response and all pertinent correspondence relating to the manuscript.

If you do NOT want this File to be published, please inform the editorial office within 2 days, if you have not done so already, otherwise the File will be published by default [contact: emboreports@embo.org]. If you do opt out, the Review Process File link will point to the following statement: "No Review Process File is available with this article, as the authors have chosen not to make the review process public in this case."

Thank you again for your contribution to EMBO reports and congratulations on a successful publication. Please consider us again in the future for your most exciting work.

Yours sincerely,

Achim Breiling
Editor
EMBO Reports

**********************************************************************************

THINGS TO DO NOW:

Please note that you will be contacted by Wiley Author Services to complete licensing and payment information. The required 'Page Charges Authorization Form' is available here: https://www.embopress.org/pb-assets/embo-site/er_apc.pdf - please download and complete the form and return to embopressproduction@wiley.com

EMBO Press participates in many Publish and Read agreements that allow authors to publish Open Access with reduced/no publication charges. Check your eligibility: https://authorservices.wiley.com/author-resources/Journal-Authors/open-access/affiliation-policies-payments/index.html

You will receive proofs by e-mail approximately 2-3 weeks after all relevant files have been sent to our Production Office; you should return your corrections within 2 days of receiving the proofs.

Please inform us if there is likely to be any difficulty in reaching you at the above address at that time. Failure to meet our deadlines may result in a delay of publication, or publication without your corrections.

All further communications concerning your paper should quote reference number EMBOR-2023-57842V3 and be addressed to emboreports@wiley.com.

Should you be planning a Press Release on your article, please get in contact with emboreports@wiley.com as early as possible, in order to coordinate publication and release dates.

## EMBO Press Author Checklist

| | |
|---|---|
| Corresponding Author Name: Johannes Huppa | |
| Journal Submitted to: EMBO REPORTS | |
| Manuscript Number: EMBOR-2023-57842 | |

**USEFUL LINKS FOR COMPLETING THIS FORM**
The EMBO Journal - Author Guidelines
EMBO Reports - Author Guidelines
Molecular Systems Biology - Author Guidelines
EMBO Molecular Medicine - Author Guidelines

**Reporting Checklist for Life Science Articles (updated January**

This checklist is adapted from Materials Design Analysis Reporting (MDAR) Checklist for Authors. MDAR establishes a minimum set of requirements in transparent reporting in the life sciences (see Statement of Task: 10.31222/osf.io/9sm4x). Please follow the journal's guidelines in preparing your manuscript.

**Please note that a copy of this checklist will be published alongside your article.**

**Abridged guidelines for figures**

**1. Data**

The data shown in figures should satisfy the following conditions:

- the data were obtained and processed according to the field's best practice and are presented to reflect the results of the experiments in an accurate and unbiased manner.
- ideally, figure panels should include only measurements that are directly comparable to each other and obtained with the same assay.
- plots include clearly labeled error bars for independent experiments and sample sizes. Unless justified, error bars should not be shown for technical
- if n<5, the individual data points from each experiment should be plotted. Any statistical test employed should be justified.
- Source Data should be included to report the data underlying figures according to the guidelines set out in the authorship guidelines on Data

**2. Captions**

Each figure caption should contain the following information, for each panel where they are relevant:

- a specification of the experimental system investigated (eg cell line, species name).
- the assay(s) and method(s) used to carry out the reported observations and measurements.
- an explicit mention of the biological and chemical entity(ies) that are being measured.
- an explicit mention of the biological and chemical entity(ies) that are altered/varied/perturbed in a controlled manner.
- the exact sample size (n) for each experimental group/condition, given as a number, not a range;
- a description of the sample collection allowing the reader to understand whether the samples represent technical or biological replicates (including how many animals, litters, cultures, etc.).
- a statement of how many times the experiment shown was independently replicated in the laboratory.
- definitions of statistical methods and measures:
  - common tests, such as t-test (please specify whether paired vs. unpaired), simple χ2 tests, Wilcoxon and Mann-Whitney tests, can be unambiguously identified by name only, but more complex techniques should be described in the methods section;
  - are tests one-sided or two-sided?
  - are there adjustments for multiple comparisons?
  - exact statistical test results, e.g., P values = x but not P values < x;
  - definition of 'center values' as median or average;
  - definition of error bars as s.d. or s.e.m.

**Please complete ALL of the questions below.**
**Select "Not Applicable" only when the requested information is not relevant for your study.**

**Materials**

| Newly Created Materials | Information included in the manuscript? | In which section is the information available? (Reagents and Tools Table, Materials and Methods, Figures, Data Availability Section) |
|---|---|---|
| New materials and reagents need to be available; do any restrictions apply? | Yes | Reagents and Tools Table (p. 31- 37) and Material and Methods (p. 31 - 70); Appendix Figure S4; No restrictions apply. |

| Antibodies | Information included in the manuscript? | In which section is the information available? (Reagents and Tools Table, Materials and Methods, Figures, Data Availability Section) |
|---|---|---|
| For **antibodies** provide the following information:<br>- Commercial antibodies: RRID (if possible) or supplier name, catalogue number and or/clone number<br>- Non-commercial: RRID or citation | Yes | Reagents and Tools Table (p. 31 - 32) and Material and Methods (p. 46) |

| DNA and RNA sequences | Information included in the manuscript? | In which section is the information available? (Reagents and Tools Table, Materials and Methods, Figures, Data Availability Section) |
|---|---|---|
| **Short novel DNA or RNA including primers, probes**: provide the sequences. | Yes | Appendix Figure S4 |

| Cell materials | Information included in the manuscript? | In which section is the information available? (Reagents and Tools Table, Materials and Methods, Figures, Data Availability Section) |
|---|---|---|
| **Cell lines:** Provide species information, strain. Provide accession number in repository **OR** supplier name, catalog number, clone number, and/**OR** RRID. | Yes | Reagents and Tools Table (p. 35) and Material and Methods (p. 38-40) |
| **Primary cultures:** Provide species, strain, sex of origin, genetic modification status. | Yes | Material and Methods (p. 38-40) |
| Report if the cell lines were recently **authenticated** (e.g., by STR profiling) and tested for mycoplasma contamination. | Yes | Material and Methods (p. 40) |

| Experimental animals | Information included in the manuscript? | In which section is the information available? (Reagents and Tools Table, Materials and Methods, Figures, Data Availability Section) |
|---|---|---|
| **Laboratory animals or Model organisms:** Provide species, strain, sex, age, genetic modification status. Provide accession number in repository **OR** supplier name, catalog number, clone number, **OR** RRID. | Yes | Material and Methods (p. 38) |
| **Animal observed in or captured from the field:** Provide species, sex, and age where possible. | Not Applicable | |
| Please detail **housing and husbandry conditions**. | Yes | Material and Methods (p.38) |

| Plants and microbes | Information included in the manuscript? | In which section is the information available? (Reagents and Tools Table, Materials and Methods, Figures, Data Availability Section) |
|---|---|---|
| **Plants:** provide species and strain, ecotype and cultivar where relevant, unique accession number if available, and source (including location for collected wild specimens). | Not Applicable | |
| **Microbes:** provide species and strain, unique accession number if available, and source. | Yes | Reagents and Tools (p. 32) |

| Human research participants | Information included in the manuscript? | In which section is the information available? (Reagents and Tools Table, Materials and Methods, Figures, Data Availability Section) |
|---|---|---|
| If collected and within the bounds of privacy constraints report on age, sex and gender or ethnicity for all study participants. | Not Applicable | |

| Core facilities | Information included in the manuscript? | In which section is the information available? (Reagents and Tools Table, Materials and Methods, Figures, Data Availability Section) |
|---|---|---|
| If your work benefited from core facilities, was their service mentioned in the acknowledgments section? | Not Applicable | |

**Design**

| Study protocol | Information included in the manuscript? | In which section is the information available? (Reagents and Tools Table, Materials and Methods, Figures, Data Availability Section) |
|---|---|---|
| If study protocol has been **pre-registered, provide DOI in the manuscript**. For clinical trials, provide the trial registration number **OR** cite DOI. | Not Applicable | |
| Report the **clinical trial registration number** (at ClinicalTrials.gov or equivalent), where applicable. | Not Applicable | |

| Laboratory protocol | Information included in the manuscript? | In which section is the information available? (Reagents and Tools Table, Materials and Methods, Figures, Data Availability Section) |
|---|---|---|
| Provide DOI OR other citation details if **external detailed step-by-step protocols** are available. | Not Applicable | |

| Experimental study design and statistics | Information included in the manuscript? | In which section is the information available? (Reagents and Tools Table, Materials and Methods, Figures, Data Availability Section) |
|---|---|---|
| Include a statement about **sample size** estimate even if no statistical methods were used. | Yes | Figure and EV Figure legends (p.81-117) |
| Were any steps taken to minimize the effects of subjective bias when allocating animals/samples to treatment (e.g. **randomization procedure**)? If yes, have they been described? | Yes | Material and Methods (p. 38), Figure and EV Figure legends (p.81-117) |
| Include a statement about **blinding** even if no blinding was done. | Not Applicable | |
| Describe **inclusion/exclusion criteria** if samples or animals were excluded from the analysis. Were the criteria pre-established?<br><br>If sample or data points were omitted from analysis, report if this was due to attrition or intentional exclusion and provide justification. | Not Applicable | |
| For every figure, are **statistical tests** justified as appropriate? Do the data meet the assumptions of the tests (e.g., normal distribution)? Describe any methods used to assess it. Is there an estimate of variation within each group of data? Is the variance similar between the groups that are being statistically compared? | Yes | Figures and Figure legends (p. 81-117) |

| Sample definition and in-laboratory replication | Information included in the manuscript? | In which section is the information available? (Reagents and Tools Table, Materials and Methods, Figures, Data Availability Section) |
|---|---|---|
| In the figure legends: state number of times the experiment was **replicated** in laboratory. | Yes | Figure and EV Figure legends (p.81-117) |
| In the figure legends: define whether data describe **technical or biological replicates**. | Yes | Figure and EV Figure legends (p.81-117) |

## Ethics

| Ethics | Information included in the manuscript? | In which section is the information available? (Reagents and Tools Table, Materials and Methods, Figures, Data Availability Section) |
|---|---|---|
| Studies involving **human participants**: State details of **authority granting ethics approval** (IRB or equivalent committee(s), provide reference number for approval. | Not Applicable | |
| Studies involving **human participants**: Include a statement confirming that **informed consent** was obtained from all subjects and that the experiments conformed to the principles set out in the WMA Declaration of Helsinki and the Department of Health and Human Services Belmont Report. | Not Applicable | |
| Studies involving **human participants:** For publication of **patient photos**, include a statement confirming that consent to publish was obtained. | Not Applicable | |
| Studies involving experimental **animals**: State details of **authority granting ethics approva**l (IRB or equivalent committee(s), provide reference number for approval. Include a statement of compliance with ethical regulations. | Yes | Material and Methods (p. 38) |
| Studies involving **specimen and field samples:** State if relevant **permits** obtained, provide details of authority approving study; if none were required, explain why. | Not Applicable | |

| Dual Use Research of Concern (DURC) | Information included in the manuscript? | In which section is the information available? (Reagents and Tools Table, Materials and Methods, Figures, Data Availability Section) |
|---|---|---|
| Could your study fall under dual use research restrictions? Please check biosecurity documents and list of **select agents and toxins** (CDC): https://www.selectagents.gov/sat/list.htm | Not Applicable | |
| If you used a select agent, is the security level of the lab appropriate and reported in the manuscript? | Not Applicable | |
| If a study is subject to dual use research of concern regulations, is the name of the **authority granting approval and reference number** for the regulatory approval provided in the manuscript? | Not Applicable | |

## Reporting

The MDAR framework recommends adoption of discipline-specific guidelines, established and endorsed through community initiatives. Journals have their own policy about requiring specific guidelines and recommendations to complement MDAR.

| Adherence to community standards | Information included in the manuscript? | In which section is the information available? (Reagents and Tools Table, Materials and Methods, Figures, Data Availability Section) |
|---|---|---|
| State if relevant guidelines or checklists (e.g., **ICMJE, MIBBI, ARRIVE, PRISMA**) have been followed or provided. | Yes | Material and Methods (p. 38) |
| For **tumor marker prognostic studies,** we recommend that you follow the **REMARK** reporting guidelines (see link list at top right). See author guidelines, under 'Reporting Guidelines'. Please confirm you have followed these guidelines. | Not Applicable | |
| For **phase II and III randomized controlled trials**, please refer to the **CONSORT** flow diagram (see link list at top right) and submit the CONSORT checklist (see link list at top right) with your submission. See author guidelines, under 'Reporting Guidelines'. Please confirm you have submitted this list. | Not Applicable | |

## Data Availability

| Data availability | Information included in the manuscript? | In which section is the information available? (Reagents and Tools Table, Materials and Methods, Figures, Data Availability Section) |
|---|---|---|
| Have **primary datasets** been deposited according to the journal's guidelines (see 'Data Deposition' section) and the respective accession numbers provided in the Data Availability Section? | Yes | Data availability section (p. 71) |
| Were **human clinical and genomic datasets** deposited in a public access-controlled repository in accordance to ethical obligations to the patients and to the applicable consent agreement? | Not Applicable | |
| Are **computational models** that are central and integral to a study available without restrictions in a machine-readable form? Were the relevant accession numbers or links provided? | Yes | Material and Methods (p. 54 - 70). Links to the software packages for analysis can be obtained from the cited papers or from the corresponding authors. |
| If publicly available data were reused, provide the respective **data citations in the reference list**. | Not Applicable | |
